# Flavone-based dual PARP-Tubulin inhibitor manifesting efficacy against endometrial cancer

**DOI:** 10.1080/14756366.2023.2276665

**Published:** 2023-11-02

**Authors:** Sachin Sharma, Kavya Chandra, Aliva Naik, Anamika Sharma, Ram Sharma, Amandeep Thakur, Ajmer Singh Grewal, Ashwani K. Dhingra, Arnab Banerjee, Jing Ping Liou, Santosh Kumar Guru, Kunal Nepali

**Affiliations:** a School of Pharmacy, College of Pharmacy, Taipei Medical University, Taipei, Taiwan; b Department of Biological Sciences, BITS Pilani KK Birla Goa campus, Goa, India; c Department of Biological Sciences, National Institute of Pharmaceutical Education and Research, Hyderabad, India; d Guru Gobind Singh College of Pharmacy, Yamuna Nagar, India; e Program in Drug Discovery and Development Industry, College of Pharmacy, Taipei Medical University, Taipei, Taiwan

**Keywords:** Flavone, benzopyran, PARP, tubulin, inhibitor, endometrial cancer

## Abstract

Structural tailoring of the flavone framework (position 7) via organopalladium-catalyzed C–C bond formation was attempted in this study. The impact of substituents with varied electronic effects (phenyl ring, position 2 of the benzopyran scaffold) on the antitumor properties was also assessed. Resultantly, the efforts yielded a furyl arm bearing benzopyran possessing a 4-fluoro phenyl ring (position 2) (**14**) that manifested a magnificent antitumor profile against the Ishikawa cell lines mediated through dual inhibition of PARP and tubulin [(IC_50_ (PARP1) = 74 nM, IC_50_ (PARP2) = 109 nM) and tubulin (IC_50_ = 1.4 µM)]. Further investigations confirmed the ability of **14** to induce apoptosis as well as autophagy and cause cell cycle arrest at the G2/M phase. Overall, the outcome of the study culminated in a tractable dual PARP-tubulin inhibitor endowed with an impressive activity profile against endometrial cancer.

## GRAPHICAL ABSTRACT

**Figure:**
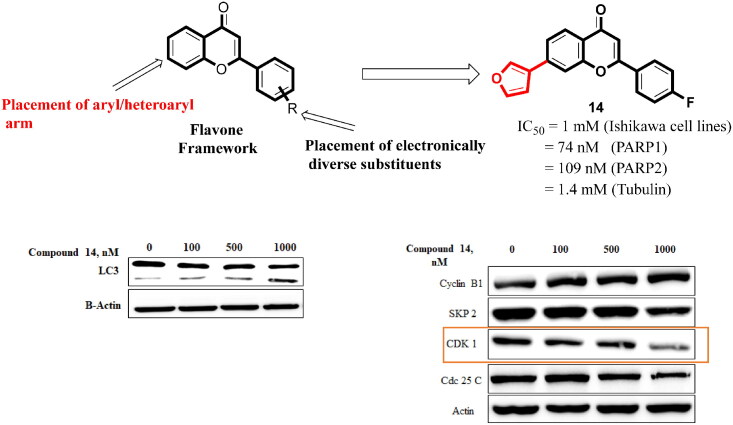

## 1. Introduction

Cancer is a multifactorial and deadly disease^1–3^ characterised by the development of uncontrollably dividing abnormal cells that infiltrate and destroy normal body tissue.^4–6^ One of the major areas of concern in the category of gynaecologic cancers, is endometrial cancer, with over 60 000 new cases diagnosed per year in the United States alone.^7–16^ Endometrial cancer starts in the endometrium (lining of the uterus) and is mostly observed in postmenopausal women, with few cases occurring in younger women. The aetiology of endometrial cancer includes genetic and epigenetic alterations.^17–23^ Curative surgery including total hysterectomy and bilateral salpingo-oophorectomy is the recommended treatment strategy for early-stage endometrial cancer. For the advanced stage of endometrial cancer, reliance on chemotherapy is a prudent choice.^24^ Disappointingly, treatment options for advanced endometrial cancer are marred by a lack of new targeted agents and it is conceived that supplementing the armoury of anticancer interventions for endometrial cancer via logical fabrication of scaffolds is the need of the hour.

Literature precedents indicate that recent attempts by medicinal chemists in the field of new anti-cancer drug development have been directed towards the design of dual-targeting inhibitors.^24–29^ This inclination is attributed to the synergistic antiproliferation effects evidenced by the “one scaffold – two targets approach”. Noteworthy to mention that resorting to a cocktail of drugs (two drugs two targets) is also a tried-tested option to attain amplified antitumor effects. However, multifunctional chemical architectures score over combination therapy owing to the issues associated with the use of two chemotherapeutic drugs (combination therapy) viz., undesirable drug − drug interactions, complicated pharmacokinetics, and intricate toxicity profiles.^26–29^ Given the aforementioned, the design of dual inhibitors was envisioned as a logical stratagem to load the anti-endometrial cancer chemical toolbox in this study.

Albeit the approach of furnishing multifunctional therapeutics for cancer appears to be fascinating, the selection of the targets that can be modulated simultaneously or concomitantly to attain enhanced anti-tumour efficacy is a critical and daunting task.^30–31^ Delightfully, an extensive literature survey led us to arrive at the candidature of PARP and tubulin as druggable targets for the design of new structural assemblages for endometrial cancer.^30–34^

Poly (ADP-ribose) polymerases (or PARPs) are a family of proteins involved in DNA damage repair.^35^^,^^36^ Amongst the various members, PARP1 and PARP2 are major enzymes that regulate the DNA damage response through the process of parylation (transfer of PAR chains from the nicotinamide-adenine-dinucleotide).^36^ Various scientific reports have been published that indicate that the increased PARP levels in a variety of cancer cells lead to genomic instability, resistance of cells towards death, replicative immortality, and reprogrammed metabolism.^37^ The last decade witnessed the FDA approvals of several PARP inhibitors for the treatment of BRCA mutations harbouring cancer viz. Olaparib for breast cancer, niraparib for ovarian cancer, rucaparib for pancreatic cancer, and talazoparib for prostate cancer. Other than the aforestated inhibitors, fuzuloparib, and pamiparib have received CFDA approvals. Veliparib, stenoparib, mefuparib, and RBN-2397 represent the investigational small molecule PARP inhibitors.^30^ Much to the delight, recent disclosures perspicuously underscore the favourable trends witnessed with PARP inhibitors in endometrial cancer such as: (i) the promising efficacy of olaparib in advanced endometrial carcinoma^31^ (ii) pathologic complete response of high-grade endometrial cancer to PARP inhibitors^32^ (iii) optimistic activity profile of a triplet regimen consisting of PARP inhibitor in recurrent, advanced endometrial carcinoma^33^ (iv) manifestation of efficacy by olaparib, in cultured endometrial carcinoma cells.^34^ These outcomes clearly advocate for the targeting of PARPs in endometrial cancer.

Along with PARP, Tubulin was pinpointed as a promising target for endometrial cancer.^38–40^ As such, tubulin is a basic building block of microtubules, the cytoskeleton protein polymer, involved in cell structure maintenance, intracellular organelle transportation, and distribution of cells during the cell cycle.^40–42^ The last three decades have evidenced an explosion of research on tubulin inhibitors that has led to the furnishment of numerous chemical architectures eliciting efficacy in diverse malignancies. Extensive research in this direction has resulted in some FDA approvals, along with the emergence of many investigational drugs. Encouragingly, tubulin inhibitor AEZS 112 was reported to inhibit the growth of experimental human ovarian and endometrial cancers.^43^ In addition, tubulin inhibitors as monotherapy, as well as a part of combination therapy, are being evaluated in clinical trials for the treatment of endometrial cancer (NCT03981796). Overall, the progress chart of tubulin inhibitors has validated tubulin as one of the most sought-after targets for cancer chemotherapy.

In addition to the reports of the efficacy of PARP and tubulin in endometrial cancer (monotherapy), some precedents advocate for a more pronounced efficacy of a combination of PARP and tubulin inhibitors ascertaining the benefits of simultaneous modulation of both targets. A study outcome demonstrated that a cocktail of PARP inhibitors with paclitaxel decreased endometrial cancer cell viability.^44^ Also, PARP inhibitors sensitised endometrial cancer cells to cytotoxic treatment with paclitaxel.^44^ Moreover, a combination of PARP inhibitor AZD5305 with paclitaxel is currently being assessed in endometrial cancer in a clinical trial (NCT04644068). Also, a recent study outcome demonstrated the striking antitumor efficacy of a small molecule dual PARP-tubulin inhibitor against various cancers.^45^ The aforementioned shreds of evidence clubbed with assertions claiming that multifunctional scaffolds can eliminate the need for extensive investigations required for combination therapy, the theoretical basis of the present study was formulated to design dual PARP-Tubulin inhibitors, anticipating promising efficacy of the adducts in endometrial cancer.

## 2. Drug design

**Figure 1.**
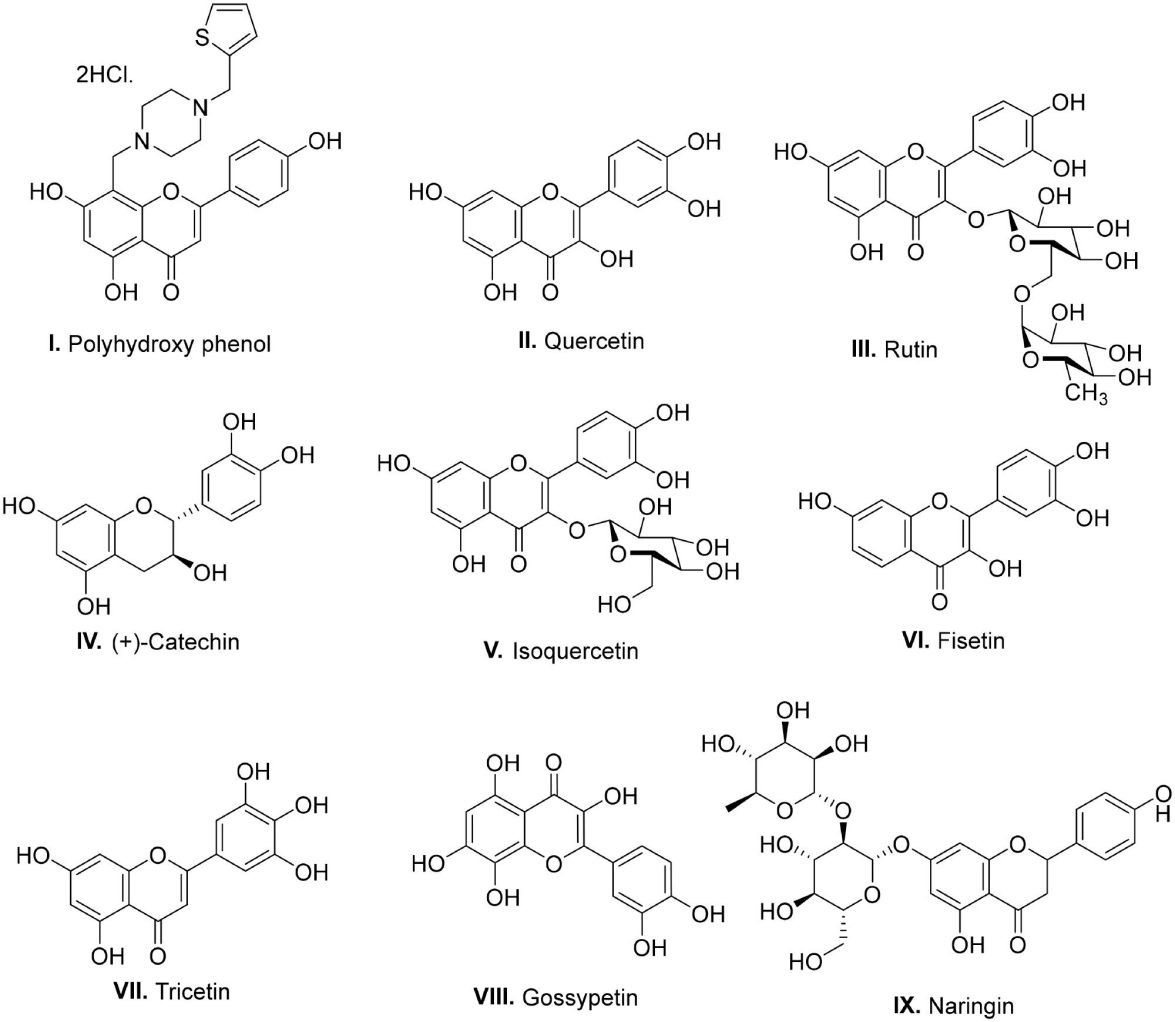
Reported flavonoid-based PARP inhibitors.

With a clear cut-idea in hand to design dual PARP-tubulin inhibitors as prospective therapeutics for endometrial cancer, our drug discovery team commenced with the screening of the synthetic bank of the laboratory for pinpointing adducts endowed with dual modulatory ability towards PARP and tubulin. Noteworthy to mention that lately, our medicinal chemistry campaigns have focused on the structural engineering of alkaloids and flavonoids as well as the synthesis of natural product-inspired libraries of bioactive scaffolds. Delightfully, the screening results led us to arrive at the structural template of biaryl-type flavones that were previously furnished by our research group to amplify the tubulin inhibitory potential of flavones. Unfortunately, the endeavour was halted as the generated templates did not elicit an amplified tubulin polymerisation inhibitory activity, however, retention/modest reduction in tubulin inhibition was observed with most of the structures (unpublished work). Excitingly, in the screening assay, the prototype compound of the biaryl-type flavones demonstrated moderate potential to inhibit PARP1 and PARP2. This preliminary screening result is aligned with several disclosures ascertaining the PARP inhibitory potential of flavone-based adducts (Figure 1).^46–49^ Given the optimistic direction, we conceived structural tailoring of biaryl-type flavones might culminate into a tractable anti-endometrial cancer agent mediating efficacy via dual PARP1/PARP2-tubulin inhibition.

**Figure 2.**
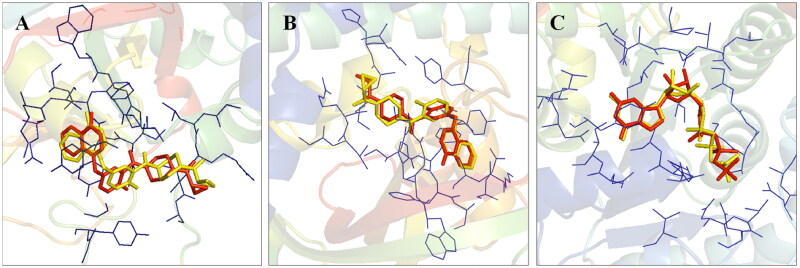
Validation of the docking protocol. The docking protocol was validated via redocking the co-crystallized ligands. The re-docked ligands (yellow) produced a pose similar to those of the co-crystallized ligands (red) (A- PARP1; B- PARP2 and C- Tubulin).

Before the commencement of the task to design and generate a compendium of biaryl-type flavones, a docking study of prototype biaryl flavone **1** was conducted within the active site of PARP1, PARP2, and tubulin. The docking protocol was first validated by redocking the respective co-crystallized ligands with PARP1, PARP2, and tubulin proteins (Figure 2). The re-docked ligands/inhibitors of these proteins (PARP1, PARP2, and tubulin) produced poses similar to the co-crystallized ligands/inhibitors with PARP1, PARP2 and tubulin (PDB IDs: 5DS3, 4TVJ and 1SA0, respectively), indicating that a rational docking protocol was used in this study. The docking score (binding free energy) as well as residues involved in hydrogen bonding and hydrophobic interactions are presented in Table 1.

**Table 1.** Docking score and residues involved in binding interactions of compound **1** with PARP1, PARP2, and tubulin.

Compound	Docking score (kcal/mol)	Residues involved in hydrogen bonds (bond distance)	Residues involved in hydrophobic & other interactions
PARP1 (PDB ID: 5DS3)			
**1**	−8.7	–	His862, Ala880, Tyr896, Ala898, Lys903, Tyr907
PARP2 (PDB ID: 4TVJ)			
**1**	−9.5	–	Ala446, Tyr462, Ala464, Lys469, Tyr473
Tubulin (PDB ID: 1SA0)			
**1**	−8.7	–	Gln11, Ala12, Ala99, Tyr224

**Figure 3.**
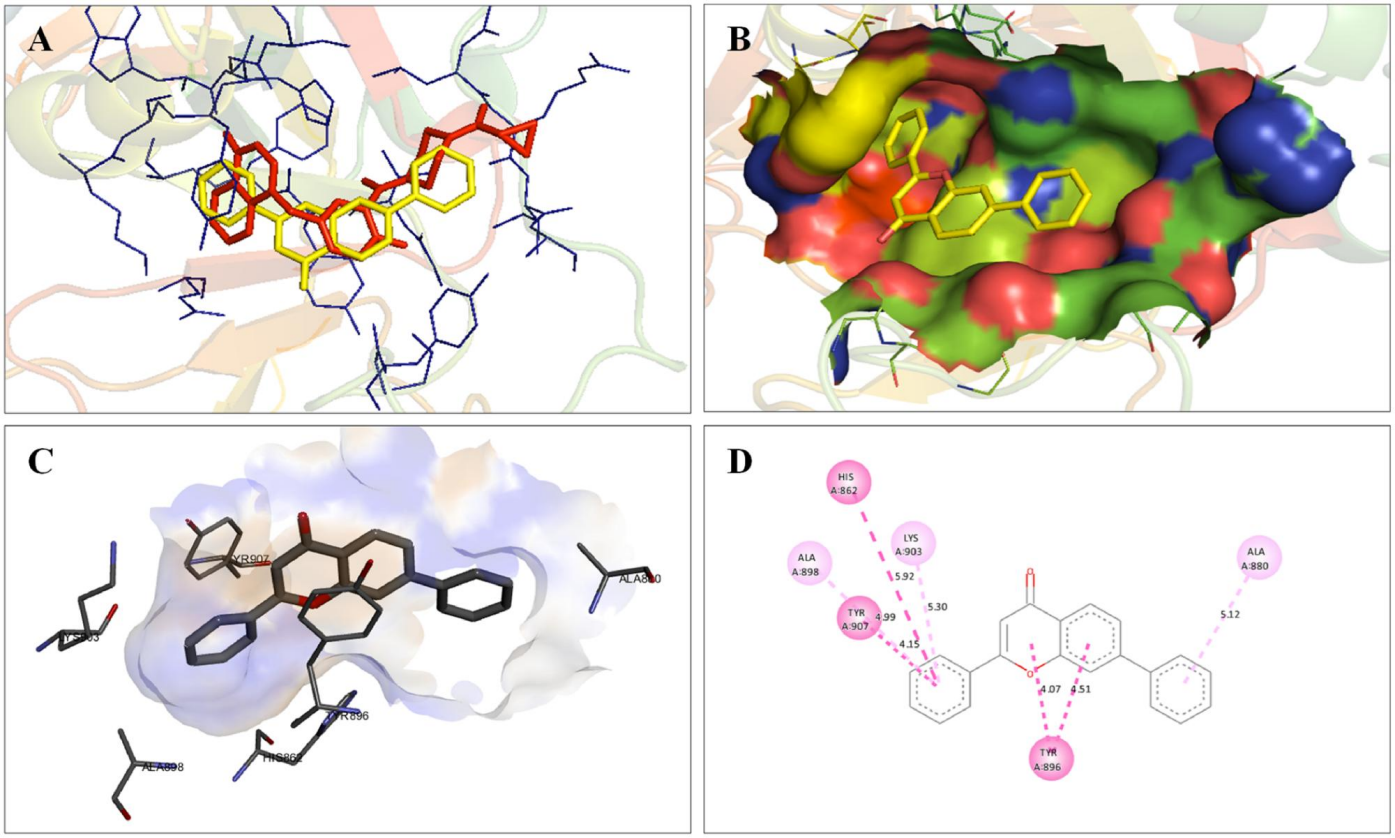
Interaction analysis of **1** with PARP1. (A) Overlay of **1** (yellow) with co-crystallized ligand (red). (B) Orientation of **1** in the active site. (C) 3D docked pose of **1**. (D) 2D docked pose of **1** showing hydrophobic interactions with PARP1.

**Figure 4.**
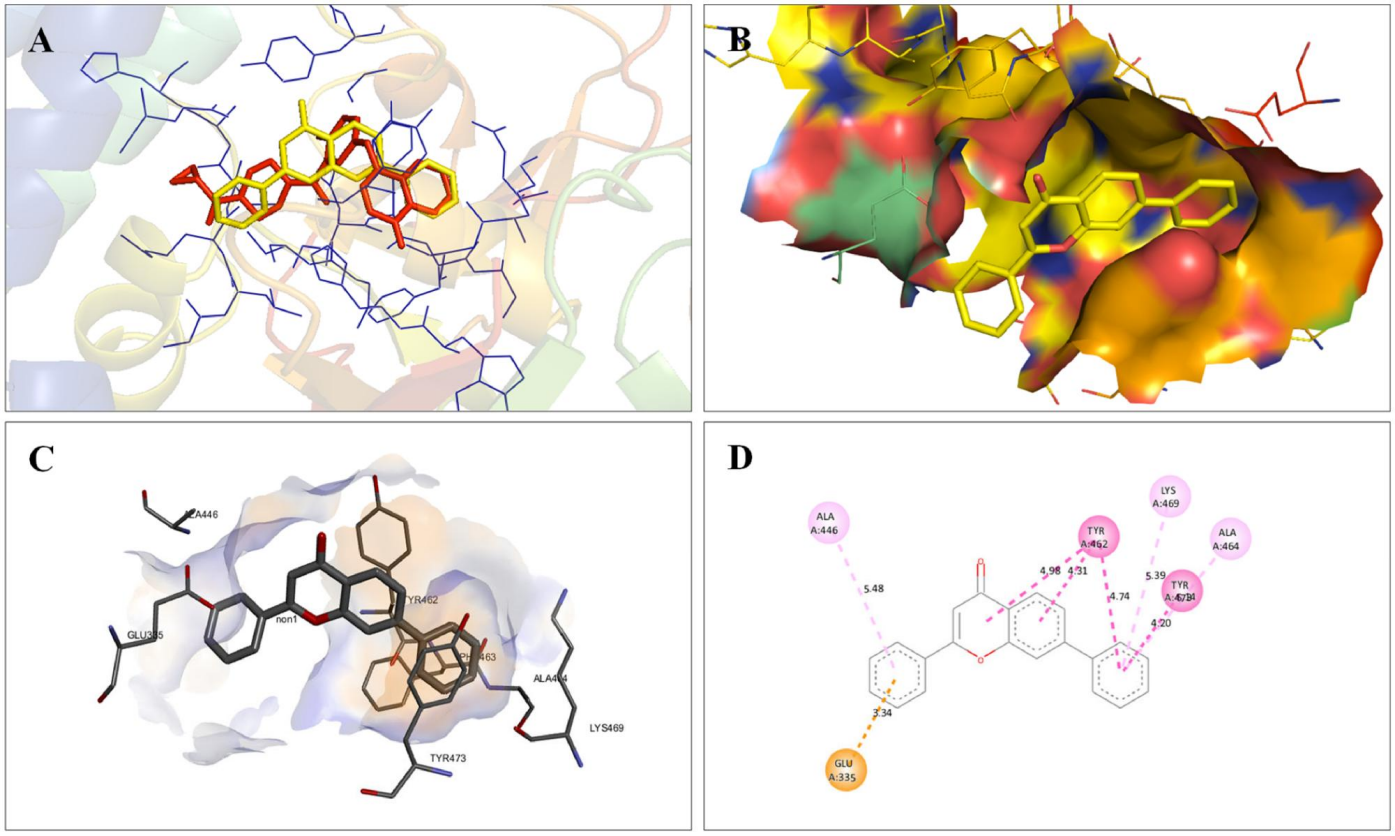
Interaction analysis of **1** with PARP2. (A) Overlay of **1** (yellow) with co-crystallized ligand (pink). (B) Orientation of **1** in the active site of PARP2 protein. (C) 3D docked pose of **1**. (D) 2D docked pose of **1** showing hydrophobic interactions.

**Figure 5.**
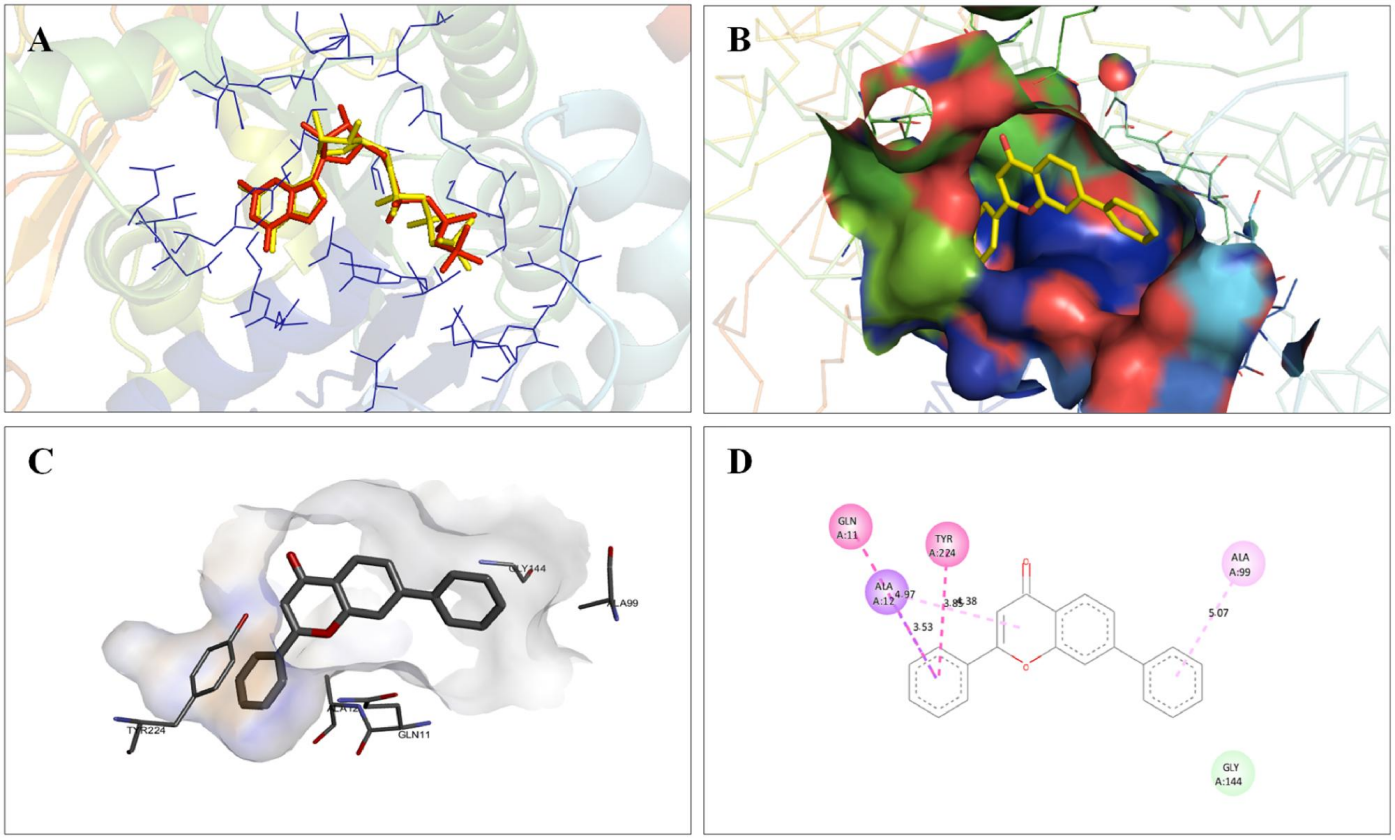
Interaction analysis of **1** with tubulin. (A) Overlay of **1** (yellow) with co-crystallized ligand (pink). (B) Orientation of **1** in the active site of tubulin. (C) 3D docked pose of **1**. (D) 2D docked pose of **1** showing hydrophobic interactions with tubulin.

The docking interactions of compound **1** with active site residues of PARP1 (PDB ID: 5DS3) are shown in Figure 3. Compound **1** exhibited hydrophobic interactions with His862, Tyr896 & Tyr907 residues (pi-pi type) and Ala880, Ala898 & Lys903 residues (pi-alkyl type) in the active site of PARP1 protein. Figure 4 shows the docking interactions of compound **1** with active site residues of PARP2 (PDB ID: 4TVJ). Hydrophobic interactions with Tyr462 & Tyr473 residues (pi-pi type), Ala446, Ala464 & Lys469 residues (pi-alkyl type), and electrostatic interaction with Glu335 residue (pi-anion type) in the active site of PARP2 protein were observed. The interaction profile of compound **1** with the amino acid residues of tubulin (PDB ID: 1SA0) is depicted in Figure 5. The prototype flavone manifested hydrophobic interactions with Gln11 & Tyr224 residues (pi-pi type), Ala12 & Ala99 residues (pi-alkyl type) and Ala12 residue (pi-sigma type) in the active site of tubulin protein.

**Figure 6.**
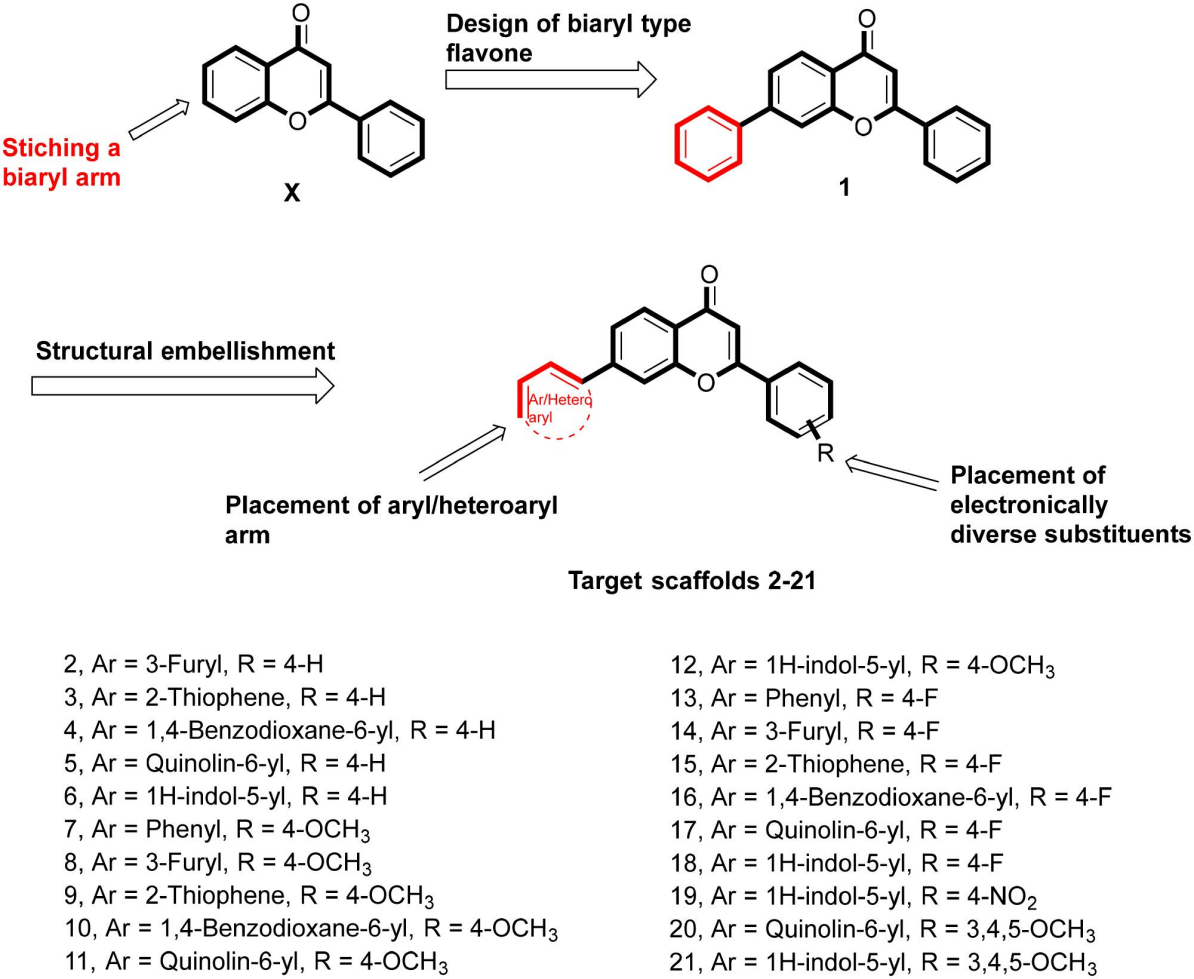
Design strategy.

Overall, the docking results indicated that flavone **1** was well accommodated within the active site of PARP1, PARP2, and tubulin. Based on the interaction profile of flavone **1,** it was conceived that the structural embellishment of the flavone framework might lead to additional interactions (hydrogen bonding and others) with the amino acid residues of the targets, thereby enhancing the enzyme inhibitory activity. With this background, a series of biaryl flavones was accomplished as depicted in Figure 6 and subjected to a series of biological evaluation assays. Encouragingly, a dual PARP-tubulin inhibitor **14** endowed with magnificent cellular activity against the endometrial cancer cell lines was identified in the present study. The bifunctional adduct demonstrated the potential to induce apoptosis as well as autophagy and caused cell cycle arrest at the G2-M phase.

**Figure 7.**
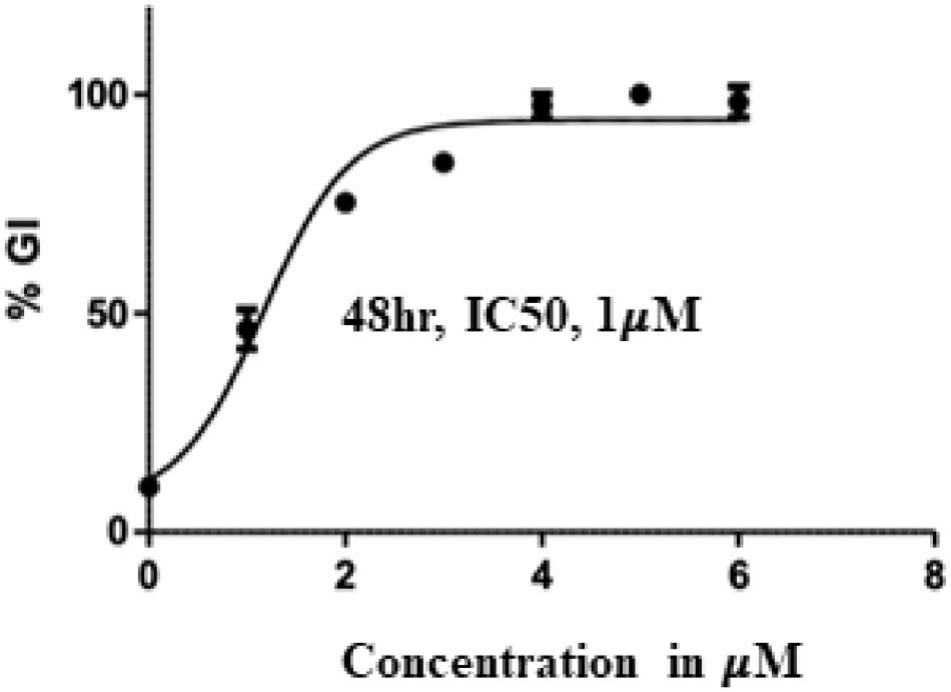
IC_50_ value of compound **14 (**Treatment concentrations- 2,4,6 and 8 µM).

## 3. Results and discussions

### 3.1. Synthesis

**Scheme 1.**
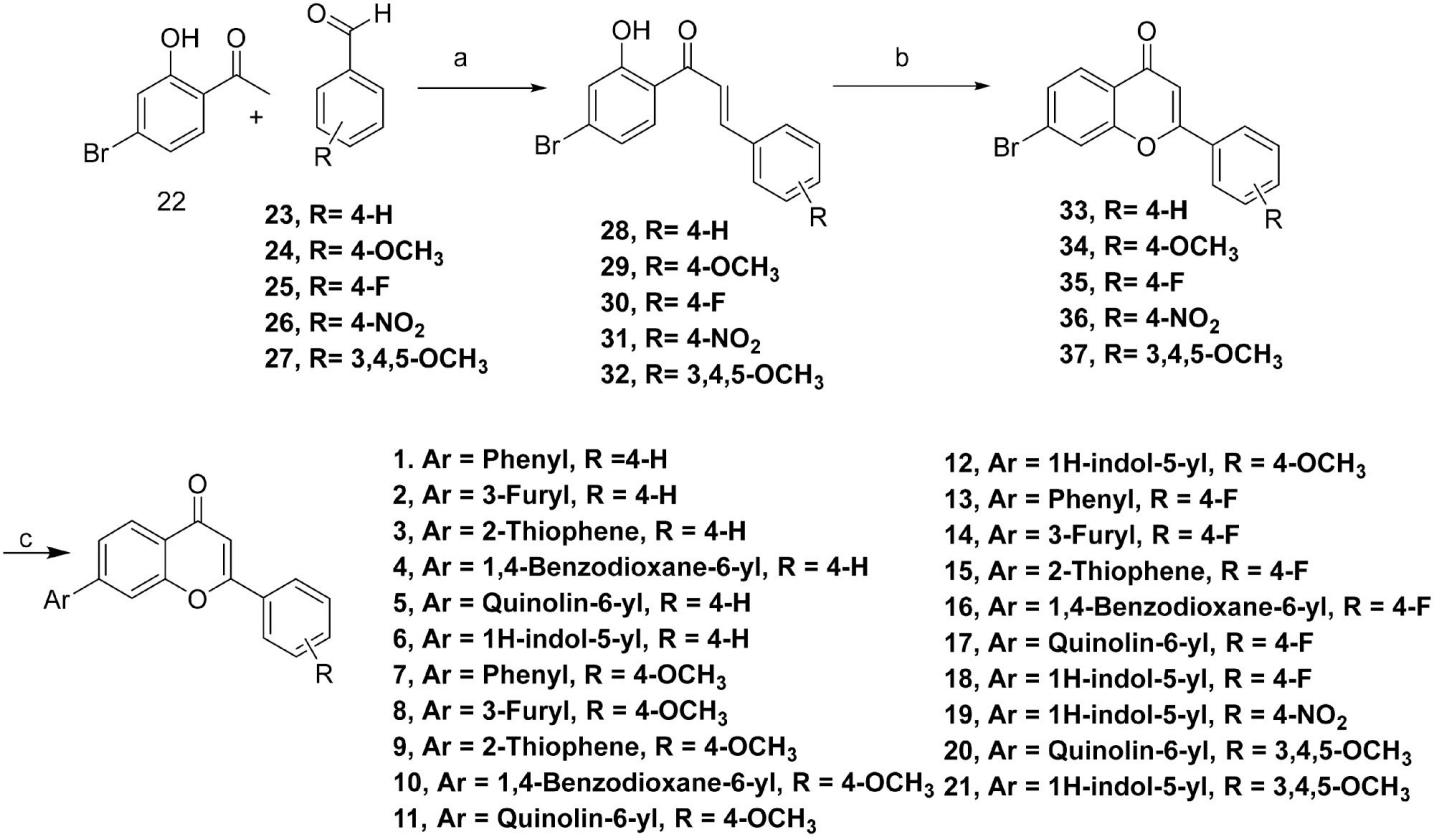
Reagents and conditions: (A) NaOH, Ethanol, rt, 24h; (B) I_2_, DMSO, reflux, 24 Wh; (C) aryl/heteroaryl boronic acids, Pd(PPh_3_), Dioxane: Water (9:3), reflux, 100 °C, 2h.

The designed compounds (**1–21**) were furnished via a multistep synthetic route depicted in Scheme 1. Commercially available aromatic aldehydes and ketones with diverse substitutions were used for the base-mediated Claisen Schmidt condensation to afford intermediates **28–32**. Iodine-mediated cyclisation of intermediates **28–32** yielded the benzopyrans (**33–37**). Notably, the absence of proton resonance (β to carbonyl) in intermediates (**33–37**) confirmed the intramolecular attack of the hydroxy group at the β carbon leading to the cyclisation of adducts **28–32**. Forth, the benzopyrans (**33–37**) were subjected to C-C bond formations via Suzuki arylation employing diverse boronic acids to obtain the target structural assemblages (**1–21**). Overall, the established synthetic protocol was robust and efficient and afforded the synthesis of target compounds in moderate to excellent yields. Imperative to mention that the Suzuki arylation methodology enabling C-C bond formations at position 7 of the benzopyran skeleton was flexible enough to enable the installation of aryl as well as heteroaryl arms.

### 3.2. In-vitro cytotoxicity studies

The cell growth inhibitory effects of the synthesised compounds were evaluated against Ishikawa cell lines. Comparisons were done with flavone **X**, biaryl flavone **1, olaparib (FDA-approved PARP inhibitor), and combretastatin (tubulin inhibitor)** (Table 2). The assessment commenced with the screening of compound **1** which elicited moderate cell growth inhibitory potential against the employed cell line with an IC_50_ value of 3 µM. This observation depicted that stitching of the aryl ring on the flavone framework conferred cell growth inhibitory potential to the resulting scaffolds (compare **X** with **1**). Further, analysis was done to compare the cytotoxicity of compound **1** with other substituted benzopyrans (compounds **2–6**). The outcome perspicuously revealed that the Suzuki arylation to afford C–C bond formation on the benzopyran framework led to the amplification of the cell growth inhibitory effects only in cases where benzopyrans were appended to a monocyclic heteroaryl ring (**2, 3**). Notably, biaryls **2** and **3** demonstrated more pronounced cytotoxic effects than their counterpart **1** with IC_50_ values of 1.5 and 2.1 µM, respectively. The higher cell growth inhibitory effects of biaryl **2** bearing a furyl ring could be attributed to the tendency of oxygen to form stronger hydrogen bonds. However, contrary to the expectations, the diametrically opposite impact of tethering a sterically bulky biaryl arm on the benzopyran skeleton was observed as biaryl flavones **4–6** demonstrated cytotoxicity-devoid trends. This indicated that the enhancement of steric bulk at the benzopyran framework (**position 7**) was not tolerable. Forth, the synthesised compounds bearing a substituted phenyl ring at **position 2** of the biaryl arm bearing benzopyrans were profiled for the cytotoxic effects. Noteworthy to mention that diverse substitutions with varied electronic effects viz fluorine (−I, +R), methoxy (−I, +R), and nitro (−I and − R) were made on the phenyl ring (**position 2** of benzopyran). Also, the trimethoxy phenyl ring well established as an imperative structural feature of tubulin inhibitors was tethered to the benzopyran scaffold (**20, 21**). Unfortunately, none of the substitution patterns other than fluorine substitution paid dividends in the context of substantial cell growth inhibitory effects.

**Table 2.** *In-vitro* growth inhibition (IC_50_ in µM) effects of the compounds (**1–21**).

Compound code	IC_50_ values (Ishikawa cell lines, µM ± SD^a^)
1	3 ± 1.09
2	1.5 ± 0.67
3	2.1 ± 0.89
4	>10
5	>10
6	>10
7	6.3 ± 1.08
8	3.2 ± 1.04
9	3 ± 0.785
10	>10
11	>10
12	>10
13	2.03 ± 1.10
**14**	**1.0 ± 0.40**
15	1.5 ± 1.08
16	>10
17	>10
18	>10
19	>10
20	>10
21	>10
**X**	>10
**Olaparib**	3.91 ± 1.06
**Combretastatin**	4.21 ± 0.78

^a^SD: standard deviation, all experiments were independently performed at least three times

Encouragingly, Flavones **13, 14,** and **15** bearing a monocyclic aryl/heteroaryl arm (**position 7**) and a 4-fluorophenyl ring at position **2** elicited remarkable cytotoxicity profiles with IC_50_ values of 2.03, 1.00 and 1.5 µM, respectively. Indeed, a careful comparison of the cell growth inhibitory effects of counterparts **1** and **13**, **2** and **14**, **3** and **15** perspicuously highlights the favourable trends of placing fluorine at the para position of the phenyl ring (**position 2 of benzopyran scaffold**). Each of the fluorine-bearing adducts outshone its non-fluorine-bearing counterpart with lower IC_50_ values. Noteworthy to mention that these trends were not replicated by fluorine-bearing adducts **16**, **17,** and **18** ascertaining the non-preference of the benzopyran towards bicyclic aromatic/heteroaromatic rings (**position 7**) and biasedness towards the inclusion of monoaryl/heteroaryl arms in their framework. Taken together, compound **14** was pinpointed as the most promising cell growth inhibitor against Ishikawa cell lines (Figure 7) and was further investigated exhaustively.

### 3.3. Compound 14 inhibits PARP1 and PARP2

**Figure 8.**
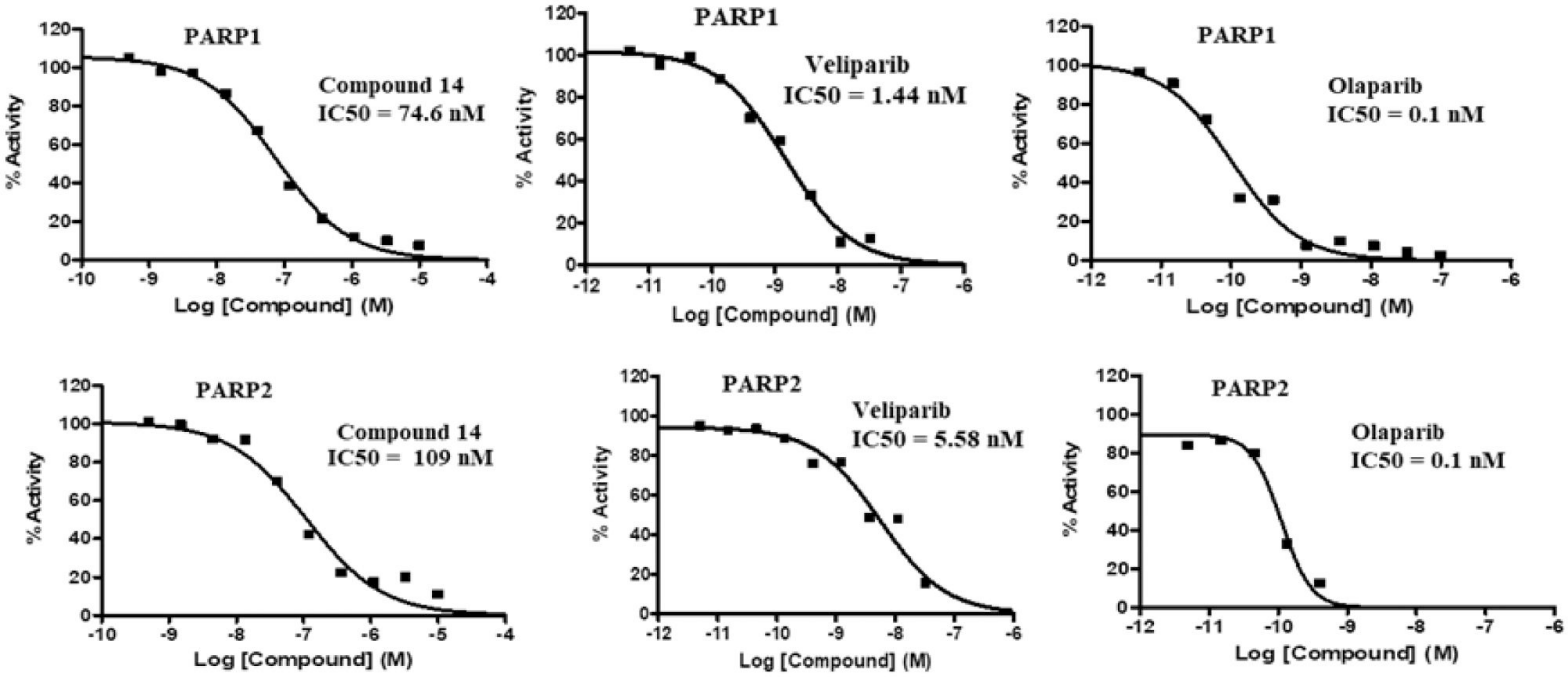
IC_50_ values (PARP inhibition) of compound **14,** Olaparib and Veliparib.

Compound **14** was evaluated for its ability to inhibit PARP1 and PARP2. As such, PARP enzymatic activity transfers ADP-ribose from NAD + substrate into the PAR chain on histone substrate. The amount of NAD + remaining after the enzymatic activity was quantified using the Promega NAD/NADH-Glo™ assay kit, in the endpoint PARP activity assay. The compounds were tested in a 10-dose IC_50_ singlet with a 3-fold serial dilution starting at 10 µM against 2 PARPs. The control compound, Veliparib (ABT-888, PARP inhibitor), was tested in a 10-dose IC_50_ with a 3-fold serial dilution starting at 0.1 uM. The outcome presented in Table 3 and Figure 8 depicts the striking inhibitory potential of compound **14** against PARP1 and PARP2 with an IC_50_ value of 74 nM and 109 nM. These results are highly indicative of PARP inhibition as one of the mechanisms underlying the cytotoxicity of compound **14** against the Ishikawa cell lines.

**Table 3.** PARP1 and PARP2 inhibitory activity of compound 14.

Compound	IC_50_ (nM)	
	PARP1	PARP2
14	74.6	109
Olaparib	0.1	0.11
Veliparib (ABT-888)	1.44	5.58

### 3.4. In-vitro tubulin inhibition

Compound **14** was further profiled for its tubulin inhibitory effects and the results are illustrated in Table 4. Combretastatin A-4 was employed as a standard tubulin inhibitor in this study. Encouragingly, flavone **14** manifested magnificent tubulin inhibitory potential with an IC_50_ value of 1.4 µM. Notably, a correlation of the results presented in Tables 2–4 clearly reflects that the striking cell growth inhibitory effects of compound **14** stems from its ability to exert dual inhibition of PARP and Tubulin.

**Table 4.** Tubulin inhibitory activity of compound **14** (Treatment concentrations – 0.1, 1, 5, 10 μM).

Compound	IC_50_ (µM, Tubulin polymerisation)^a^
**14**	1.4 ± 0.3
**CA-4**	1.3 ± 0.7

^a^IC_50_ values were shown as the mean ± SD of three independent experiments

### 3.5. Compound 14 induces apoptosis (nuclear staining)

**Figure 9.**
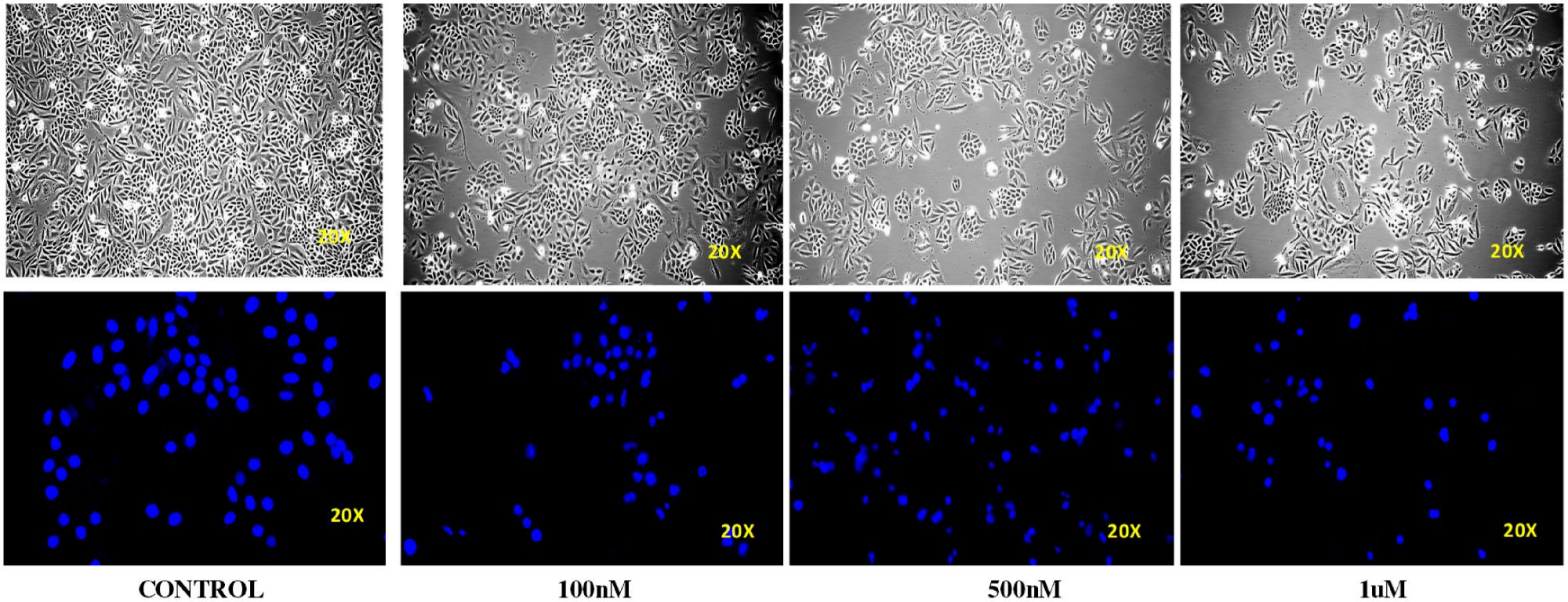
Effect of compound **14** on the cell wall and nuclear morphology of Ishikawa cells.

Ishikawa cell lines were treated with compound **14** at concentrations of 0.1, 0.5, and 1 μM for 24 h. Cellular morphology was observed by an inverted microscope. DAPI staining was done to monitor the nuclear morphology. It was observed that treatment with compound **14** led to a concentration-dependent increase in the number of scattered apoptotic bodies in Ishikawa cells. In addition, cell size shrinkage, nuclear condensation and cell wall deformation were observed in the morphology of treated cells. Thus, the results were indicative of the apoptosis-inducing ability of compound **14** (Figure 9).

### 3.6. Compound 14 induces autophagy (acridine orange staining)

**Figure 10.**
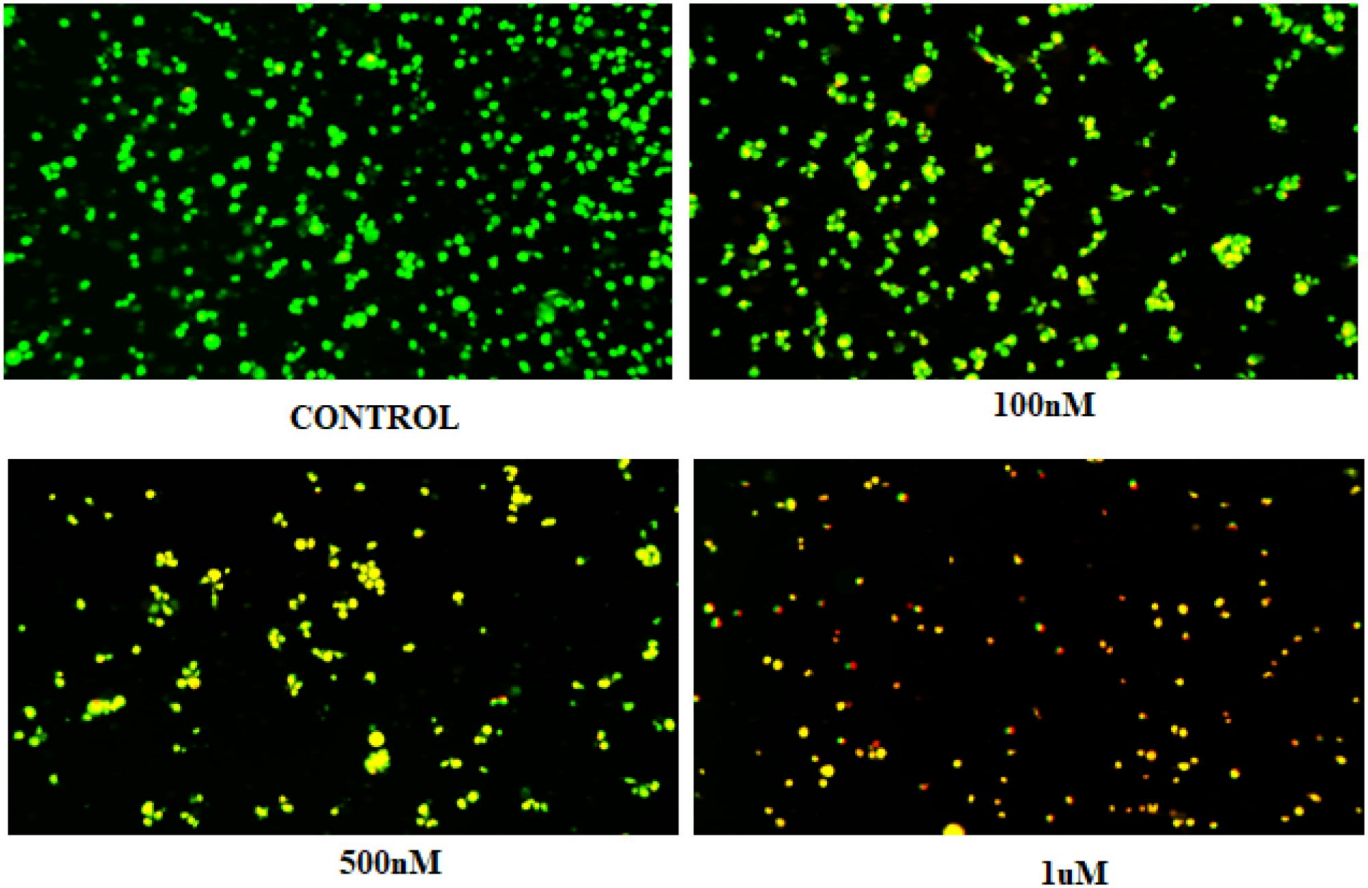
Morphology of untreated Ishikawa cells (control) and those treated with 0.1, 0.5, and 1 µM of compound **14**.

Compound **14** was evaluated for its ability to induce autophagy by acridine orange staining assay. Ishikawa cells were observed in fluorescent micrographs after staining with acridine orange (AO) and propidium iodide (PI). The results depicted in Figure 10 revealed that the cells exhibited green fluorescence in untreated conditions, however, an increase in red fluorescence was observed when cells were treated with varying concentrations of compound **14.** The appearance of red fluorescence suggesting the presence of acidic compartments, such as autophagosomes and lysosomes was attributed to the induction of autophagy. This shift towards red fluorescence represents an elevation in autophagic flux, reflecting an active process of cellular degradation and recycling. Overall, the results indicate that compound **14** induces autophagy by increasing autophagic flux in a concentration-dependent manner.

### 3.7. Compound 14 triggers concentration-dependent cleavage of LC-3

**Figure 11.**
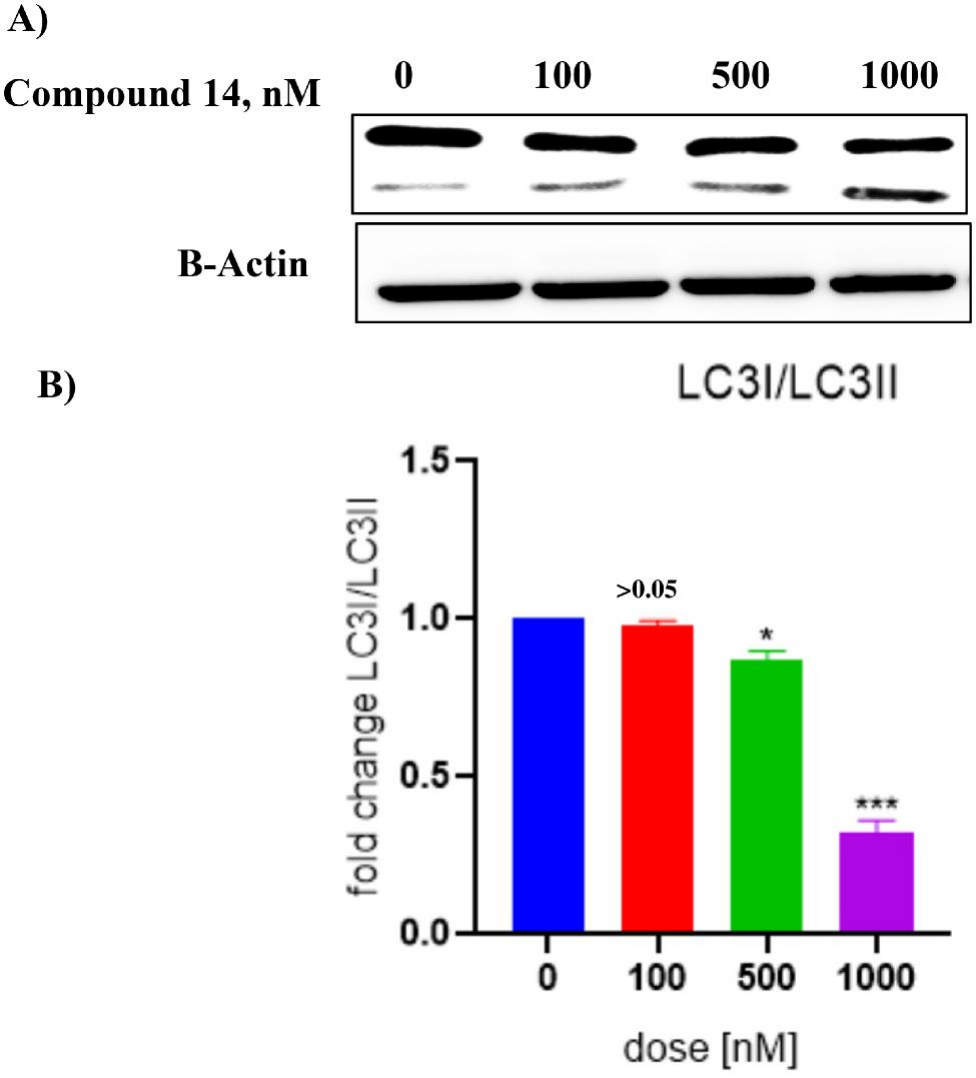
(A) Cleavage of LC-3 (autophagy marker) (B) Protein expression was measured by western blotting after 24 h treatment. The intensity of the band indicates down regulation of proteins in the cells. Data represented as the mean of three independent experiments. Statistical significance is represented as follows: ns - non-significant, * *p* < 0.05, *** *p* < 0.001.

Literature precedents indicate that autophagy triggers the cleavage of tubulin-associated protein LC-3 yielding cytosolic LC3-I (18 kDa) and autophagosome-associated LC3-II (16 kDa).^50–58^ Thus, the ability of compound **14** was evaluated to induce the cleavage of this cytosolic protein. The results of this assay revealed that compound **14** exerted concentration-dependent cleavage of LC3 in Ishikawa cells (Figure 11). Considering the aforementioned cleavage of cytosolic microtubule-associated protein light chain-3 (LC3)-I into LC3-II as an autophagy-specific marker, it was deduced that **14** is an autophagy inducer. Notably, the outcome of this assay strengthens the results of the acridine orange staining assay, ascertaining the autophagy-inducing ability of compound **14.**

### 3.8. Compound 14 dissipates the mitochondrial membrane potential (rhodamine 123 staining)

**Figure 12.**
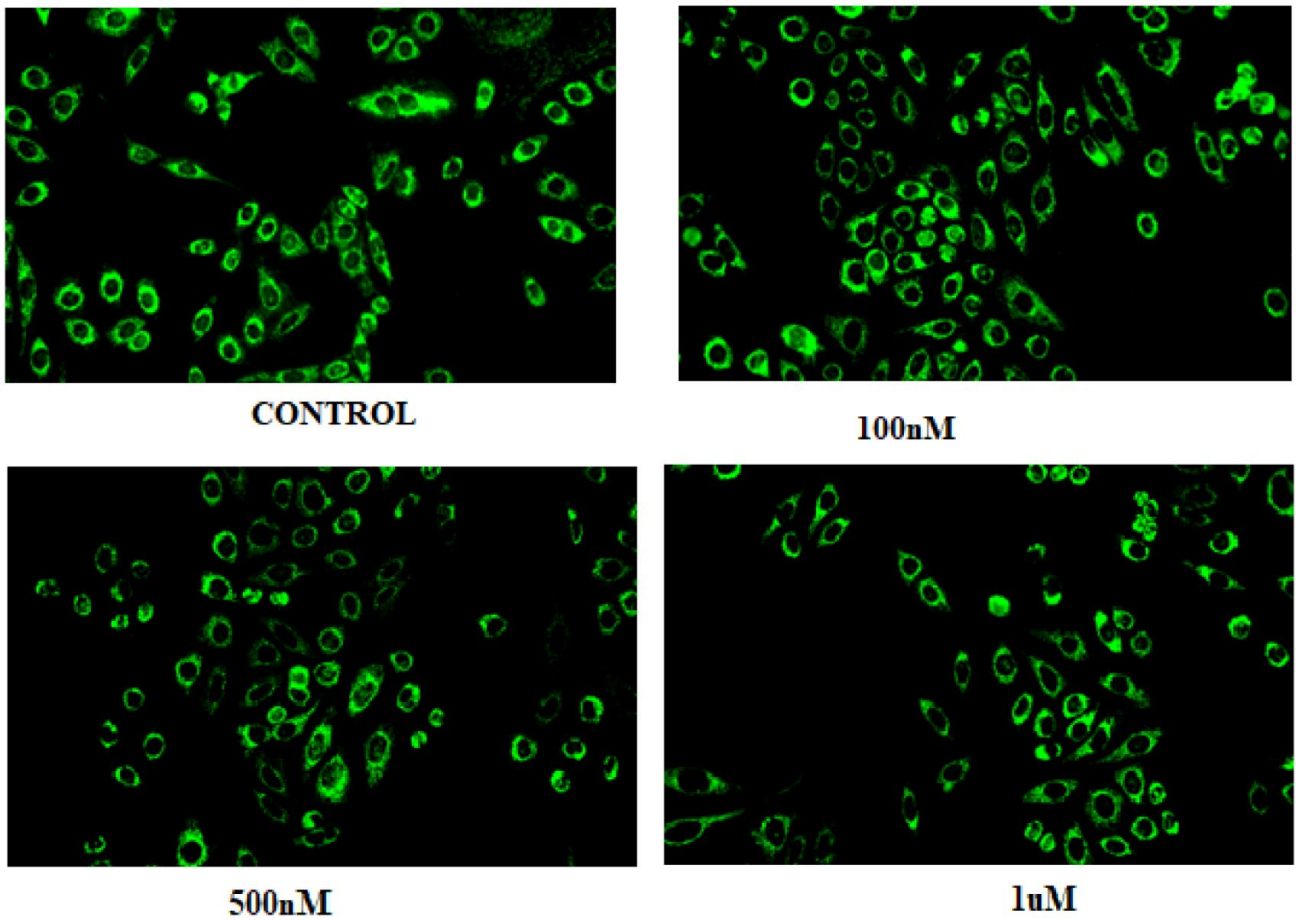
Results of Rhodamine staining assay.

Mitochondrial membrane potential was measured by rhodamine-123 (RH-123) staining. Ishikawa cells were treated at 0.1, 0.5, and 1 μM of compound **14**. Rhodamine dyes accumulate within active and polarised mitochondria under physiological conditions, resulting in robust fluorescence signals. A decline in rhodamine fluorescence intensity is observed when the dyes fail to accumulate within the mitochondria. This happens when the mitochondrial transmembrane potential is compromised. As evident from the results illustrated in Figure 12, the treatment of the Ishikawa cells with compound **14** led to a reduction in rhodamine fluorescence in a concentration-dependent manner. The decreased fluorescence patterns associated with apoptotic cell death indicate progressive dissipation of the mitochondrial transmembrane potential (Figure 12).

### 3.9. Compound 14 enhances the intracellular ROS levels

**Figure 13.**
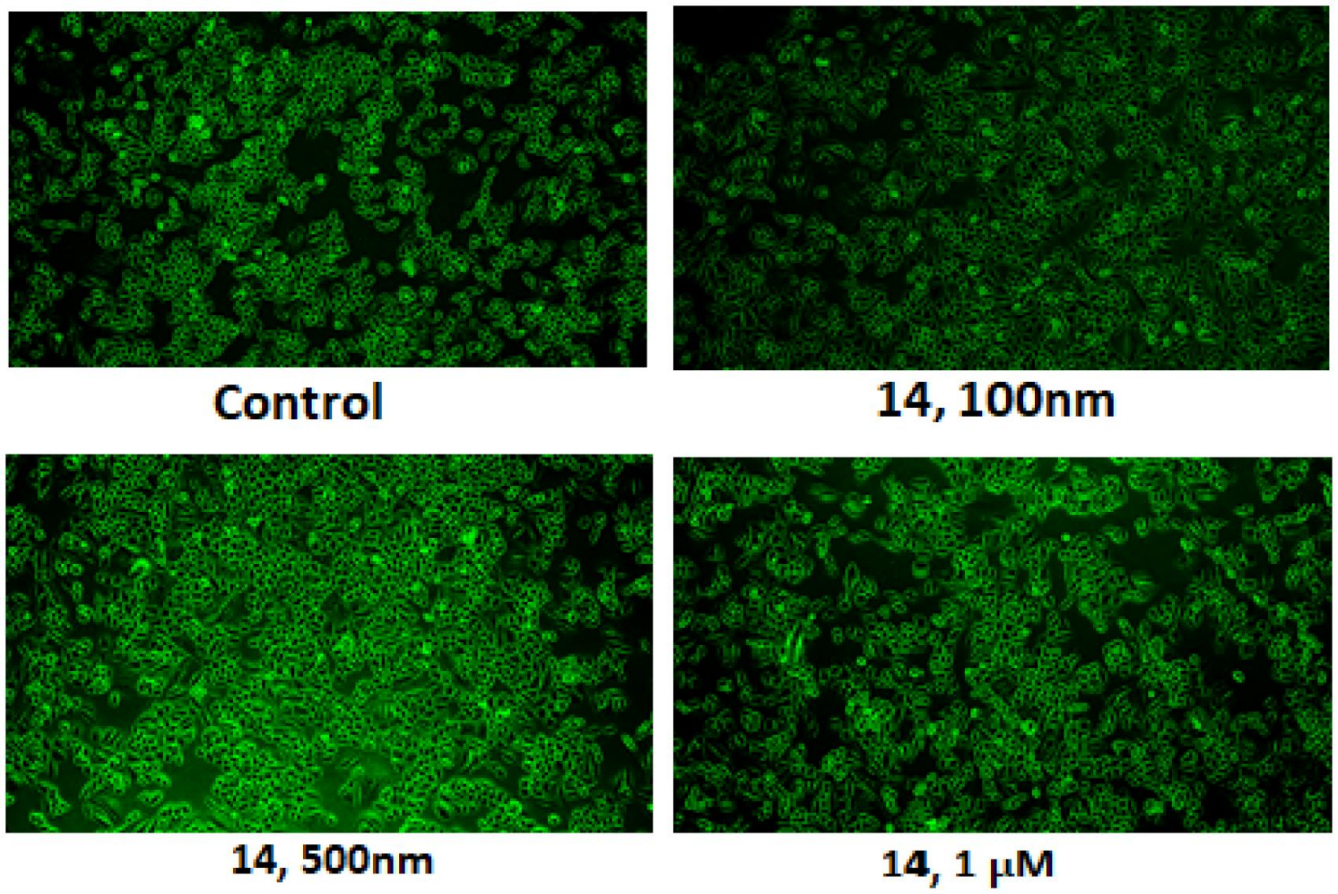
Results of DCFDA staining.

The intracellular ROS levels in control and compound **14**-treated cells were examined using the DCFDA fluorescent dye. As compared to the untreated control, a significant increase in fluorescence intensity was observed when Ishikawa cells were treated with compound **14**. The outcome of the assay reveals the potential of compound **14** to enhance the intracellular ROS levels that can cause oxidative stress in cells making the tumour cancer cells vulnerable to apoptosis (Figure 13).

### 3.10. Compound 14 triggers cell cycle arrest at the G2/M phase

**Figure 14.**
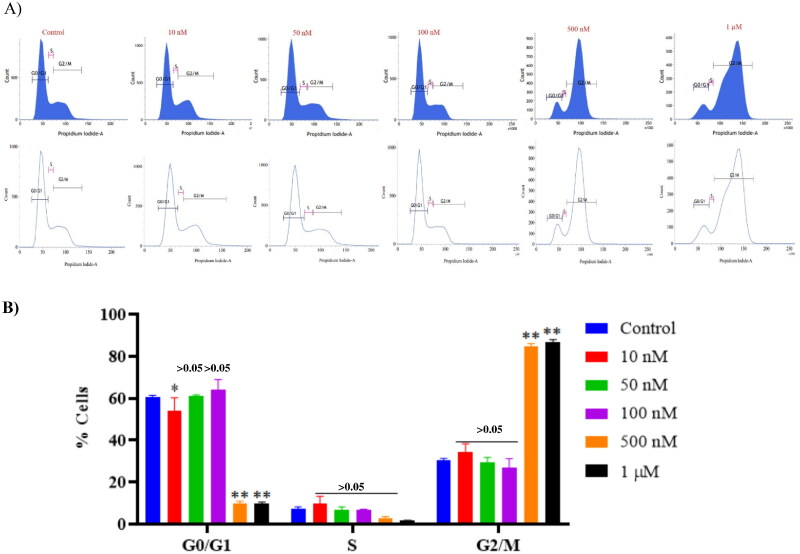
Effect of compound **14** on cell cycle proteins regulating G2/M progression at indicated concentrations. Statistical significance is represented as follows: ns - non-significant, *p < 0.05, ** *p* < 0.01.

Furthermore, the regulatory effect of compound **14** in the cell cycle distribution of Ishikawa cancer cells was evaluated via flow cytometric analysis. Ishikawa cells seeded on a 6-well plate (3 × 10^5^ cells/well) were treated with compound **14** for 48 h. After 48 h treatment of Ishikawa cells with compound **14,** the cell cycle started to be arrested at the G2/M phase. Notably, a significant increase in G2/M phase cell cycle arrest was observed with 0.5 µM and 1 µM of compound **14** as compared to the control group (Figure 14).

### 3.11. Compound 14 exerts downregulation of CDK1 protein

**Figure 15.**
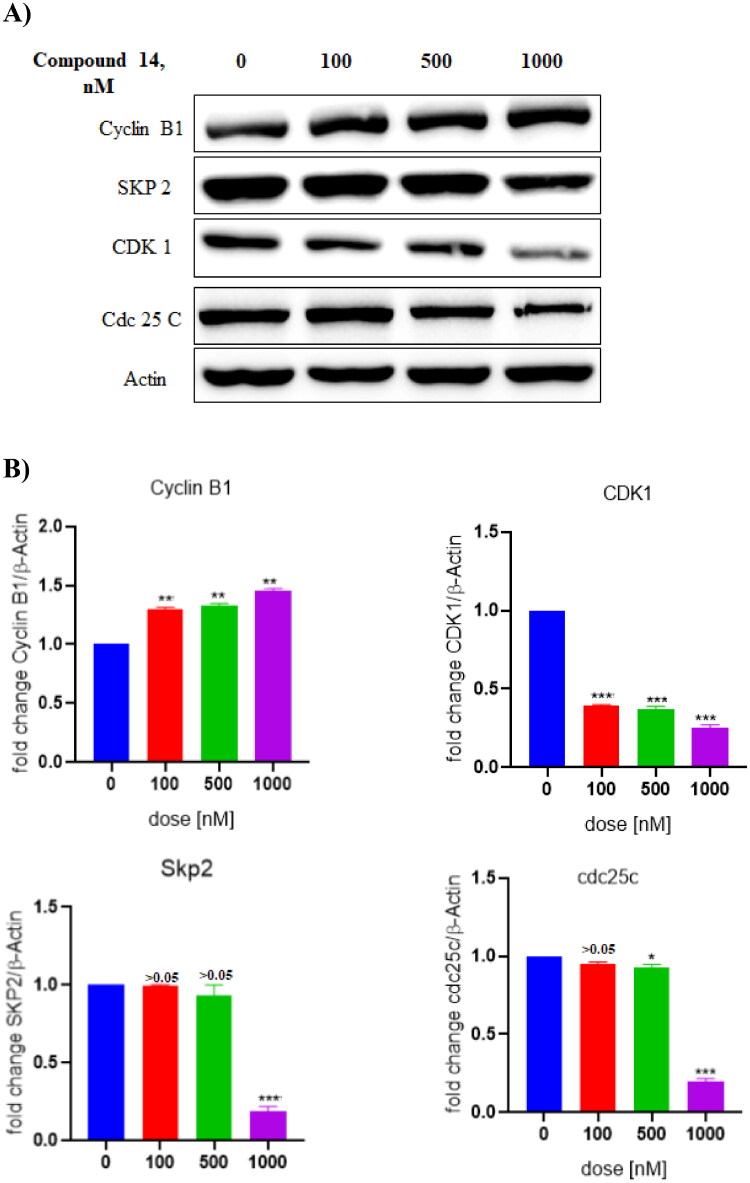
(A) Expression levels of proteins (B) Protein expression was measured by western blotting after 24 h treatment. The intensity of the band indicates the down-regulation of proteins in the cells. Data represented as the mean of three independent experiments. Statistical significance is represented as follows: ns - non-significant, *p < 0.05, ** *p* < 0.01, *** *p* < 0.001.

Western blot analysis was performed to assess the involvement of proteins in the cell cycle arrest effects of compound **14** on Ishikawa cells. As evident from the results depicted in Figure 15, the treatment of Ishikawa cells with compound **14** downregulated the expression of CDK1 protein. These results suggested that compound **14** triggered G2/M phase cell cycle arrest by inhibiting the expression of CDK1.

### 3.12. Docking study

**Figure 16.**
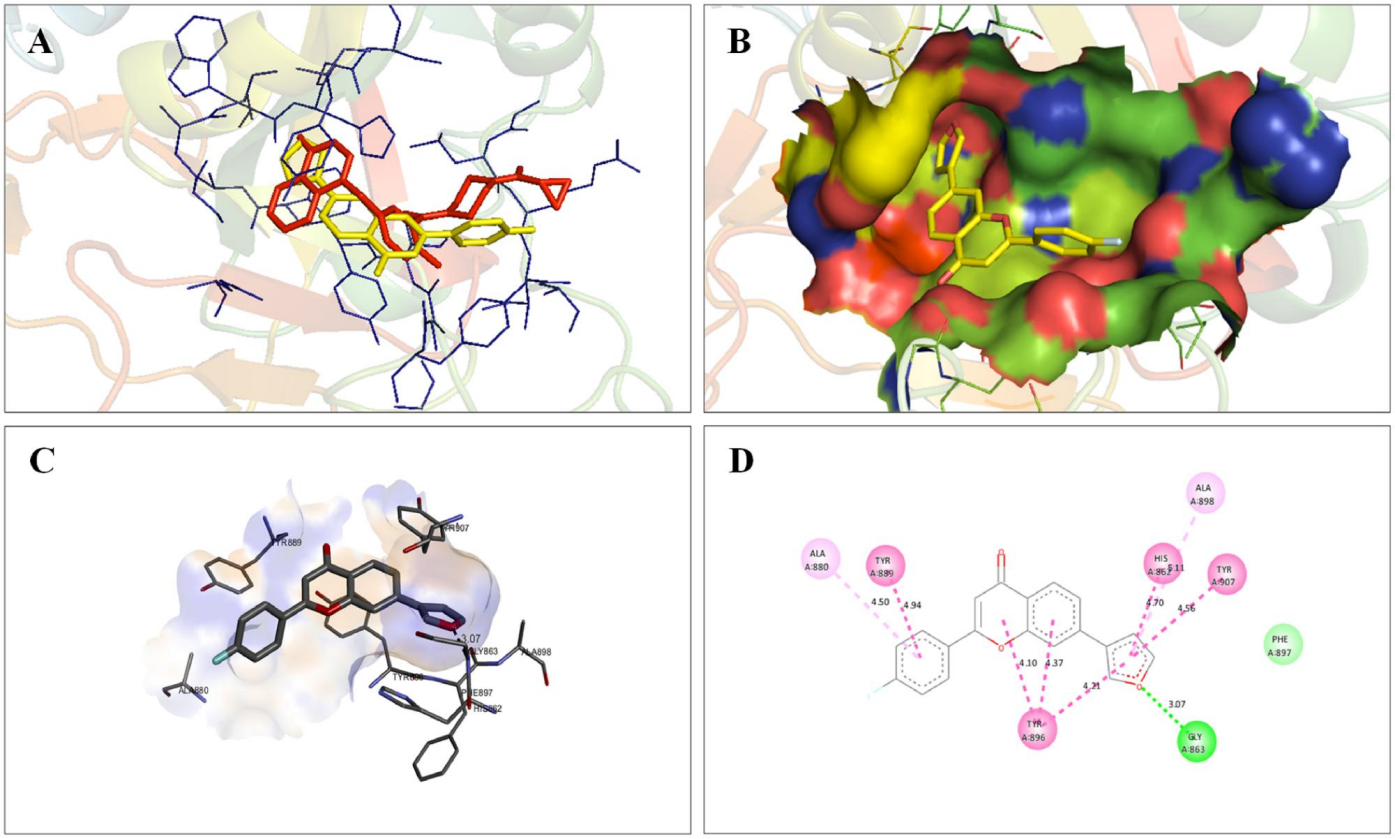
Interaction analysis of **14** with PARP1. (A) Overlay of **14** (yellow) with co-crystallized ligand (red). (B) Orientation of **14** in the active site of PARP1 protein. (C) 3D docked pose of **14** showing hydrogen bond interaction. (D) 2D docked pose of **14** showing hydrogen bond and hydrophobic interactions in the active site of PARP1 protein.

**Figure 17.**
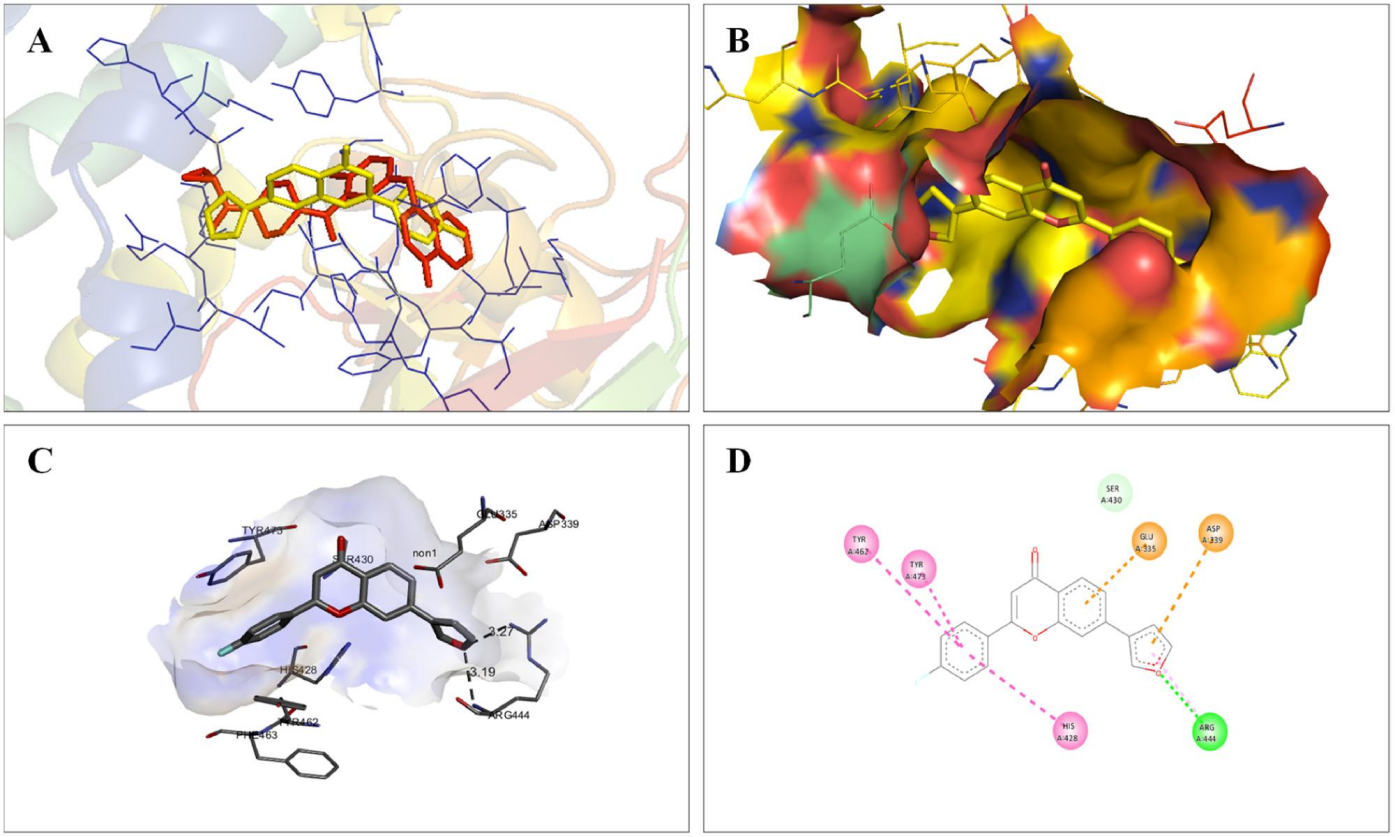
Interaction analysis of **14** with PARP2. (A) Overlay of **14** (yellow) with co-crystallized ligand (pink). (B) Orientation of **14** in the active site PARP2 protein. (C) 3D docked pose of **14** showing hydrogen bond interaction. (D) 2D docked pose of **14** showing hydrogen bond and hydrophobic interactions in the active site of PARP2 protein.

**Figure 18.**
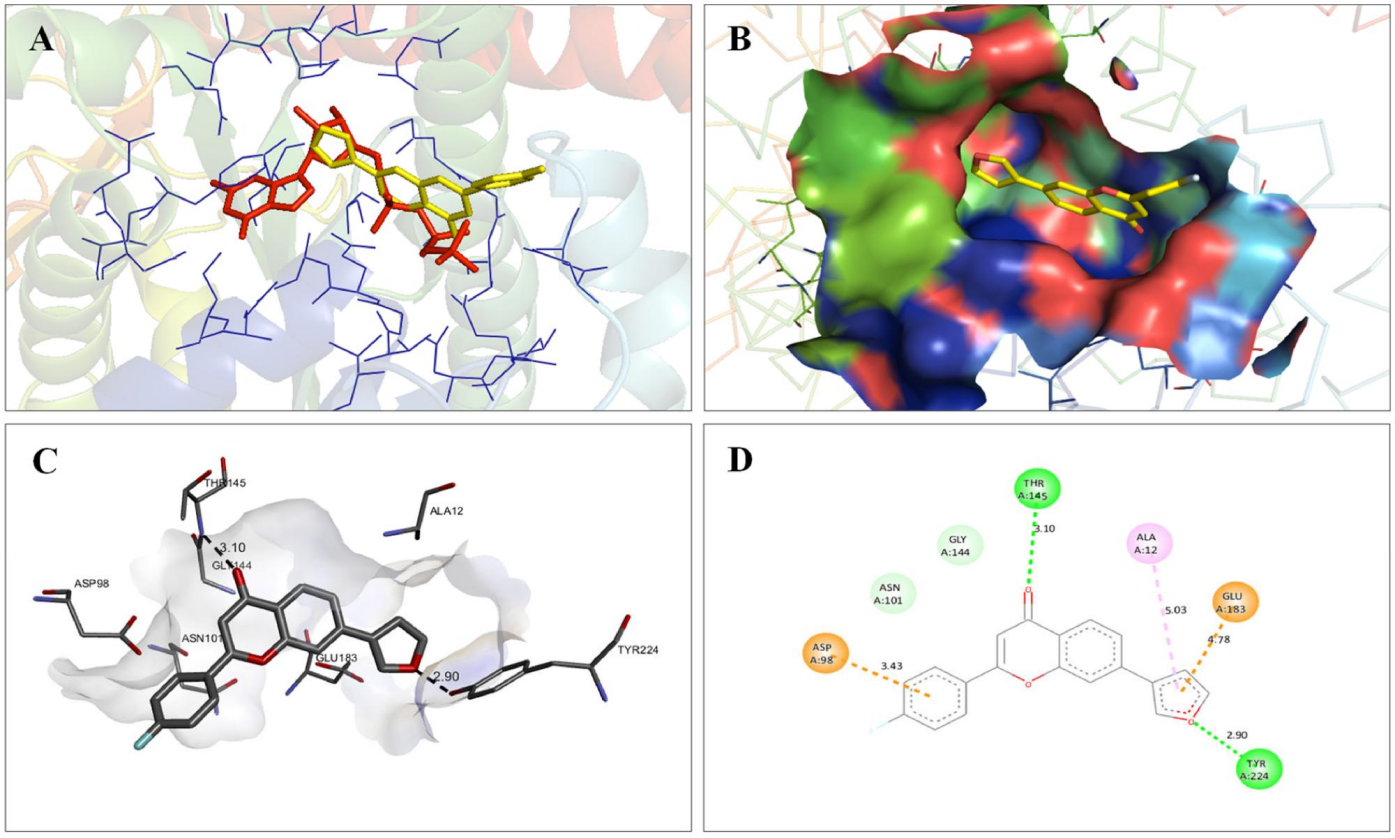
Interaction analysis of **14** with tubulin. (A) Overlay of **14** (yellow) with co-crystallized ligand (pink). (B) Orientation of **14** in the active site of tubulin protein. (C) 3D docked pose of **14** showing hydrogen bond interactions. (D) 2D docked pose of **14** showing hydrogen bond and hydrophobic interactions in the active site of tubulin protein.

Molecular modelling studies were performed to rationalise the experimental results and figure out the key interactions of compound **14** with the active site residues of PARP1, PARP2, and tubulin. The docking score and residues involved in binding interactions of test compounds with PARP1, PARP2 and tubulin are presented in Table 5. The interactions of **14** with active site residues of PARP1 (PDB ID: 5DS3) are outlined in Figure 16. From the docking study, several key interactions were figured. Notably, a hydrogen bond interaction between the “–O–” functionality of this compound and the “–NH–” group of Gly863 residue (bond distance: 3.07 Å) was observed. Also, compound **14** was involved in some hydrophobic interactions with His862, Tyr889, Tyr896 & Tyr907 residues (pi-pi type) and Ala880 & Ala898 residues (pi-alkyl type) in the active site of PARP1. The docking results of compound **14** with active site residues of PARP2 (PDB ID: 4TVJ) are shown in Figure 17. Two hydrogen bond interactions were between the “–O–” functionality of this compound and the “–NH–” groups of Arg444 residue (bond distance: 3.19 Å and 3.27 Å). Also, hydrophobic interactions with His428, Tyr462 & Tyr473 residues (pi-pi type), Arg444 residue (pi-alkyl type) and electrostatic interactions with Glu335 & Asp339 residues (pi-charge type) in the active site of PARP2 were seen. Flavone **14** was also docked within the active site of tubulin (PDB ID: 1SA0) (Figure 18). Two hydrogen bond interactions between the “C = O” & “–O–” functionality of this compound and “–NH–” and phenolic “–OH–” groups of Thr145 & Tyr224 residues in the active site of tubulin (bond distance: 3.10 Å and 2.90 Å, respectively) were observed. Compound **14** also exhibited hydrophobic interactions with Ala12 residue (pi-alkyl type) and electrostatic interactions with Asp98 & Glu183 residues (pi-charge type) in the active site of tubulin protein. Taken together, the favourable binding pattern of flavone **14** within the active site of PARP1, PARP2 and tubulin suggests its prevailing role in dual PARP-tubulin inhibitory activity.

**Table 5.** Docking score and residues involved in binding interactions of compound **14** with PARP1, PARP2 and tubulin.

Compound	Docking score (kcal/mol)	Residues involved in hydrogen bonds (bond distance)	Residues involved in hydrophobic & other interactions
PARP1 (PDB ID: 5DS3)			
**14**	−9.4	Gly863 (3.07 Å)	His862, Ala880, Tyr889, Tyr896, Ala898, Tyr907
PARP2 (PDB ID: 4TVJ)			
**14**	−9.9	Arg444 (3.19 Å & 3.27 Å)	Glu335, Asp339, His428, Arg444, Tyr462, Tyr473
Tubulin (PDB ID: 1SA0)			
**14**	−8.0	Thr145 (3.10 Å), Tyr224 (2.90 Å)	Ala12, Asp98, Glu183

**Figure 19.**
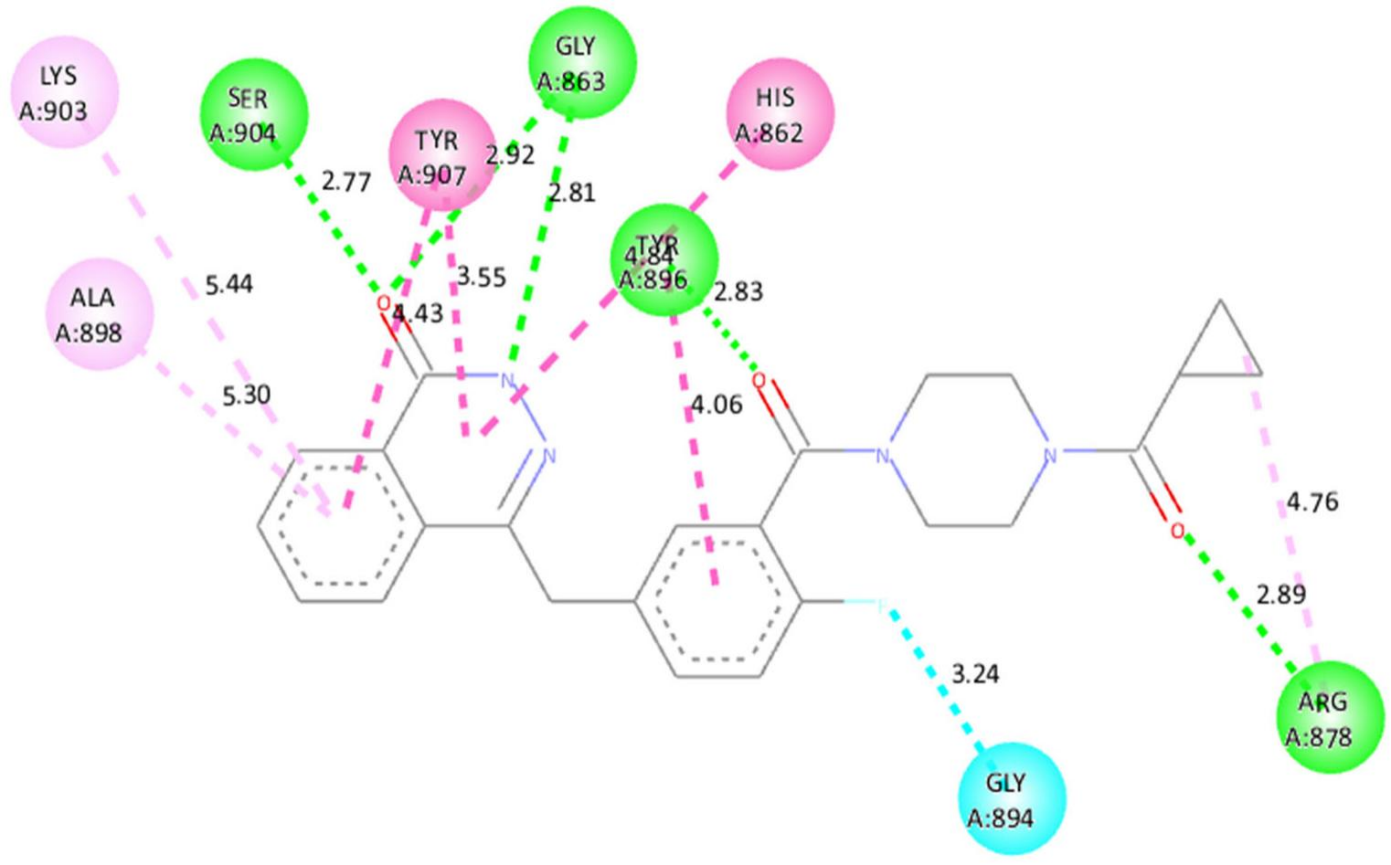
Docked pose showing binding interactions of olaparib with PARP1.

**Figure 20.**
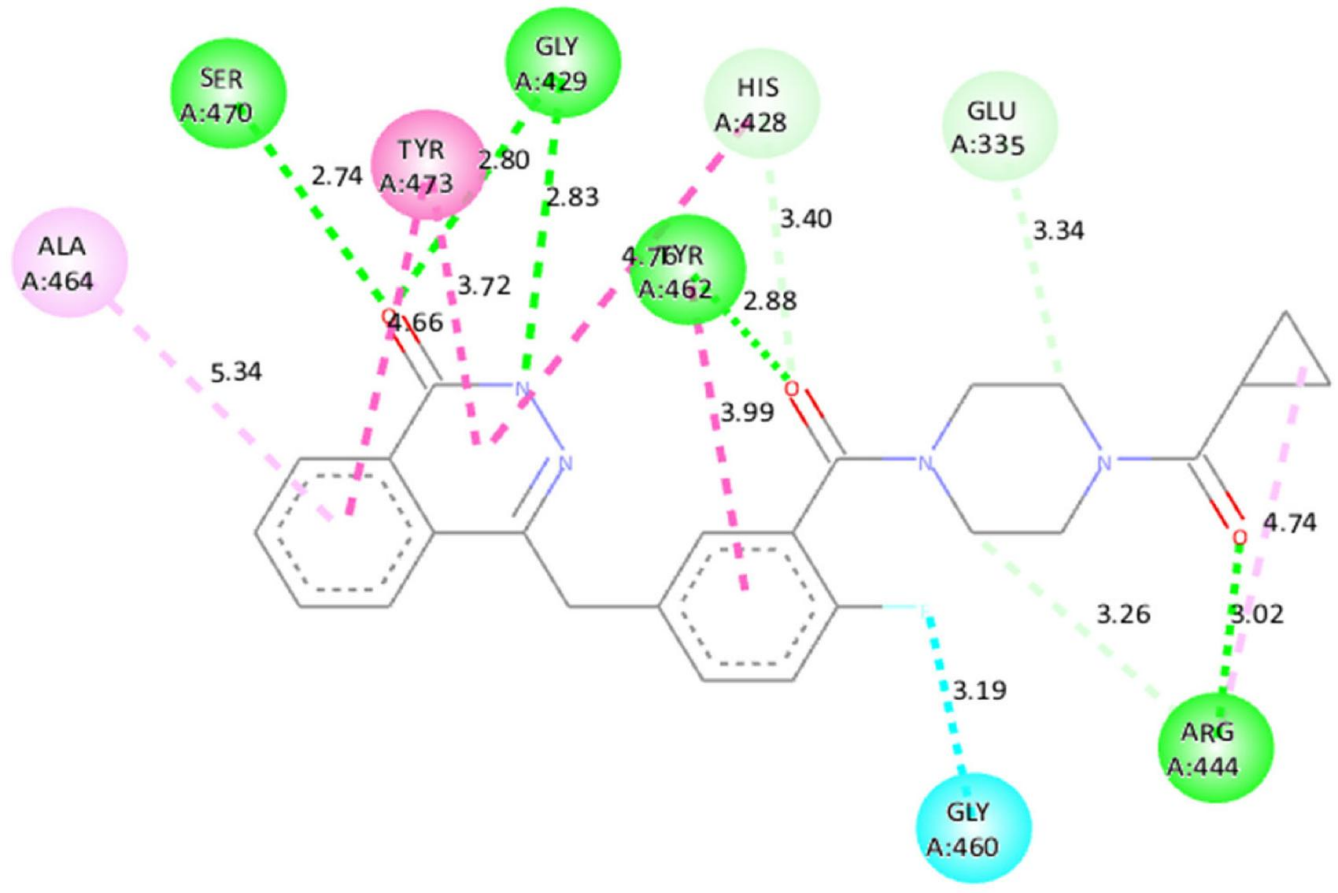
Docked pose showing binding interactions of olaparib with PARP2.

**Figure 21.**
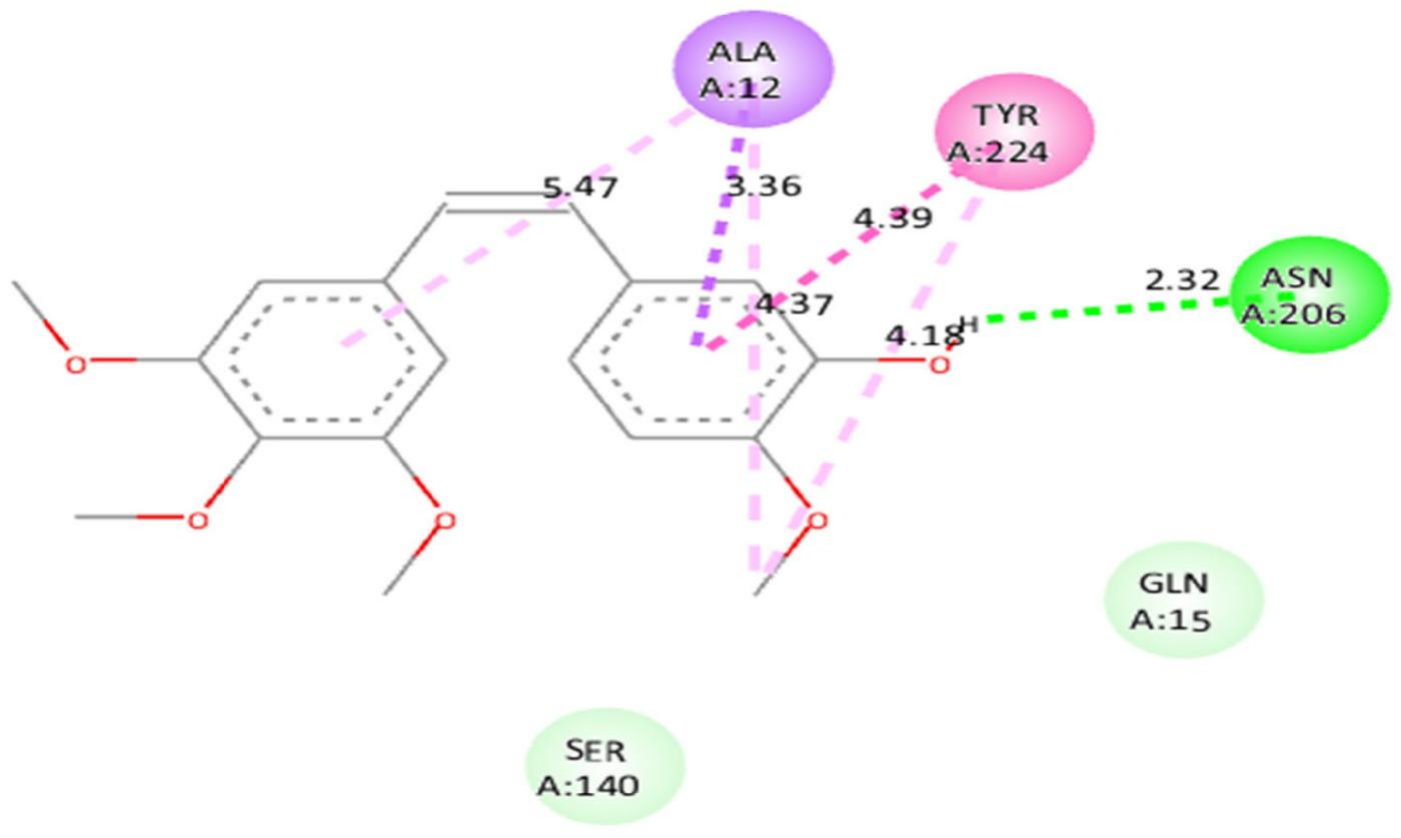
Docked pose showing binding interactions of combretastatin A4 with tubulin.

To figure out the similarity in the binding pattern of compound **14** and the standard PARP and tubulin inhibitor, the interaction profile of Olaparib (PARP inhibitor) and Combretastatin (Tubulin inhibitor) was studied. Olaparib (co-crystallized and known ligand of PARP1 and PARP2) was docked with PARP1 and PARP2 proteins (docking score was −11.2 and −11.4 kcal/mol, respectively). Combretastatin A4 (a known inhibitor of tubulin) was docked with tubulin (docking score was −7.6 kcal/mol). Olaparib showed hydrogen bond interactions with Ser904 (bond distance: 2.77 Å), Gly863 (bond distance: 2.81 and 2.92 Å), Arg878 (bond distance: 2.89 Å) and Tyr896 (bond distance: 2.83 Å), hydrophobic interactions with Ala898, Lys903, Tyr907, Tyr896, His862 and Arg878 residues, and halogen interaction with Gly894 residue of PARP1 (Figure 19). Olaparib showed hydrogen bond interactions with Ser470 (bond distance: 2.74 Å), Gly429 (bond distance: 2.80 and 2.83 Å), Tyr462 (bond distance: 2.88 Å) and Arg444 (bond distance: 3.02 Å), hydrophobic interactions with Ala464, Tyr473, His428, Tyr462 and Arg444 residues, and halogen interaction with Gly460 residue of PARP2 (Figure 20). Combretastatin A4 showed hydrogen bond interaction with Asn206 residue (bond distance: 2.32 Å), and hydrophobic interactions with Ala12 and Tyr224 residues of tubulin (Figure 21). Careful observation of the docking results of compound **14** (most active compound), olaparib, and combretastatin revealed that hydrogen bonding as well as hydrophobic interactions with the amino acid residues of PARP1, PARP2, and Tubulin are the crucial interactions for the stabilisation of the compound within the active site.

### 3.13. ADME (absorption, distribution, metabolism and excretion) study

The results obtained from the ADME study (*in silico*) revealed that compound **14** possessed good pharmacokinetic parameters for oral bioavailability and drug-likeness (Table 6) as contrived by Lipinski’s rule of five.

**Table 6.** ADME parameters of compound **14** predicted using the SwissADME online tool.

Property	Predicted value
Molecular formula	C_19_H_11_FO_3_
Molecular weight (g/mol)	306.29
Number of heavy atoms	23
Number of H-bond acceptors	4
Number of H-bond donors	0
Number of rotatable bonds	2
Topological polar surface area (Å²)	43.35
Molar refractivity	85.58
Partition coefficient (Log P)	4.14
Log S (Estimated solubility)	−5.02
Solubility class	Moderately soluble
Bioavailability score	0.55
PGP substrate	No
Log K_p_ (skin permeation)	−5.08 cm/s
Drug-likeness (Lipinski’s rule of five)	Yes
Number of violations	0
Pan-assay interference structures (PAINS) alerts	0

## 4. Conclusion

This study conducted structural engineering of the flavone framework to construct dual PARP-tubulin inhibitors. Structural activity relationship was established that indicated that appendage of a monocyclic heteroaryl arm (**position 7, benzopyran framework**) and placement of a 4-fluorophenyl ring at **position 2** (benzopyran scaffold) resulted in the enhancement of the cell growth inhibitory effects against the Ishikawa cell lines. *In-vitro* cytotoxicity results led us to identify compound **14** as a potent cell growth inhibitor of the employed human cancer cell lines (Ishikawa cell lines). Results of mechanistic studies indicated that the cytotoxic effects of compound **14** against Ishikawa cell lines were mediated through dual modulation (inhibition) of PARP and Tubulin. Also, flavone **14** demonstrated apoptosis and autophagy-inducing ability as evident from the results of DAPI, rhodamine, acridine orange staining, and DCFDA staining. Western blot experiment also confirmed the ability of compound **14** to induce autophagy as **14** dose-dependently cleaved LC-3 (**autophagosome marker**). Compound **14** also downregulated the expression level of CDK1 protein. Flow cytometric evaluation of flavone **14** revealed that it caused cell cycle arrest at the G2/M phase. In a nutshell, these outcome of this study presents a promising dual PARP-tubulin inhibitor endowed with efficacy against endometrial cancer.

## 5. Experimental

### 5.1. Chemistry

The instruments used for the characterisation of the final compounds are as follows: Bruker DRX-300 and 600 spectrometers (^1^H and ^13^C NMR) and JEOL (JMS-700) EI mass spectrometer (HRMS). The purity of the final compounds was determined using Shimadzu LC-2030C (HPLC).

#### 5.1.1. General procedures for the synthesis of compounds 28–32

To a solution of **22** (2.0 gm, 0.009 mol, 1.0 equiv.) in ethanol (50 ml) was added corresponding substituted benzaldehydes **23–27** (0.009 mol, 1.0 equiv.), followed by the dropwise addition of 9 ml of 2.0 M sodium hydroxide aqueous solution (0.018 mol, 2.0 equiv.). The reaction mixture was stirred at room temperature for 24 h and the progress of the reaction was monitored by TLC. After completion of the reaction, the pH of the reaction mixture was neutralised using 3 N HCl. The resulting precipitates were filtered, washed with water, dried and was carried to next step without purification.

##### 5.1.1.1. (E)-1–(4-bromo-2-hydroxyphenyl)-3-(phenyl)prop-2-en-1-one (28)

Yield 94%; ^1^H NMR (300 MHz, DMSO-*d_6_*): δ 8.15 (d, J = 8.4 Hz, 1H), 7.81–8.02 (m, 4H), 7.48–7.50 (m, 4H), 7.27 (d, J = 1.8 Hz, 1H), 7.19–7.23 (m, 1H).

##### 5.1.1.2. (E)-1–(4-bromo-2-hydroxyphenyl)-3–(4-methoxyphenyl)prop-2-en-1-one (29)

Yield 70%; ^1^H NMR (300 MHz, CD_3_OD): δ 8.07 (d, J = 8.5 Hz, 1H), 7.95 (d, J = 15.5 Hz, 1H), 7.77–7.810 (m, 3H), 7.20 (d, J = 1.9 Hz, 1H), 7.17 (dd, J = 8.6 and 2.0 Hz, 1H), 7.02–7.05 (m, 2H), 3.90 (s, 3H).

##### 5.1.1.3. (E)-1–(4-bromo-2-hydroxyphenyl)-3–(4-fluorophenyl)prop-2-en-1-one (30)

Yield 73%; ^1^H NMR (300 MHz, CDCl_3_) δ 12.95 (s, 1H), 7.94 (d, J = 15.5 Hz, 1H), 7.79 (d, J = 8.6 Hz, 1H), 7.70 (dd, J = 8.7 and 5.4 Hz, 2H), 7.53 (d, J = 15.5 Hz, 1H), 7.27 (d, J = 1.9 Hz, 1H), 7.17 (s, 1H), 7.14 (s, 1H), 7.10–7.13 (m, 1H).

##### 5.1.1.4. (E)-1–(4-bromo-2-hydroxyphenyl)-3–(4-nitrophenyl)prop-2-en-1-one (31)

Yield 75%; ^1^H NMR (300 MHz, DMSO-*d_6_*): δ 8.29–8.32 (m, 2H), 8.14–8.17 (m, 2H), 8.12 (d, J = 4.5 Hz, 1H), 8.09 (d, J = 2.6 Hz, 1H), 7.88 (d, J = 15.7 Hz, 1H), 7.28 (d, J = 2.0 Hz, 1H), 7.23 (dd, J = 8.5 and 2.0 Hz, 1H).

##### 5.1.1.5. (E)-1–(4-bromo-2-hydroxyphenyl)-3–(3,4,5-trimethoxyphenyl)prop-2-en-1-one (32)

Yield 92%; ^1^H NMR (300 MHz, CDCl_3_): δ 13.01 (S, 1H), 7.89 (d, J = 15.6 Hz, 1H), 7.81 (d, J = 18.7 Hz, 1H), 7.47 (d, J = 15.3 Hz, 1H), 7.26 (d, J = 2.1 Hz, 1H), 7.12 (dd, J = 8.4 and 2.1 Hz, 1H), 6.91 (s, 2H), 3.97 (s, 6H), 3.95 (s, 3H).

#### 5.1.2. General procedures for the synthesis of compounds 33–37

To a solution of corresponding substituted chalcone intermediates **28–32** (0.006 mol, 1.0 equiv) in DMSO (20 ml) was added iodine pellets (0.0006 mol, 0.1 equiv.), and the reaction mixture was refluxed for 24 h and progress of the reaction was monitored by TLC. After completion of the reaction, sodium thiosulphate solution (50 ml) was added in the reaction mixture and the compound was precipitated. The resulting precipitates were filtered, washed with water, dried and was carried to next step without purification.

##### 5.1.2.1. *7-bromo-2-phenyl-4H-chromen-4-one* (*33*)

Yield 90%; ^1^H NMR (300 MHz, CDCl_3_): δ 8.13 (d, J = 8.5 Hz, 1H), 7.93–7.96 (m, 2H), 7.83 (d, J = 1.8 Hz, 1H), 7.55–7.61 (m, 4H), 6.86 (s, 1H).

##### 5.1.2.2. 7-bromo-2–(4-methoxyphenyl)-4H-chromen-4-one (34)

Yield 81%; ^1^H NMR (300 MHz, CDCl_3_): δ 8.13 (d, J = 8.3 Hz, 1H), 7.92 (s, 1H), 7.89 (s, 1H), 7.82 (s, 1H), 7.58 (d, J = 8.4 Hz, 1H), 7.07 (d, J = 8.7 Hz, 2H), 6.86 (s, 1H), 3.94 (s, 3H).

##### 5.1.2.3. *7-bromo-2–(4-fluorophenyl)-4H-chromen-4-one* (*35*)

Yield 78%; ^1^H NMR (300 MHz, CDCl_3_): δ 8.12 (d, J = 8.4 Hz, 1H), 7.92–7.96 (m, 2H), 7.81 (d, J = 1.8 Hz, 1H), 7.59 (dd, J = 8.4 and 1.8 Hz, 1H), 7.23–7.29 (m, 2H), 6.79 (s, 1H).

##### 5.1.2.4. *7-bromo-2–(4-nitrophenyl)-4H-chromen-4-one* (*36)*

Yield 72%; ^1^H NMR (300 MHz, DMSO-*d_6_*): 8.18–8.25 (m, 3H), 7.98 (d, J = 8.4 Hz, 1H), 7.72 (d, J = 1.8 Hz, 1H), 7.46 (t, J = 9 Hz, 2H), 7.11 (s, 1H).

##### 7-bromo-2–(3,4,5-trimethoxyphenyl)-4H-chromen-4-one (37)

Yield 80%; %. ^1^H NMR (300 MHz, CDCl_3_): δ 8.12 (d, J = 8.6 Hz, 1H), 7.84 (d, J = 1.7 Hz, 1H), 7.59 (dd, J = 8.5 and 1.7 Hz, 1H), 7.14 (s, 2H), 6.80 (s, 1H), 4.00 (s, 6H), 3.97 (s, 3H).

#### 5.1.3. General procedures for the synthesis of compounds 1–21

To a solution of corresponding cyclic bromo flavone intermediates **33–37** (0.0014 mol, 1.0 equiv.) in dioxane (18 ml), was added corresponding aryl boronic acid (0.0015 mol, 1.1 equiv.), tetrakis (triphenylphosphine)palladium(0) (0.00014 mol, 0.1 equiv.) and sodium carbonate (0.0042 mol, 3 equiv.), and the reaction mixture was refluxed for 2 h. After completion of the reaction (TLC), the reaction mixture was passed through celite. To the filtrate, water (100 ml) was added and the extraction was done with ethyl acetate (50 ml × 3). The combined organic layer was dried over anhydrous magnesium sulphate and concentrated under reduced pressure and the residue was further purified by performing silica gel column chromatography (ethyl acetate: n-hexane: 1:5 to 1:1) to give compounds **1–21**.

##### 5.1.3.1. 2,7-diphenyl-4H-chromen-4-one (1)

Percentage yield: 36%; HPLC purity: 99.31%; mp: 153–157 °C; ^1^H NMR (300 MHz, DMSO-*d_6_*) δ 8.13–8.20 (m, 4H), 7.85–7.91 (m, 3H), 7.48–7.66 (m, 6H), 7.10 (s, 1H). ^13^C NMR (600 MHz, DMSO-*d_6_*): δ 177.45, 163.27, 156.61, 146.32, 138.48, 132.34, 131.52, 129.66, 129.59, 129.35, 127.64, 126.83, 125.90, 124.51, 122.58, 116.51, 107.44. HRMS (ESI) for C_21_H_15_O_2_ [M + H^+^]: calcd; 299.1072 found, 299.1141.

###### 5.1.3.2. 7-(Furan-3-yl)-2-phenyl-4H-chromen-4-one (2)

Percentage yield: 39%; HPLC purity: 98.89%. mp: 163–167 °C; ^1^H NMR (600 MHz, DMSO-*d_6_*): δ 8.41 (s, 1H), 8.08 (d, J = 6.6 Hz, 2H), 7.99–8.01 (m, 2H), 7.79 (s, 1H), 7.74 (d, J = 7.8 Hz, 1H), 7.55–7.60 (m, 3H), 7.12 (s, 1H), 6.97 (s, 1H). ^13^C NMR (600 MHz, DMSO-*d_6_*): δ 177.27, 163.11, 156.79, 145.36, 141.95, 138.53, 132.31, 131.57, 129.59, 126.77, 125.81, 125.05, 123.44, 122.20, 114.89, 109.13, 107.45. HRMS (ESI) for C_19_H_13_O_3_ [M + H^+^]: calcd; 289.0865 found, 289.1003.

###### 5.1.3.3. 2-phenyl-7-(thiophen-2-yl)-4H-chromen-4-one (3)

Percentage yield: 28%; HPLC purity: 98.27%; mp: 186–189 °C; ^1^H NMR (300 MHz, DMSO-*d_6_*): δ 8.16–8.19 (m, 2H), 8.12 (d, J = 1.8 Hz, 1H), 8.08 (d, J = 8.4 Hz, 1H), 7.84–7.86 (m, 2H), 7.76 (dd, J = 5.1 and 1.2 Hz, 1H), 7.61–7.66 (m, 3H), 7.25–7.28 (m, 1H), 7.07 (s, 1H). ^13^C NMR (600 MHz, DMSO-*d_6_*): δ 177.16, 163.22, 156.69, 141.57, 139.73, 132.35, 131.46, 129.58, 129.46, 128.65, 126.84, 126.24, 123.18, 122.45, 114.53, 107.46. HRMS (ESI) for C_19_H_13_O_2_S [M + H^+^]: calcd; 305.0636 found, 305.0672.

###### 5.1.3.4. 7–(2,3-dihydrobenzo[b][1,4]dioxin-6-yl)-2-phenyl-4H-chromen-4-one (4)

Percentage yield: 39%; HPLC purity: 97.00%; mp: 181–184 °C; ^1^H NMR (300 MHz, DMSO-*d_6_*): δ 8.16–8.19 (m, 2H), 8.06–8.09 (m, 2H), 7.79 (dd, J = 8.4 and 1.8 Hz, 1H), 7.60–7.65 (m, 3H), 7.41 (d, J = 2.1 Hz, 1H), 7.36–7.39 (m, 1H), 7.07 (s, 1H), 7.03 (d, J = 8.4 Hz, 1H), 4.33 (s, 4H). ^13^C NMR (600 MHz, DMSO-*d_6_*): δ 177.43, 163.17, 156.66, 145.72, 144.78, 144.30, 132.32, 131.59, 131.55, 129.58, 126.82, 125.76, 124.06, 122.15, 120.63, 118.20, 116.14, 115.76, 107.37, 64.72, 64.56. HRMS (ESI) for C_23_H_17_O_4_ [M + H^+^]: calcd; 357.1127 found, 357.1059.

###### 5.1.3.5. 2-phenyl-7-(quinolin-6-yl)-4H-chromen-4-one (5)

Percentage yield: 20%; HPLC purity: 95.85%; mp: 136–140 °C; ^1^H NMR (600 MHz, DMSO-*d_6_*): δ 8.15 (d, J = 7.2 Hz, 2H), 8.06–8.09 (m, 3H), 7.86 (d, J = 7.8 Hz, 1H), 7.59–7.61 (m, 5H), 7.54 (d, J = 8.4 Hz, 1H), 7.42 (s,1H), 7.02 (s, 1H), 6.55 (s, 1H). ^13^C NMR (600 MHz, DMSO-*d_6_*): δ 177.44, 163.34, 156.65, 151.75, 147.96, 145.37, 137.15, 136.31, 133.23, 132.55, 131.94, 131.87, 131.51, 130.17, 129.61, 129.26, 129.18, 128.95, 127.23, 126.85, 126.05, 124.78, 122.61, 117.05, 107.52. HRMS (ESI) for C_24_H_16_NO_2_ [M + H^+^]: calcd; 350.1181 found, 350.1187.

###### 5.1.3.6. 7-(1H-indol-5-yl)-2-phenyl-4H-chromen-4-one (6)

Percentage yield: 35%; HPLC purity: 97.41%; mp: 246–249 °C; ^1^H NMR (600 MHz, DMSO-*d_6_*): δ 11.23 (s, 1H), 8.13 (d, J = 7.2 Hz, 2H), 8.07 (s, 1H), 8.04 (d, J = 10.2 Hz, 1H), 7.84 (d, J = 8.4 Hz, 1H), 7.56–7.58 (m, 5H), 7.52 (d, J = 4.2 Hz, 1H), 7.39 (s, 1H), 6.99 (s, 1H), 6.52 (s, 1H). ^13^C NMR (600 MHz, DMSO-*d_6_*): δ 177.53, 163.11, 156.77, 148.24, 136.70, 132.28, 131.94, 131.65, 129.60, 129.47, 128.78, 127.03, 126.82, 125.68, 124.54, 121.66, 120.94, 119.58, 115.78, 112.59, 107.36, 102.39. HRMS (ESI) for C_23_H_16_NO_2_ [M + H^+^]: calcd; 338.1181 found, 338.3465.

###### 5.1.3.7. 2–(4-methoxyphenyl)-7-phenyl-4H-chromen-4-one (7)

Percentage yield: 29%; HPLC purity: 96.04%; mp: 172–175 °C; ^1^H NMR (300 MHz, CDCl_3_): δ 8.32 (dd, J = 8.2 and 0.4 Hz, 1H), 7.93–7.98 (m, 2H), 7.81 (dd, J = 1.5 and 0.3 Hz, 1H), 7.67–7.75 (m, 3H), 7.46–7.57 (m, 3H), 7.05–7.10 (m, 2H), 6.81 (s, 1H), 3.94 (s, 3H). ^13^C NMR (600 MHz, DMSO-*d_6_*): δ 177.29, 163.36, 162.66, 156.54, 146.14, 138.55, 129.66, 129.31, 128.74, 127.64, 125.86, 124.38, 123.64, 122.57, 116.43, 115.04, 105.94, 55.99. HRMS (ESI) for C_22_H_17_O_3_ [M + H^+^]: calcd; 329.1178 found, 329.1207.

###### 5.1.3.8. 7-(Furan-3-yl)-2–(4-methoxyphenyl)-4H-chromen-4-one (8)

Percentage yield: 19%; HPLC purity: 98.43%; mp: 173–176 °C; ^1^H NMR (300 MHz, CD_3_OD): δ 8.21–8.22 (m, 1H), 8.16 (d, J = 8.3 Hz, 1H), 8.05–8.10 (m, 2H), 7.94 (d, J = 1.6 Hz, 1H), 7.75 (dd, J = 8.3 and 1.5 Hz, 1H), 7.69 (t, J = 1.8 Hz, 1H), 7.13–7.18 (m, 2H), 7.03 (dd, J = 2.0 and 0.9 Hz, 1H), 6.86 (s, 1H), 3.94 (s, 3H). ^13^C NMR (600 MHz, CDCl_3_): δ 177.97, 163.39, 162.40, 156.67, 144.31, 140.00, 138.09, 127.96, 126.18, 125.24, 124.01, 122.85, 122.48, 114.46, 114.28, 108.60, 106.28, 55.48. HRMS (ESI) for C_20_H_15_O_4_ [M + H^+^]: calcd; 319.0970 found, 319.1064.

###### 5.1.3.9. 2–(4-methoxyphenyl)-7-(thiophen-2-yl)-4H-chromen-4-one (9)

Percentage yield: 26%; HPLC purity − 97.78%; mp: 180–184 °C; ^1^H NMR (300 MHz, CD_3_OD): δ 7.30 (d, J = 8.7 Hz, 1H), 7.19–7.22 (m, 2H), 7.13 (d, J = 1.8 Hz, 1H), 6.95 (dd, J = 8.4 and 1.8 Hz, 1H), 6.86 (dd, J = 3.7 and 1.1 Hz, 1H), 6.74 (dd, J = 3.6 and 0.9 Hz, 1H), 6.36 (dd, J = 5.1 and 3.7 Hz, 1H), 6.27–6.30 (m, 2H), 5.99 (s, 1H), 3.07 (s, 3H). ^13^C NMR (600 MHz, CDCl_3_): δ 177.88, 163.50, 162.44, 156.60, 142.23, 139.64, 128.46, 128.00, 126.91, 126.33, 125.14, 123.97, 122.79, 122.66, 114.48, 114.20, 106.34, 55.49. HRMS (ESI) for C_20_H_15_O_3_S [M + H^+^]: calcd; 335.0742 found, 335.0750.

###### 5.1.3.10. 7–(2,3-dihydrobenzo[b][1,4]dioxin-6-yl)-2–(4-methoxyphenyl)-4H-chromen-4-one (10)

Percentage yield: 36%; HPLC purity: 97.65%; mp: 183–186 °C; ^1^H NMR (300 MHz, DMSO-*d_6_*): δ 8.14 (d, J = 9 Hz, 2H), 8.04–8.07 (m, 2H), 7.78 (dd, J = 8.4 and 1.8 Hz, 1H), 7.35–7.41 (m, 2H), 7.16 (d, J = 9.1 Hz, 2H), 7.03 (d, J = 8.3 Hz, 1H), 6.97 (s, 1H), 4.33 (s, 4H), 3.89 (s, 3H). ^13^C NMR (600 MHz, DMSO-*d_6_*): δ 177.22, 163.22, 162.63, 156.57, 145.52, 144.75, 144.31, 131.67, 128.70, 125.70, 123.91, 123.70, 122.16, 120.62, 118.20, 116.14, 115.71, 115.02, 105.90, 64.72, 64.56, 55.99. HRMS (ESI) for C_24_H_19_O_5_ [M + H+]: calcd; 387.1232 found, 387.1231.

###### 5.1.3.11. 2–(4-methoxyphenyl)-7-(quinolin-6-yl)-4H-chromen-4-one (11)

Percentage yield: 20%; HPLC purity: 95.76%; mp: 221–224 °C; ^1^H NMR (300 MHz, DMSO-*d_6_*): δ8.95 (s, 1H), 8.30 (d, J = 8.04, 1H), 8.24 (t, J = 8.8 Hz, 2H), 8.11 (s, 1H), 8.03 (d, J = 8.3 Hz, 1H), 7.90 (d, J = 8.2 Hz, 2H), 7.86 (s, 1H), 7.75 (d, J = 7.9 Hz, 1H), 7.46–7.48 (m, 1H), 7.02 (d, J = 8.4 Hz, 2H), 6.76 (s, 1H), 3.88 (s, 3H). ^13^C NMR (600 MHz, CDCl_3_): δ 178.06, 163.63, 162.50, 156.56, 150.89, 147.90, 145.67, 137.38, 136.61, 130.23, 128.84, 128.41, 128.02, 126.38, 124.34, 123.93, 123.00, 121.83, 116.45, 114.51, 106.38, 55.50. HRMS (ESI) for C_25_H_18_NO_3_ [M + H^+^]: calcd; 380.1287 found, 380.1347.

###### 5.1.3.12. 7-(1H-indol-5-yl)-2–(4-methoxyphenyl)-4H-chromen-4-one (12)

Percentage yield: 23%; HPLC purity: 97.35%; mp: 129–132 °C; ^1^H NMR (300 MHz, CDCl_3_) δ 8.36 (s, 1H), 8.30 (d, J = 8.4 Hz, 1H), 8.03 (t, J = 0.9 Hz, 1H), 7.98 (t, J = 3 Hz, 1H), 7.95 (t, J = 3 Hz, 1H), 7.85 (d, J = 1.5 Hz, 1H), 7.77 (dd, J = 8.4 and 1.8 Hz, 1H), 7.54–7.61 (m, 2H), 7.33 (t, J = 3.3 Hz, 1H), 7.05–7.10 (m, 2H), 6.80 (s, 1H), 6.68–6.70 (m, 1H), 3.94 (s, 3H); ^13^C NMR (600 MHz, CDCl_3_): δ 178.45, 163.44, 162.34, 156.67, 148.30, 136.02, 132.10, 132.04, 131.97, 128.54, 128.52, 128.46, 127.99, 125.83, 125.34, 124.49, 121.68, 119.83, 115.70, 114.45, 111.62, 106.26, 103.23, 55.49. HRMS (ESI) for C_24_H_18_NO_3_ [M + H^+^]: calcd; 368.1287 found, 368.1288.

###### 5.1.3.13. 2–(4-fluorophenyl)-7-phenyl-4H-chromen-4-one (13)

Percentage yield: 30%; HPLC purity: 98.50%; mp: 193–196 °C; ^1^H NMR (600 MHz, CDCl_3_): δ 8.27 (d, J = 8.4 Hz, 1H), 7.93–7.96 (m, 2H), 7.76 (d, J = 1.2 Hz, 1H), 7.65–7.69 (m, 3H), 7.50 (t, J = 7.8 Hz, 2H), 7.44 (m, 1H), 7.20–7.24 (m, 2H), 6.78 (s, 1H). ^13^C NMR (600 MHz, CDCl_3_): δ 178.13, 165.61, 163.93, 162.55, 156.55, 147.07, 139.11, 129.10, 128.72, 128.53, 128.47, 128.03, 128.01, 127.36, 126.19, 124.46, 122.58, 116.38, 116.24, 116.02, 107.52. 111.20. HRMS (ESI) for C_21_H_13_FO_2_ [M + H^+^]: calcd; 317.0900 found, 317.0994.

###### 5.1.3.14. 2–(4-fluorophenyl)-7-(furan-3-yl)-4H-chromen-4-one (14)

Percentage yield: 28%; HPLC purity: 98.07%; mp: 215–218 °C; ^1^HNMR (600 MHz, DMSO-*d_6_*): δ 8.42 (s, 1H), 8.15 (dd, J = 8.4 and 5.4 Hz, 2H), 8.02 (s, 1H), 7.99 (d, J = 8.4 Hz, 1H), 7.80 (s, 1H), 7.75 (d, J = 8.4 Hz, 1H), 7.41 (t, J = 8.7 Hz, 2H), 7.12 (s, 1H), 6.98 (s, 1H). ^13^C NMR (600 MHz, DMSO-*d_6_*): δ 177.17, 165.44, 163.78, 162.15, 156.75, 145.39, 141.97, 138.53, 129.49, 129.43, 128.17, 125.80, 125.05, 123.45, 122.13, 116.77, 116.63, 114.89, 109.12, 107.41. HRMS (ESI) for C_19_H_12_FO_3_ [M + H^+^]: calcd; 307.0770 found, 307.0858.

###### 5.1.3.15. 2–(4-fluorophenyl)-7-(thiophen-2-yl)-4H-chromen-4-one (15)

Percentage yield: 49%; HPLC purity: 96.74%; mp: 202–205 °C; ^1^H NMR (300 MHz, DMSO-*d_6_*): δ 8.22–8.27 (m, 2H), 8.12 (d, J = 1.5 Hz, 1H), 8.07 (d, J = 7.8 Hz, 1H), 7.83–7.84 (m, 1H), 7.81 (d, J = 1.5 Hz, 1H), 7.76 (dd, J = 5.1 and 1.2 Hz, 1H), 7.46 (t, J = 8.9 Hz, 2H), 7.26 (dd, J = 5.1 and 3.7 Hz, 1H), 7.06 (s, 1H). ^13^C NMR (600 MHz, DMSO-*d_6_*): δ 177.13, 165.48, 163.82, 162.32, 156.66, 141.55, 139.75, 129.61, 129.48, 128.68, 128.05, 126.85, 126.24, 123.22, 122.35, 116.76, 116.61, 114.54, 107.36. HRMS (ESI) for C_19_H_12_FO_2_S [M + H^+^]: calcd; 323.0542 found, 323.0586.

###### 5.1.3.16. 7–(2,3-dihydrobenzo[b][1,4]dioxin-6-yl)-2–(4-fluorophenyl)-4H-chromen-4-one (16)

Percentage yield: 63%; HPLC purity: 98.13%; mp: 193–196 °C; ^1^H NMR (300 MHz, DMSO-*d_6_*): δ 8.22–8.27 (m, 2H), 8.05–8.08 (m, 2H), 7.79 (dd, J = 8.1 and 1.5 Hz, 1H), 7.40–7.50 (m, 2H), 7.35–7.39 (m, 2H), 7.06 (s, 1H), 7.03 (d, J = 8.1 Hz, 1H), 4.33 (s, 4H). ^13^C NMR (600 MHz, DMSO-*d_6_*): δ 177.34, 165.45, 163.79, 162.19, 156.59, 145.70, 144.78, 144.30, 131.57, 129.53, 129.47, 128.15, 125.73, 124.05, 122.06, 120.61, 118.20, 116.74, 116.59, 116.13, 115.75, 107.29, 64.72, 64.56. HRMS (ESI) for C_23_H_16_FO_4_ [M + H^+^]: calcd; 375.1033 found, 375.1026.

###### 5.1.3.17. 2–(4-fluorophenyl)-7-(quinolin-6-yl)-4H-chromen-4-one (17)

Percentage yield: 33%; HPLC purity: 95.15%; mp: 236–240 °C; ^1^H NMR (600 MHz, acetone): δ 8.50 (dd, J = 4.2 and 1.8 Hz, 1H), 8.08 (d, J = 1.8 Hz, 1H), 8.03 (d, J = 7.2 Hz, 1H), 7.86 (d, J = 1.2 Hz, 1H), 7.78–7.82 (m, 3H), 7.71 (dd, J = 8.4 and 3.0 Hz, 2H), 7.56 (dd, J = 8.3 and 1.7 Hz, 1H), 7.16 (dd, J = 8.2 and 4.1 Hz, 1H), 7.01 (t, J = 8.9 Hz, 2H), 6.63 (s, 1H). ^13^C NMR (600 MHz, acetone) δ 176.87, 163.42, 161.90, 156.21, 151.36, 147.62, 144.96, 136.65, 135.87, 129.82, 129.65, 129.16, 129.10, 128.49, 128.12, 126.84, 125.61, 124.37, 122.39, 122.19, 116.68, 116.37, 116.23, 107.09. HRMS (ESI) for C_24_H_15_FNO_2_ [M + H^+^]: calcd; 368.1087 found, 368.0993.

###### 5.1.3.18. 2–(4-fluorophenyl)-7-(1H-indol-5-yl)-4H-chromen-4-one (18)

Percentage yield: 50%; HPLC purity: 95.15%; mp: 229–232 °C; ^1^H NMR (300 MHz, DMSO-*d_6_*): δ 11.29 (s, 1H), 8.26 (dd, J = 9.1 and 5.4 Hz, 2H), 8.05–8.14 (m, 3H), 7.88 (dd, J = 8.4 and 1.7 Hz, 1H), 7.61 (dd, J = 8.6 and 1.7 Hz, 1H), 7.56 (d, J = 8.6 Hz, 1H), 7.42–7.51 (m, 3H), 7.05 (s, 1H), 6.57 (s, 1H). ^13^C NMR (600 MHz, DMSO-*d_6_*): δ 177.47, 165.43, 163.77, 162.16, 156.71, 148.24, 136.69, 129.54, 129.48, 129.46, 128.77, 127.03, 125.66, 124.54, 121.56, 120.93, 119.57, 116.75, 116.60, 115.76, 112.58, 107.27, 102.38. HRMS (ESI) for C_23_H_15_FNO_2_ [M + H^+^]: calcd; 356.1087 found, 356.1098.

###### 5.1.3.19. 7-(1H-indol-5-yl)-2–(4-nitrophenyl)-4H-chromen-4-one (19)

Percentage yield: 23%; HPLC purity: 96.08%; mp: 255–258 °C; ^1^H NMR (600 MHz, DMSO-*d_6_*): δ 11.23 (s, 1H), 8.35–8.39 (m, 4H), 8.06 (dd, J = 18.6 and 10.2 Hz, 3H), 7.86 (d, J = 8.4 Hz, 1H), 7.57 (dd, J = 8.4 and 1.8 Hz, 1H), 7.52 (d, J = 9 Hz, 1H), 7.38–7.39 (m, 1H), 7.17 (s, 1H), 6.52–6.53 (m, 1H). ^13^C NMR (600 MHz, DMSO-*d_6_*): δ 177.49, 160.68, 156.76, 149.47, 148.56, 137.64, 136.74, 129.33, 128.78, 128.21, 127.06, 125.73, 124.80, 124.52, 121.62, 120.93, 119.62, 115.81, 112.62, 109.60, 102.42, 60.25, 21.17, 14.49. HRMS (ESI) for C_23_H_15_N_2_O_4_ [M + H^+^]: calcd; 383.1032 found, 383.1032.

###### 5.1.3.20. 7-(Quinolin-6-yl)-2–(3,4,5-trimethoxyphenyl)-4H-chromen-4-one (20)

Percentage yield: 26%; HPLC purity: 98.92%. mp: 214–217 °C; ^1^H NMR (300 MHz, DMSO-*d_6_*): δ 8.99 (dd, J = 4.2 and 1.8 Hz, 1H), 8.50–8.55 (m, 2H), 8.38 (d, J = 1.5 Hz, 1H), 8.29 (dd, J = 8.8 and 2.1 Hz, 1H), 8.17–8.21 (m, 2H), 8.02 (dd, J = 8.3 and 1.7 Hz, 1H), 7.64 (dd, J = 8.3 and 4.2 Hz, 1H), 7.47 (s, 2H), 7.23 (s, 1H), 3.97 (s, 6H), 3.79 (s, 3H). ^13^C NMR (600 MHz, CDCl_3_): δ 178.05, 163.48, 156.58, 153.62, 151.11, 148.11, 145.98, 141.37, 137.22,136.43, 130.41, 128.74, 128.39, 126.88, 126.44, 126.42, 124.58, 122.94, 121.87, 116.53, 107.58, 103.82, 61.04, 56.39. HRMS (ESI) for C_27_H_22_NO_5_ [M + H^+^]: calcd; 440.1498 found, 440.1588.

###### 5.1.3.21. 7-(1H-indol-5-yl)-2–(3,4,5-trimethoxyphenyl)-4H-chromen-4-one (21)

Percentage yield: 22%; HPLC purity: 95.09%; mp: 246–250 °C; ^1^H NMR (300 MHz, DMSO-*d_6_*): δ 11.30 (s, 1H), 8.19 (d, J = 1.5 Hz, 1H), 8.08–8.11 (m, 2H), 7.88 (dd, J = 8.4 and 1.5 Hz, 1H), 7.61–7.64 (m, 1H), 7.56 (d, J = 8.4 Hz, 2H), 7.47 (s, 2H), 7.18 (s, 1H), 6.57 (s, 1H), 3.96 (s, 6H), 3.78 (s, 3H). ^13^C NMR (600 MHz, DMSO-*d_6_*): δ 177.54, 162.84, 156.71, 153.71, 148.10, 141.01, 136.68, 129.50, 128.75, 127.02, 126.95, 125.59, 124.45, 121.60, 120.98, 119.55, 115.91, 112.54, 107.22, 104.51, 102.36, 60.68, 56.77. HRMS (ESI) for C_26_H_22_NO_5_ [M + H^+^]: calcd; 428.1498 found, 428.1456.

### 5.2. Cell culture

Ishikawa cells (a human endometrial carcinoma of the epithelial cell) were kindly gifted by Dr. Geetanjali Sachdeva and Dr. Uddhav Chaudhari of National Institute for Research In Reproductive and Child Health (NIRRCH), Mumbai. They had procured the cells from Sigma‐Aldrich (St. Louis, MO) (cat #99040201). The cells were maintained in RPMI medium supplemented with 10% Foetal bovine serum (FBS) and 100 IU/mL penicillin. The cell lines were maintained under standard cell culture conditions at 37 °C and 5% CO_2_ in a humidified environment.

### 5.3. Cell viability assay

Mitochondrial activity was evaluated by MTT assay.^59^ This assay is based on enzymatic reduction of the yellow-colored MTT dye to purple-colored formazan crystals by a variety of mitochondrial and cytosolic enzymes that are operational in viable cells. Briefly, ISHIKAWA (3000 cells/well) were seeded in 100 µL of medium into a 96-well plate and left to settle in a CO_2_ incubator. The test compound **14** was added in each well (100 μL/well) with different concentrations and the plate was incubated for 48 h. Four hours before the end of the incubation period, 20 μL of MTT solution (2.5 mg/mL in PBS) was added to each well and re-incubated for 4 h at 37 °C. Then 150 μL of DMSO was added to each well to dissolve the formazan crystals. The optical density (OD) of each well was recorded using a microplate reader at a wavelength of 570 nm. The percentages of cell viability and growth inhibition were calculated according to the following equations. Cell viability (%) = [(OD of treated cells-OD of blank)/(OD of control-OD of blank) ×100].

### 5.4. Identification of nuclear and cellular morphology

Ishikawa was treated with compounds **14** at concentrations of 0.1, 0.5, and 1 μM for 24 h. Briefly, cells were collected, washed twice with PBS, and fixed with methanol: acetic acid (3:1 ratio v/v). The next day, cells were washed and dispensed in 50 μL of fixing solution. Cells were then spread on clean slides, and exposed to DAPI solution (4′,6-diamidino-2-phenylindole(DAPI) (CAS Number: 28718–90-3) (5 μg/mL in 0.01 M citric acid and 0.45 M disodium phosphate containing 0.05% Tween 20) for 30 min at room temperature. The slides were then rinsed with distilled water for 10 min, and washed with PBS. After drying, the slides were mounted with glycerol: PBS (1:1 ratio v/v) and observed under the fluorescent microscope to figure out any nuclear morphological changes that occur during apoptosis.^60^

### 5.5. Measurement of mitochondrial membrane potential

Rhodamine staining was employed to investigate apoptosis induced by compound **14** in Ishikawa cells. Under physiological conditions, rhodamine dyes are known to accumulate within active and polarised mitochondria, resulting in robust fluorescence signals. However, when the mitochondrial transmembrane potential is compromised the dyes fail to accumulate within the mitochondria, leading to a decline in rhodamine fluorescence intensity. The Rhodamine staining protocol followed was as follows: Ishikawa cells were treated at 0.1, 0.5, and 1 μM for 24 h, and RH-123 (1 μg) (Cat No. R8004) was added 30 min before the termination of the experiment and then washed with PBS before analysis on a fluorescence microscope.^61^

### 5.6. Acridine orange staining

The Autophagy induction ability of compound **14** was assessed by acridine orange staining which is used to measure the autophagic acidic vesicular organnelles. Ishikawa cell were seeded in a six-well plate and treated with compound **14** for 48 h. Incubation of the cells was done with 1 mg/mL Acridine orange (Cat no. A9231**)** before the termination of the experiment. Cells were then washed with PBS and then analysed with a fluorescence microscope. In untreated or basal conditions, the cells exhibited predominantly green fluorescence, indicating a neutral pH environment within the cytoplasm. However, upon induction of autophagy, an increase in red fluorescence was observed, suggesting the presence of acidic compartments, such as autophagosomes and lysosomes. A9231.

### 5.7. Measurement of ROS

Dichlorodihydrofluorescin diacetate (DCFHDA) is a fluorogenic dye that measures hydroxyl, peroxyl, and other reactive oxygen species (ROS) activity within the cell. The oxidative conversion of DCFH-DA to DCF (2′, 7′ -dichlorofluorescein) leads to the detection of intracellular ROS. As such, an increase in intracellular ROS is indicative of apoptosis in cancer cells Ishikawa cells were treated with compound-**14** at 100, 500, and 1000 nM concentrations for 24 h. After 24h cells were stained with DCFHDA (20 µM) (Cat no, 287810) for 45 min. After 45 min, cells were washed with PBS buffer and analysed with a fluorescence microscope. Low green Fluorescence was observed in untreated control cells while bright green fluorescence was observed with treated cells.^61^

### 5.8. Preparation of cell lysate and Western blotting

Ishikawa cells were treated with compound-**14** at 100, 500, and 1000 nM concentrations for 24 h. After 24h cells were collected and centrifuged at 400 g, washed in PBS and cell pellets were lysed in RIPA buffer in preparation of the whole cell lysate. Supernatants were collected and proteins were estimated using the Bradford reagent. An equal amount of protein (60–80 µg) was loaded into each well for SDS-PAGE. Blocking was done with 5% BSA at room temperature with shaking for 2 h. After the SDS page run was completed, we washed the PVDF membrane and then blocking was done. Blots were incubated with different primary antibodies(1:1000 dilutions), and secondary antibodies (1:10 000 dilutions) and chemiluminescence was captured on Chemidoc after incubating the blots in ECL plus solution.

Primary Antibodies: LC3B Antibody (Cell signalling Technology, Cat No:2775), Cyclin B1 (Cell signalling Technology, Cat No:4138), SKP-2 (Cell signalling Technology, Cat No:4358), CDK1 (Abcam, Anti-CDK1 antibody [A17]), cdc25c (Cell signalling Technology, Cat No:4688); internal control: Actin (Sigma Aldrich, Cat No: A5441);

Secondary antibodies: Anti-mouse IgG, HRP-linked Antibody #7076, Cell signalling Technology, Dilution 1:10 000 and Anti-rabbit IgG, HRP-linked Antibody #7074, Cell signalling Technology, Dilution 1:10 000.

### 5.9. PARP inhibitory assay

Enzyme inhibition assays were performed by the Reaction Biology Corporation, Malvern, PA. (http://www.reactionbiology.com). PARP enzymatic activity transfers ADP-ribose from NAD + substrate into the PAR chain on histone substrate. PARP enzymatic activity transfers ADP-ribose from NAD + substrate into the PAR chain on histone substrate. The compounds were tested in a 10-dose IC50 singlet with a 3-fold serial dilution starting at 10 uM against 2 PARPs. Control compound, Veliparib (ABT-888) and olaparib was tested in a 10-dose IC50 with a 3-fold serial dilution starting at 0.1 uM. Raw data in data tabs represent luminescence signals corresponding to the amount of NAD + utilised in the enzymatic reaction. The control compound, Veliparib (ABT-888), was tested in a 10-dose IC50 with a 3-fold serial dilution starting at 0.1 uM. The substrates used were: Activated DNA, 10 µg/mL; NAD+ (Nicotinamide Adenine Dinucleotide), 500 nM; Protein substrate for PARP1, core histones from chicken, 10 µg/mL; protein substrate for PARP2, 5 µM.

### 5.10. In vitro microtubule assembly assay

The assay was performed using the tubulin polymerisation kit (BK006P,Cytoskeleton). Pure tubulin (cat. # T240) was equilibrated in 80 mM PIPES, 1 mM EGTA, 1 mM MgCl2, pH 6.8 buffer (BRB80) and centrifuged at 75 000 rpm for 10 min in an TLA-100 rotor in a centrifuge. The tubulin concentration was measured spectrophotometrically at 275 nm (ε = 107.000 M-1 cm-1) and then diluted to 10 μM. The samples were supplemented with 1 mM GTP (cat. #BST05) and the compound 14 at 0.1, 1, 5, 10 μM (or the vehicle, i.e. DMSO). The time course of assembly at 37 °C was detected as turbidity by measuring the absorbance of the samples at 350 nm using a spectrophotometer.

### 5.11. Docking study

The crystal structures of PARP1, PARP2, and tubulin (with PDB IDs: 5DS3, 4TVJ and 1SA0, respectively) were retrieved from the protein data bank (https://www.rcsb.org).^62^ All the crystal structures were prepared individually by removing existing ligands, water molecules, unbound ions, and extra chains using the PyMOL molecular graphics tool (Schrödinger, LLC). Thereafter, non-polar hydrogens were merged while polar hydrogens were added to each protein added using AutoDock tools.^63^ This process was repeated for each protein and then saved into docking-ready PDBQT format. 2-D chemical structures of all the ligands (standards as well as test compounds) were drawn using MarvinSketch 18.5.0 (ChemAxon Ltd.) and converted into 3D conformation (mol2 format) using Frog2 server.^64^ Polar hydrogens were added while non-polar hydrogens were merged with the carbons and the internal degrees of freedom and torsions were set. The ligand molecules were further saved into the docking-ready PDBQT format using AutoDock tools.^63^ Docking of the ligands to various protein targets and determination of binding affinities was carried out using AutoDock Vina.^65^

### 5.12. Drug-likeness and prediction of ADME

*In silico* methods for the determination of absorption, distribution, metabolism, and excretion (ADME) parameters depend on theoretically derived statistical models, which have been generated by relating the structural characteristics of compounds that have been measured in a given assay to their biological responses and are now widely used due to its low resource necessity.^66^ Therefore, test compounds were evaluated for the ADME parameters by using the SwissADME tool.^67^ and accessed using Lipinski’s rule of 5 for drug-likeness.^68^

### 5.13. Cell cycle analysis

Cells were treated with compound **14** (10, 50, 100, 500 and 1000 nM) for 24 h, collected and washed once with PBS. The pellet was then fixed in 70% ethanol overnight, followed by one washing with PBS and incubation with RNAse A for 90 min at 37 °C. Cells were then stained with PI and acquired in FACS. Analysis of the data was done by Modfit software (Verity Software House Inc., Topsham, ME) for the proportions of different cell cycle phases.^69^

## Supplementary Material

Supplemental Material
