# Supplementary material for: Flavone-based dual PARP-Tubulin inhibitor manifesting efficacy against endometrial cancer
Source: J Enzyme Inhib Med Chem. 2023 Nov 2;38(1):2276665. doi: 10.1080/14756366.2023.2276665 (PMC10627047; doi:10.1080/14756366.2023.2276665)

# Stitching a heteroaryl arm on the flavone framework culminates in a tractable dual PARP-tubulin inhibitor manifesting anti-endometrial cancer activity

**Contents:**

**1. $^{1}H$ NMR for compounds 1-21 ……………..…………………………………………..…S1-21**

**2. $^{13}C$ NMR for compounds 1-21……………………...…………………………………...S22-42**

**3. HPLC purity data for compounds 1-21….…………………………………………..S43-63**

**4. HRMS for compounds 1-21……………….…………………………..……………….S64-84**

## $^{1}H$ NMR of Compound 1

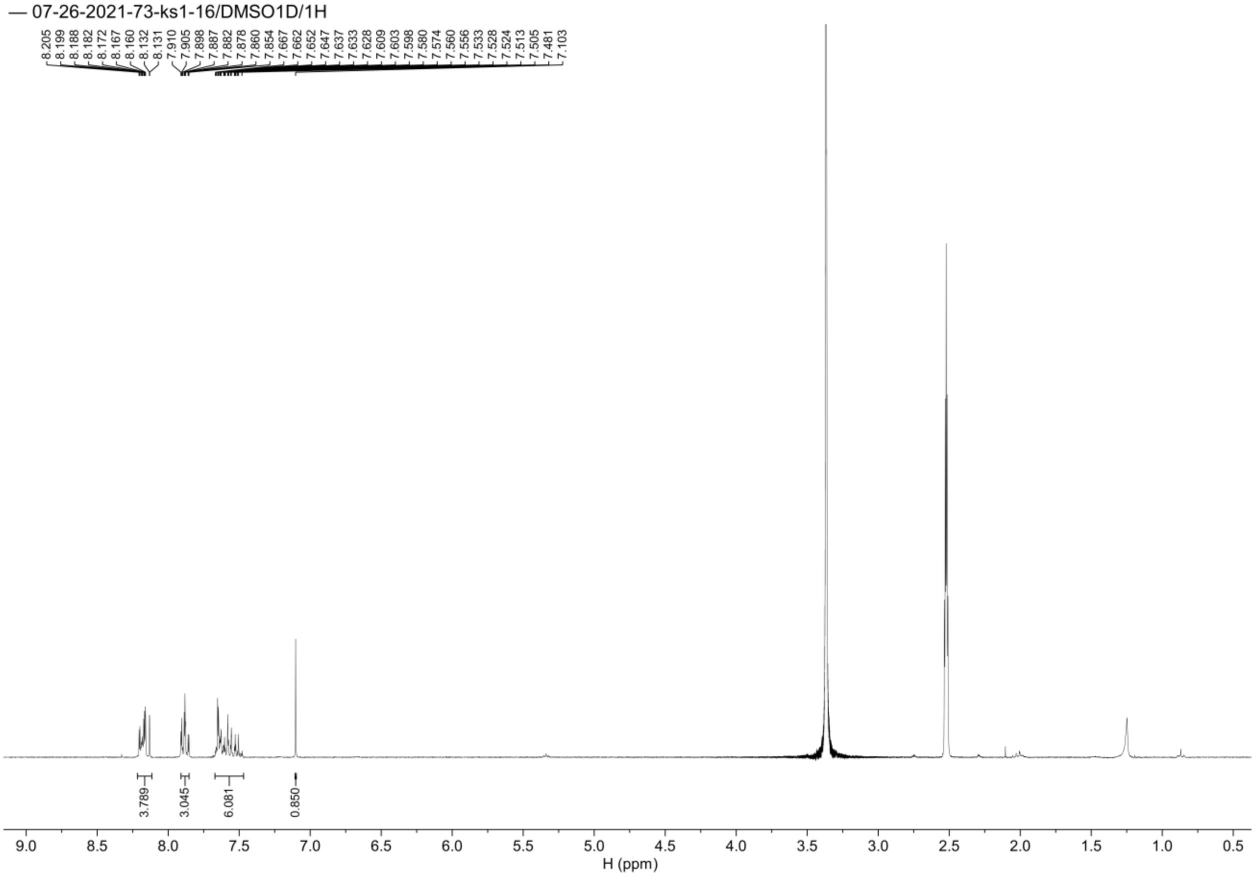

# $^{1}$H NMR of Compound 2

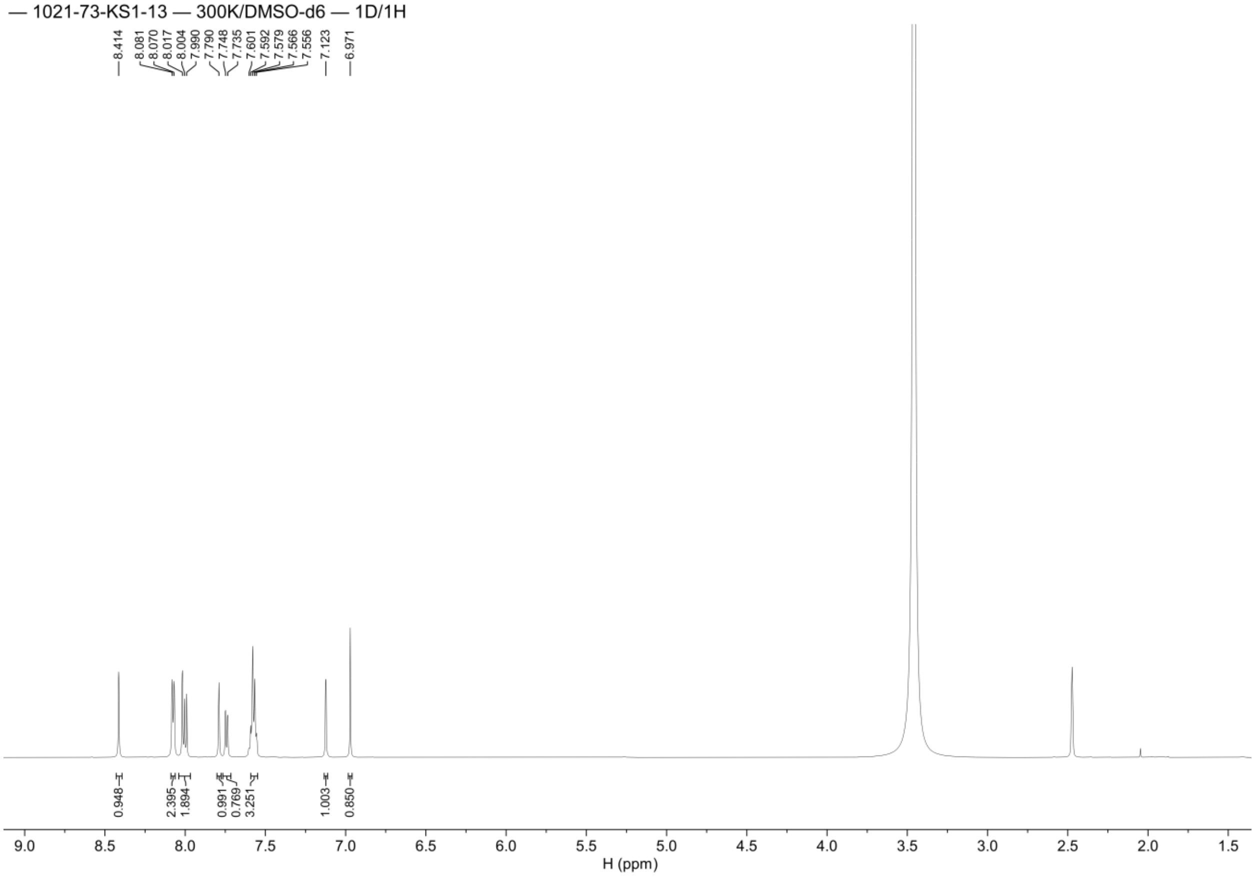

# $^{1}$H NMR of Compound 3

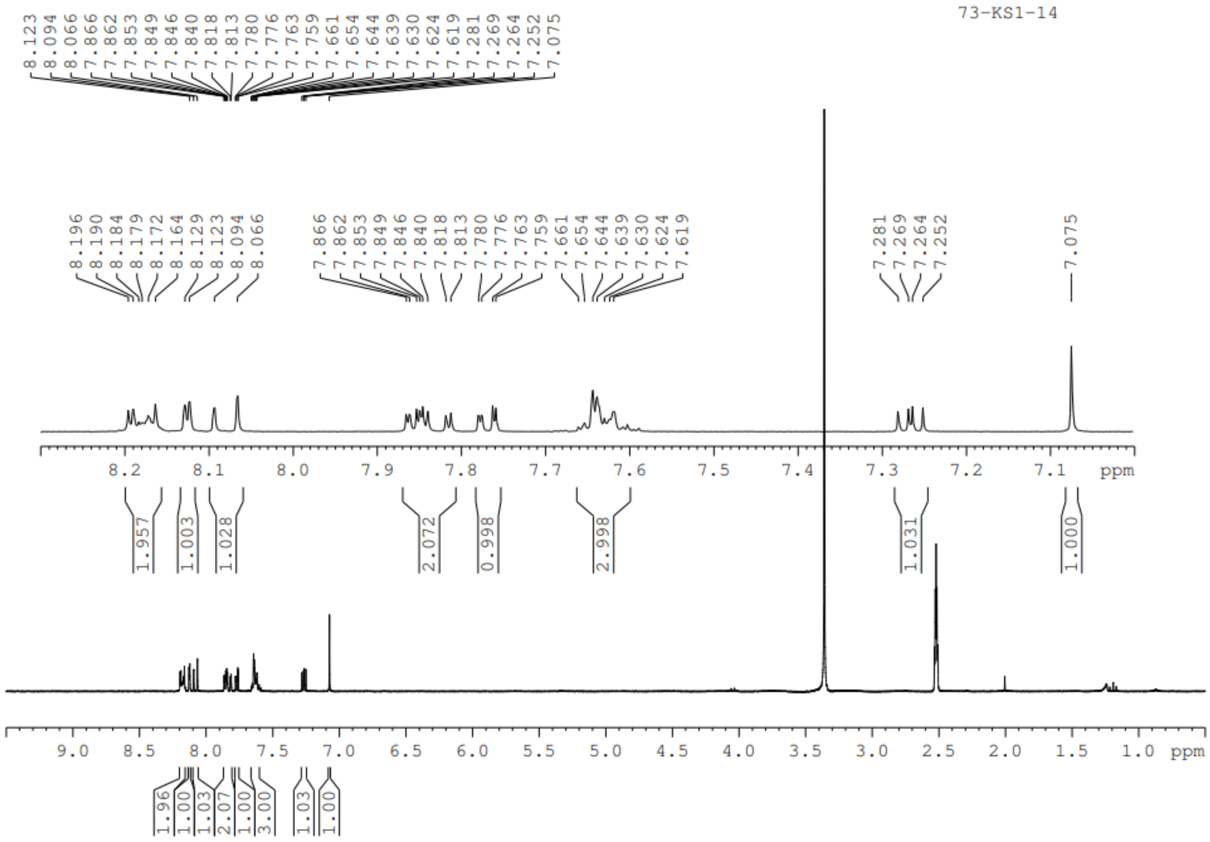

# $^1$H NMR of Compound 4

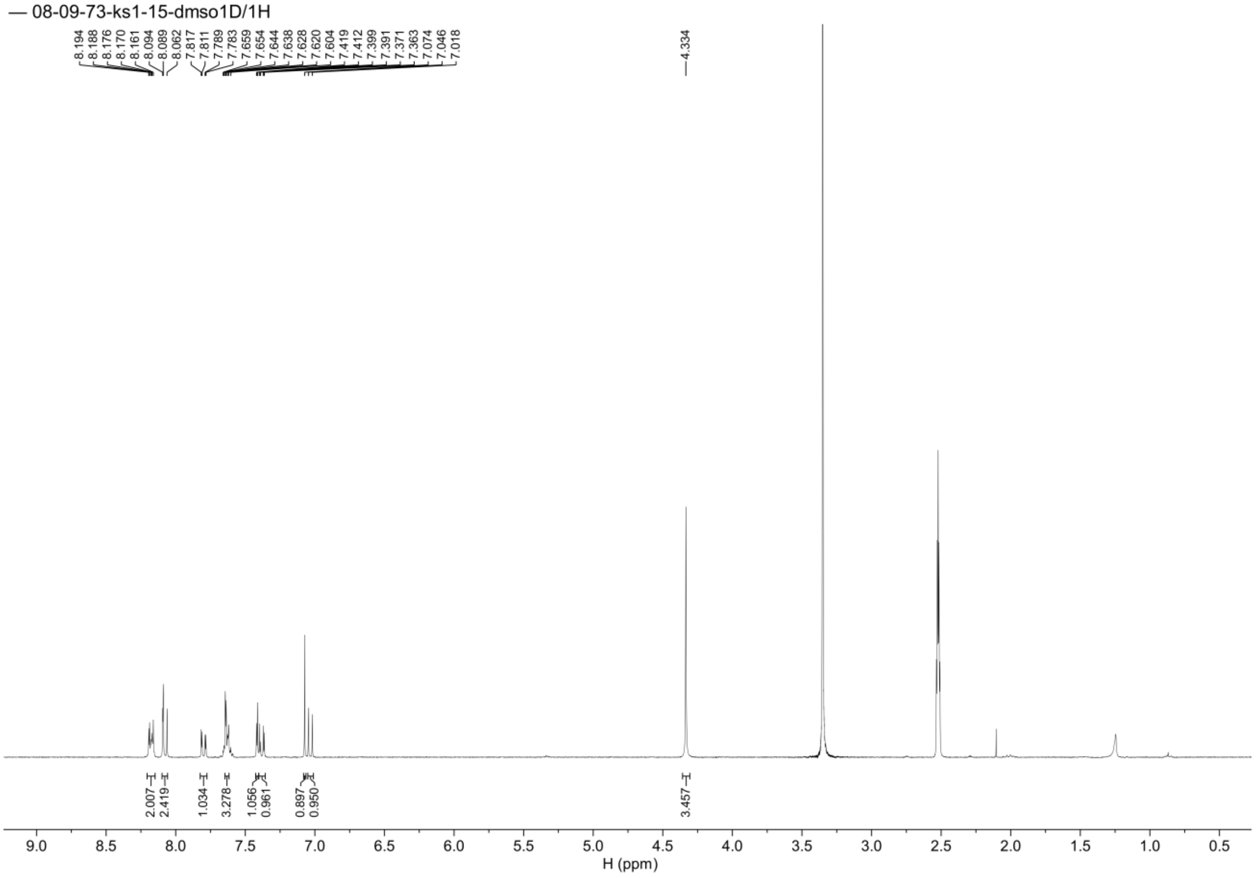

# $^1$H NMR of Compound 5

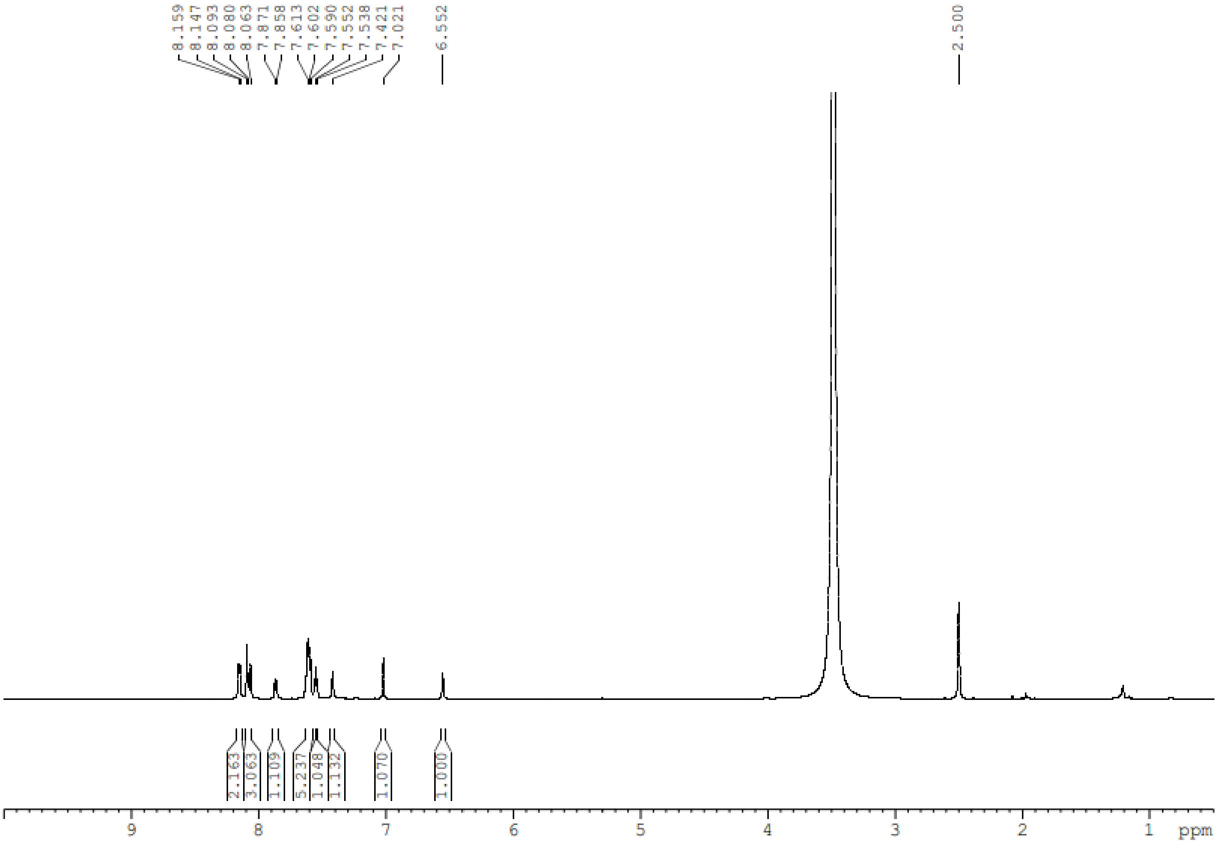

# $^1$H NMR of Compound 6

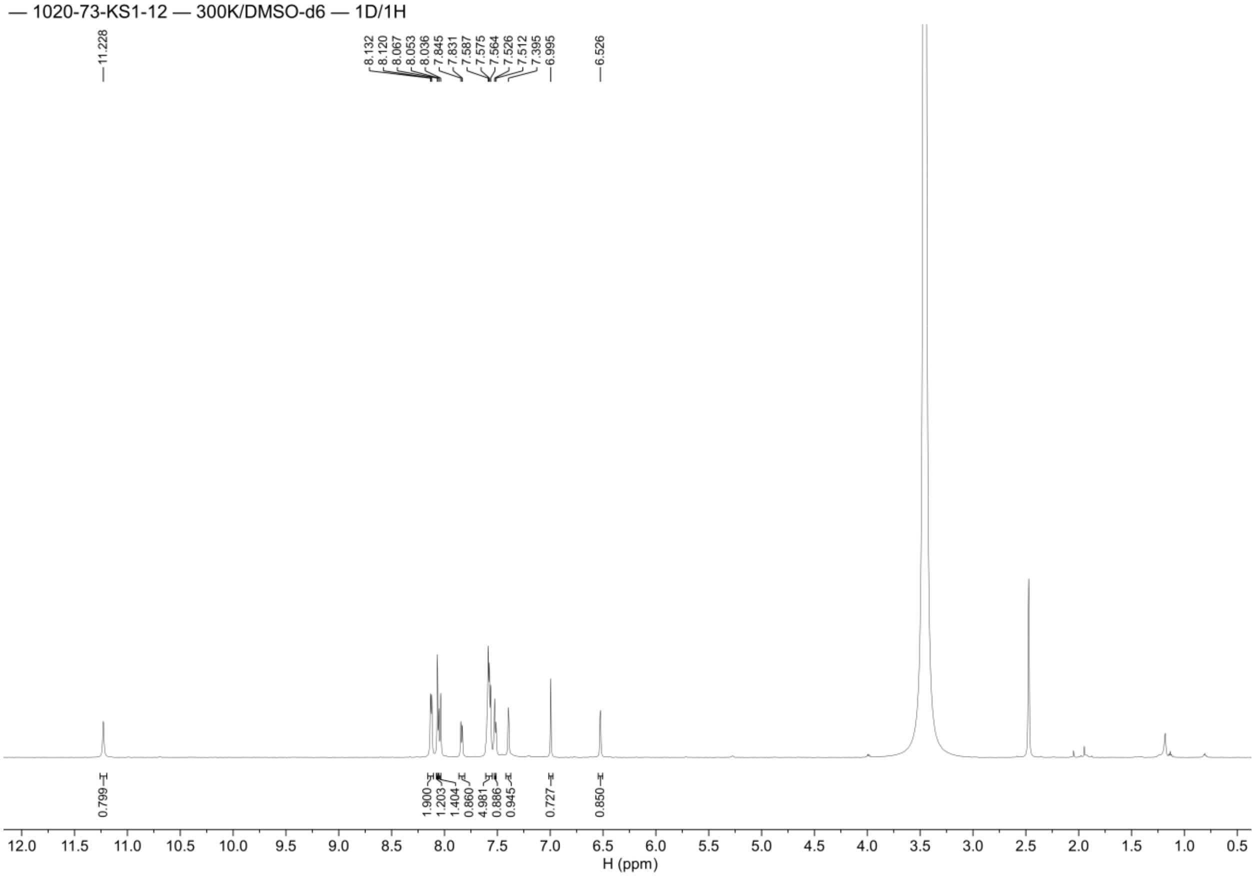

# $^{1}$H NMR of Compound 7

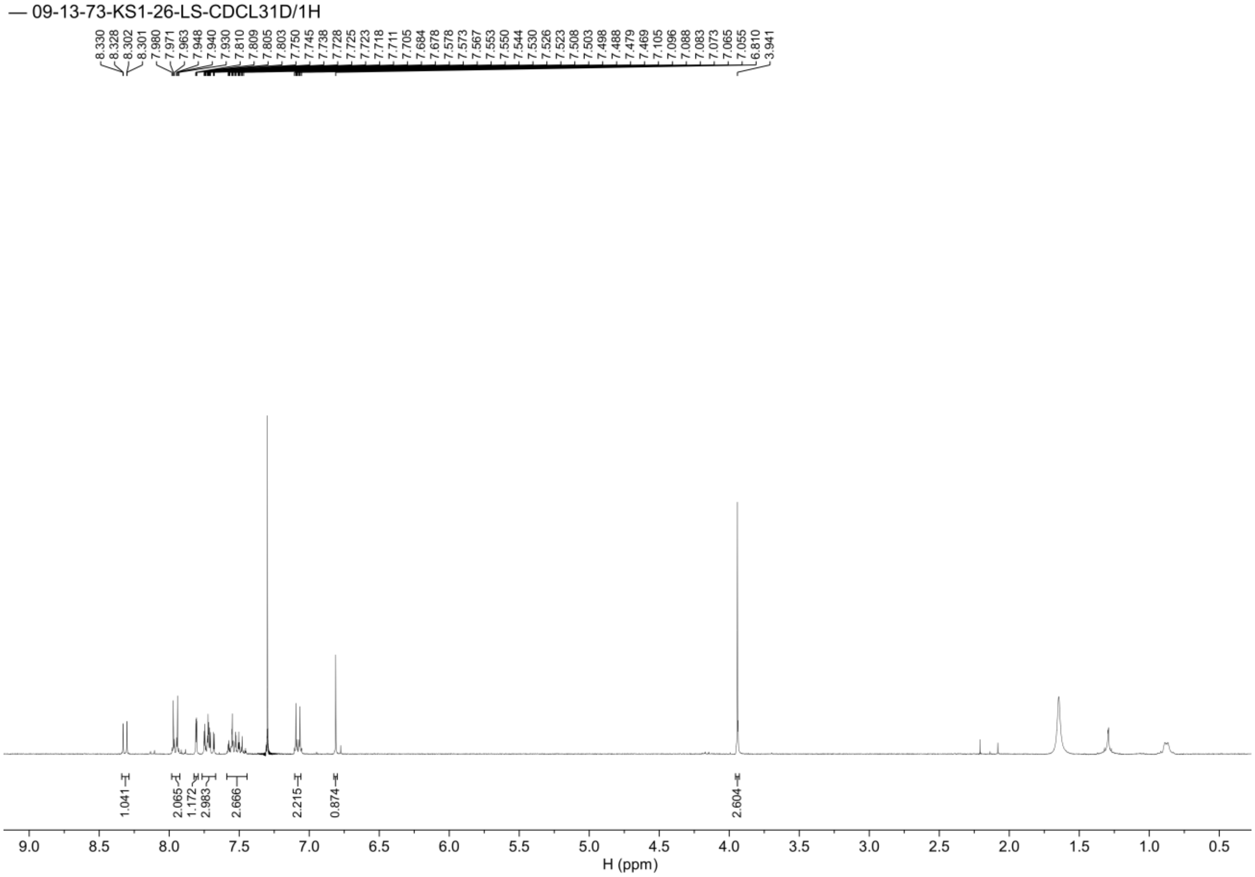

## $^{1}$H NMR of Compound 8

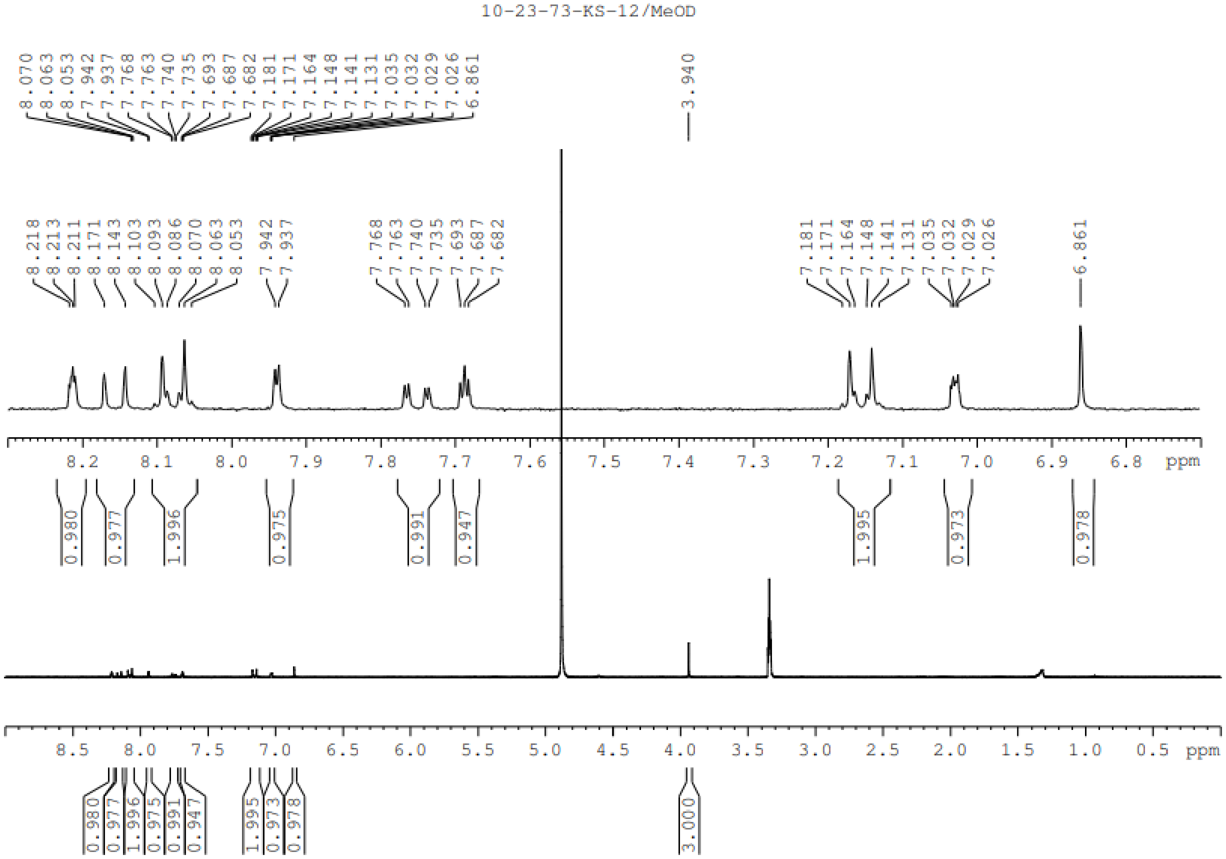

# $^{1}$H NMR of Compound 9

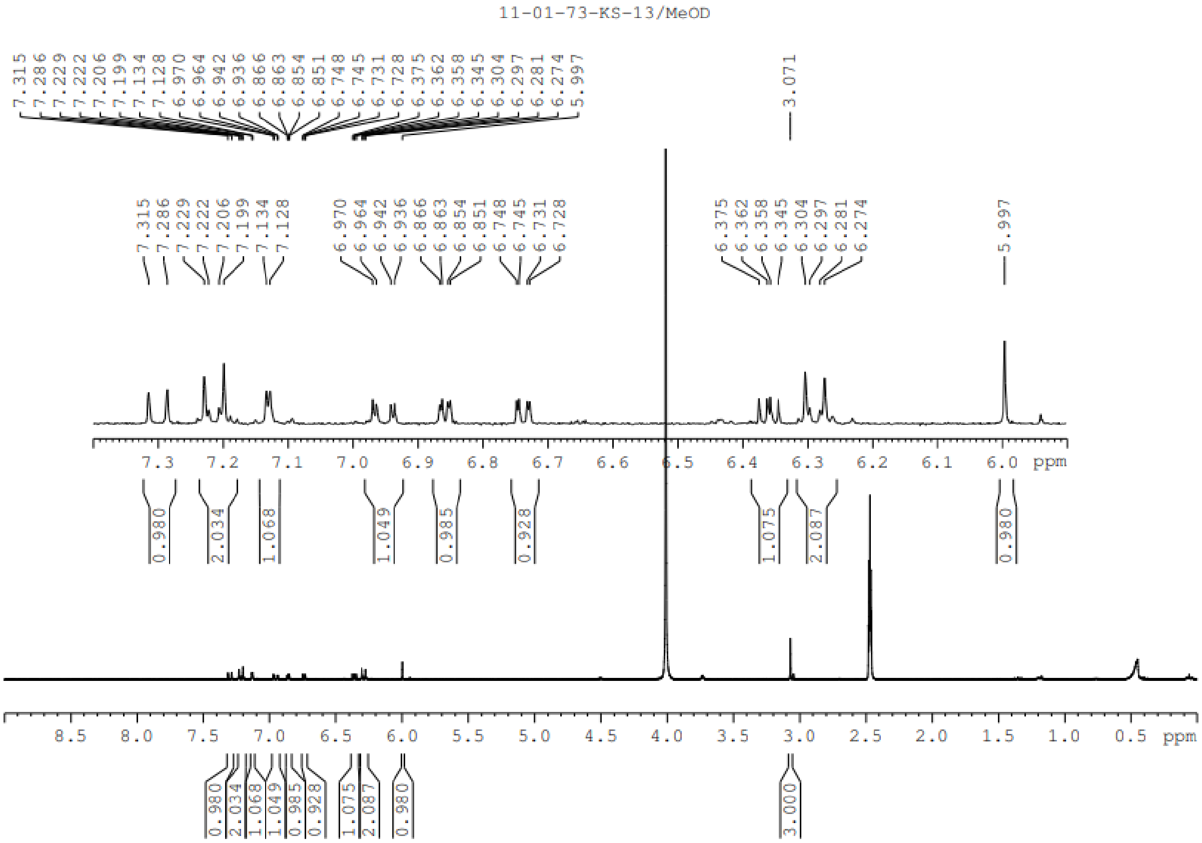

# $^{1}$H NMR of Compound 10

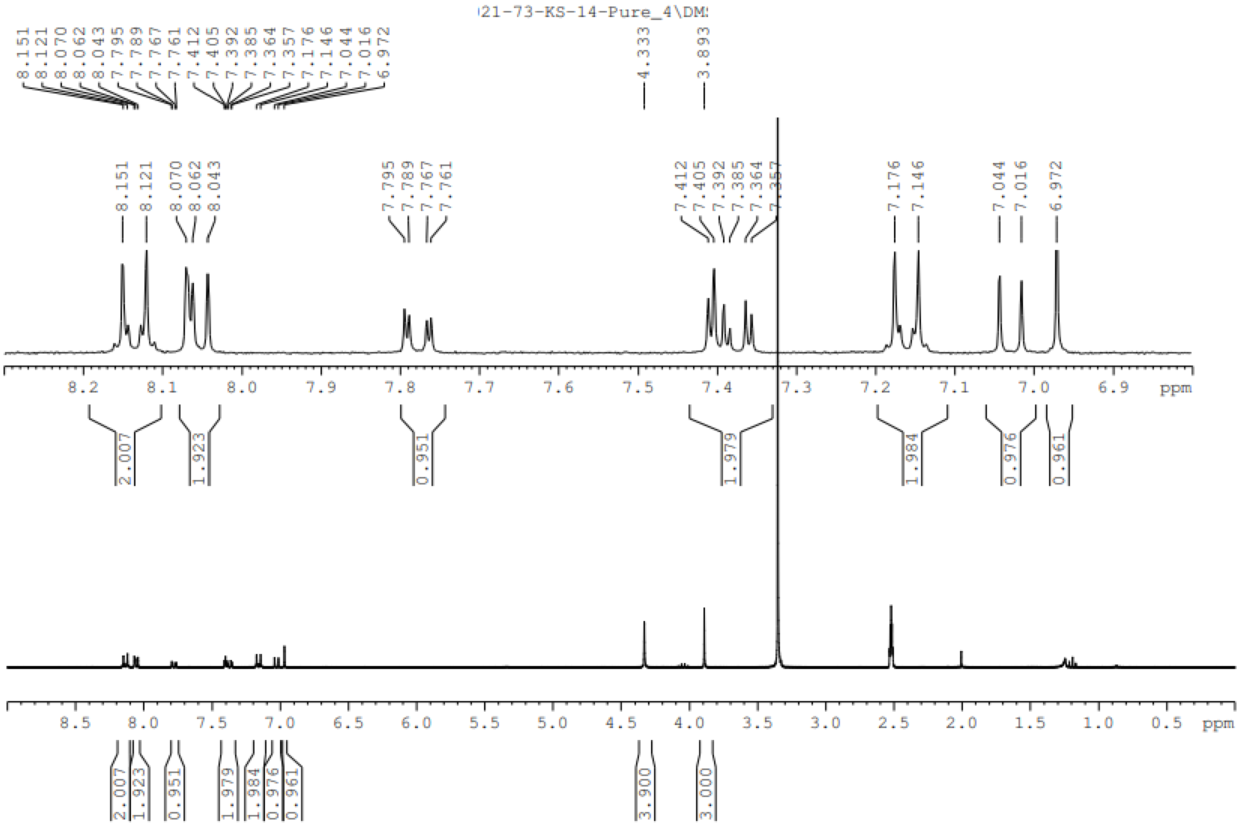

## $^{1}$H NMR of Compound 11

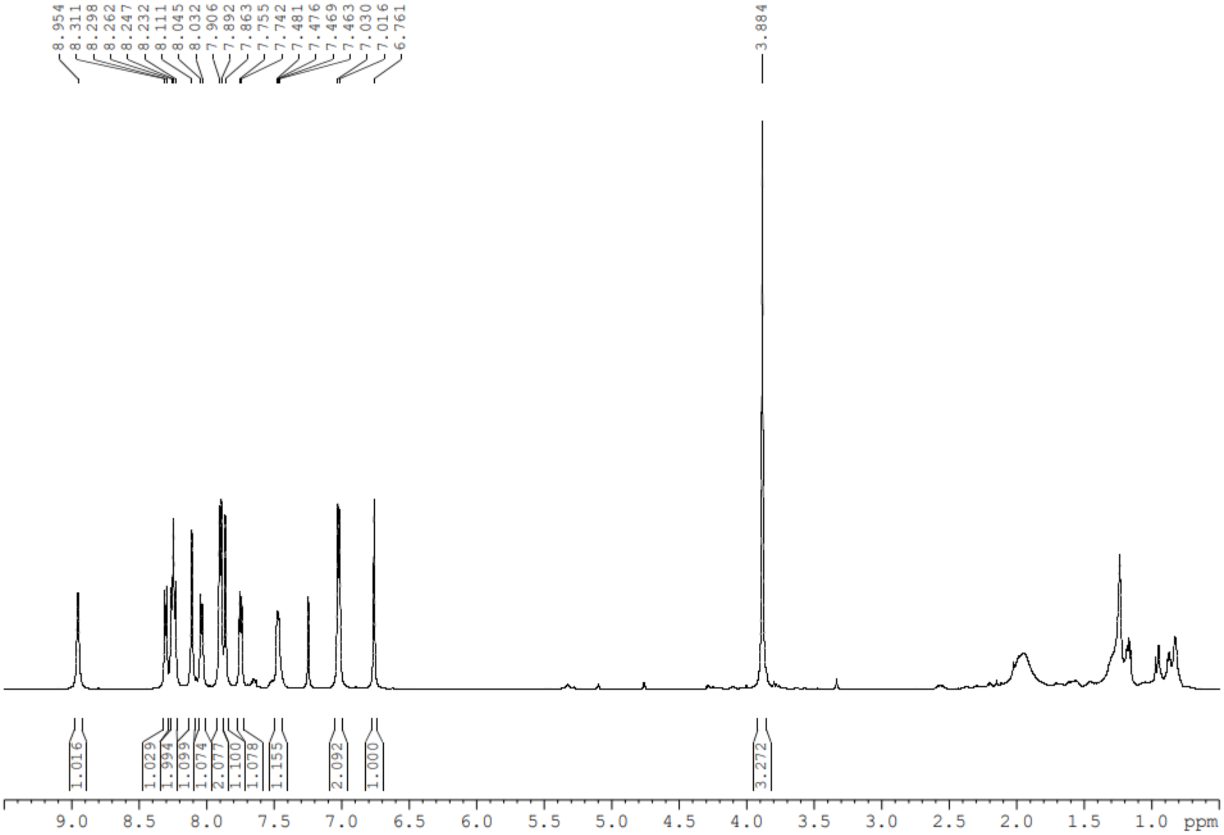

# $^{1}$H NMR of Compound 12

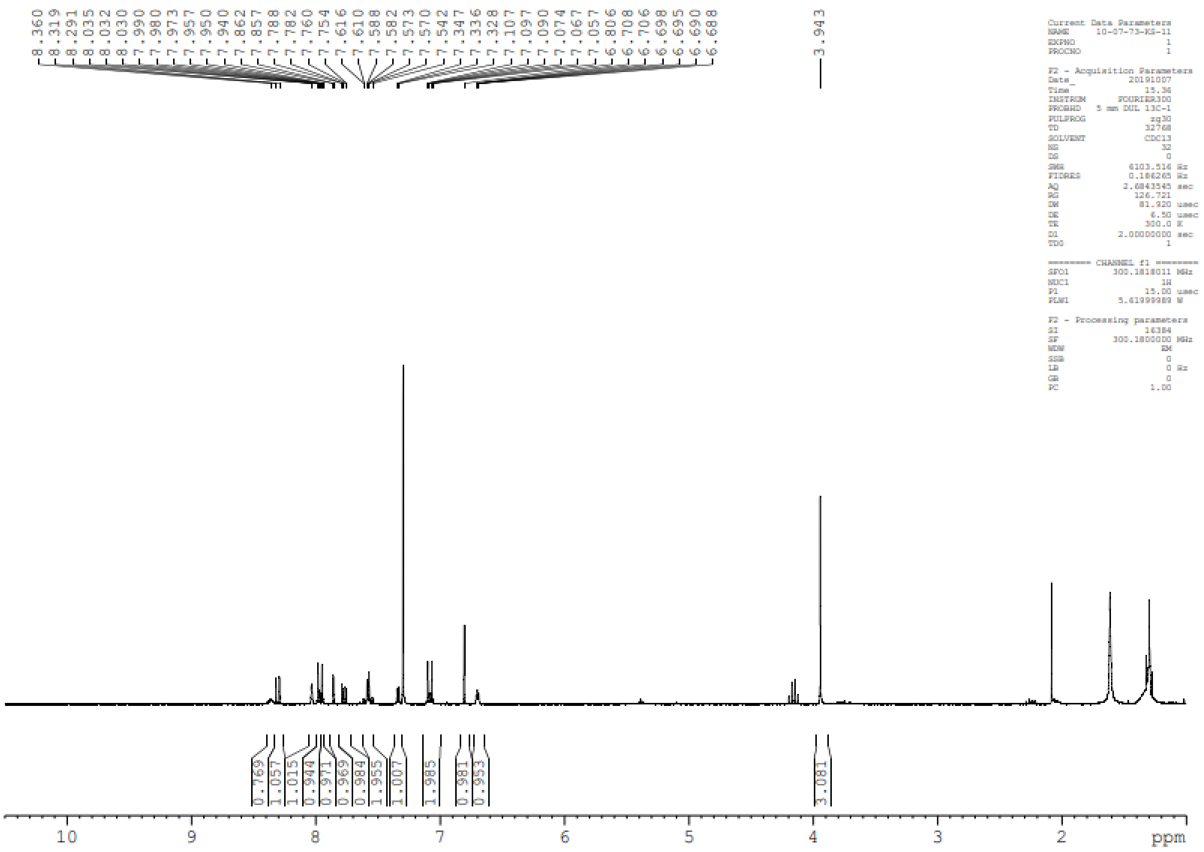

# $^1$H NMR of Compound 13

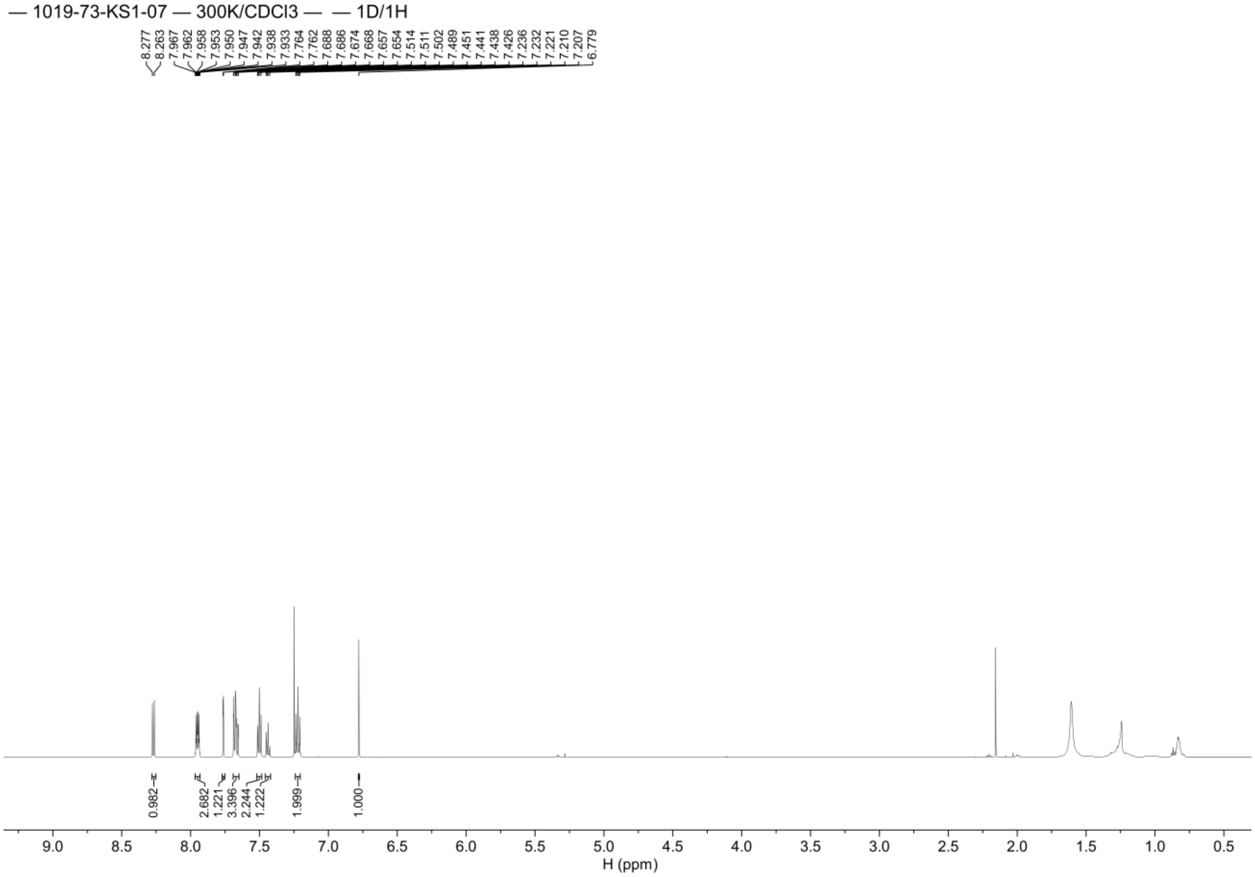

# $^{1}$H NMR of Compound 14

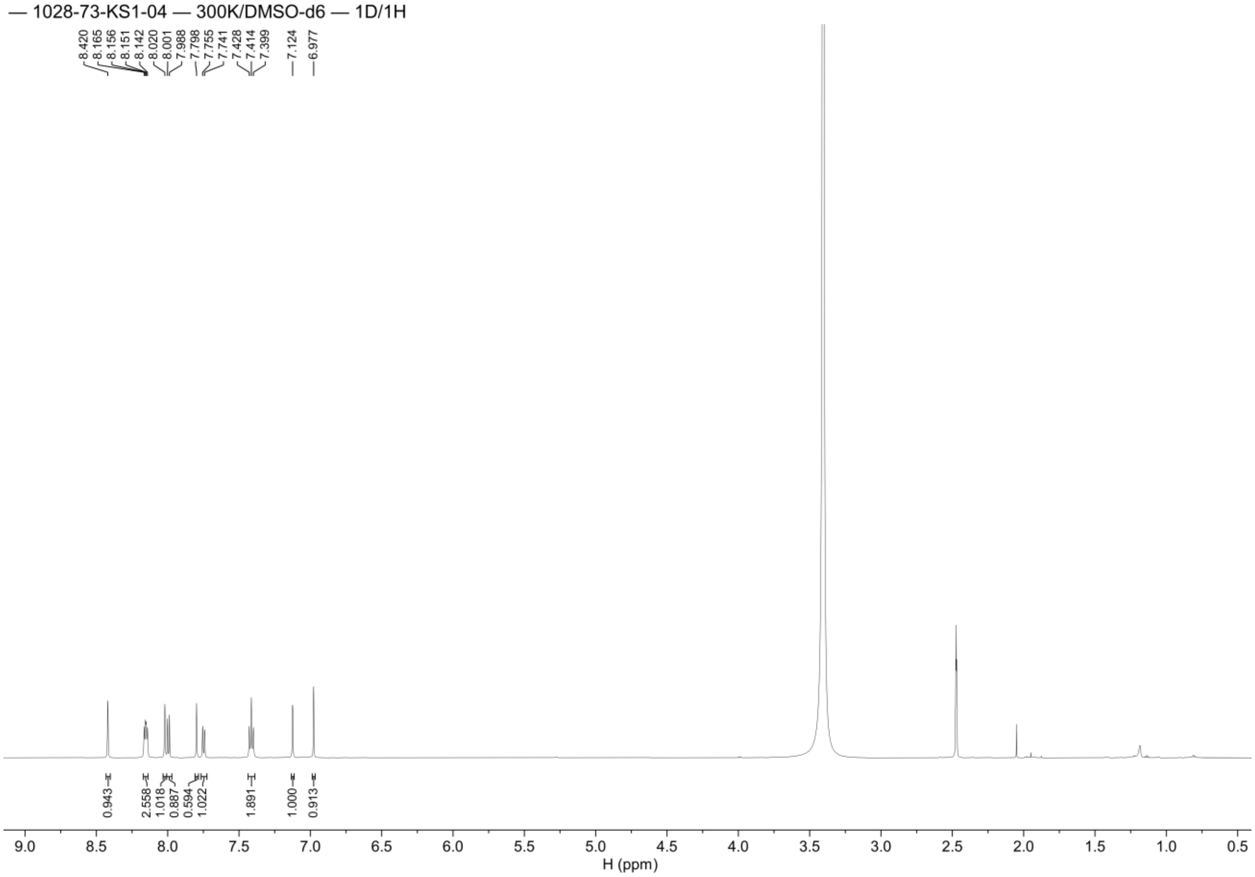

# $^{1}$H NMR of Compound 15

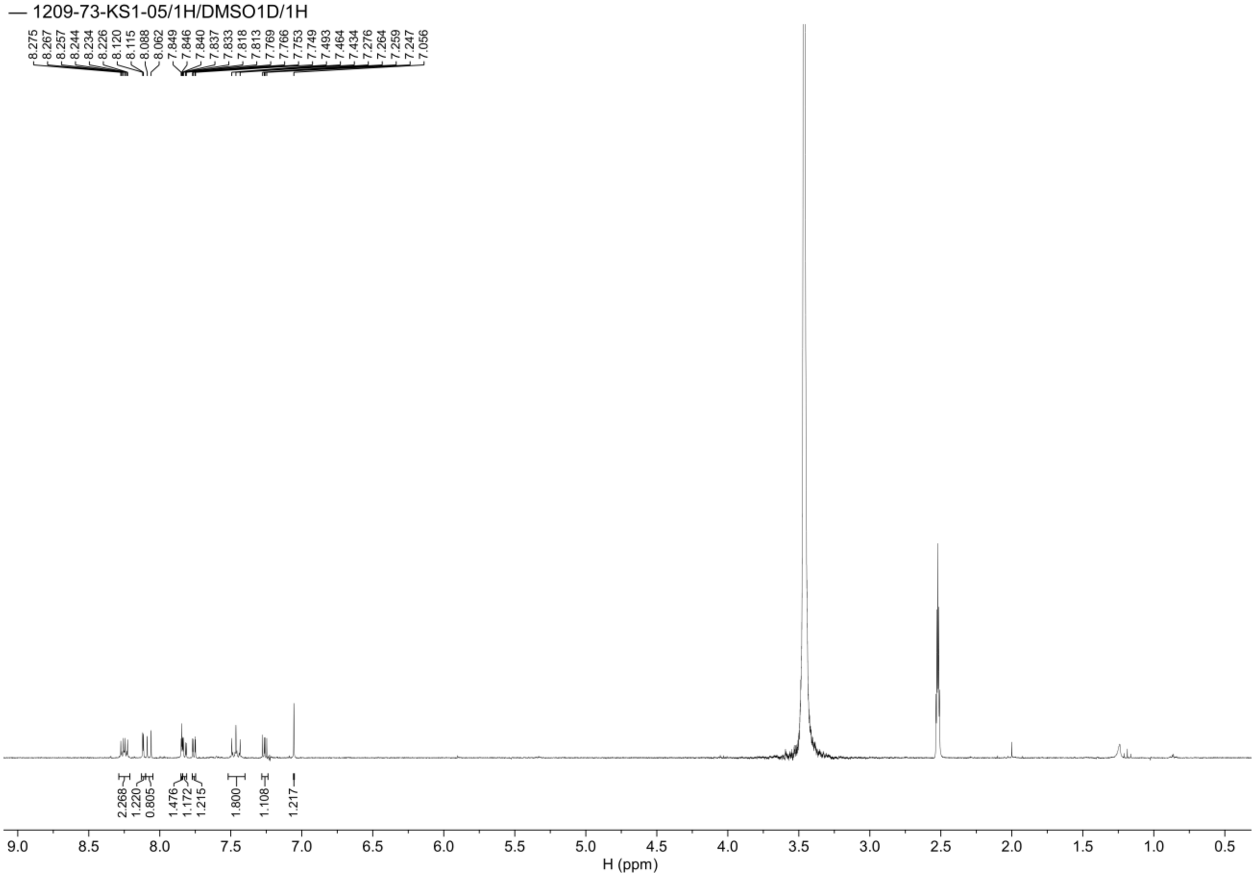

# $^{1}$H NMR of Compound 16

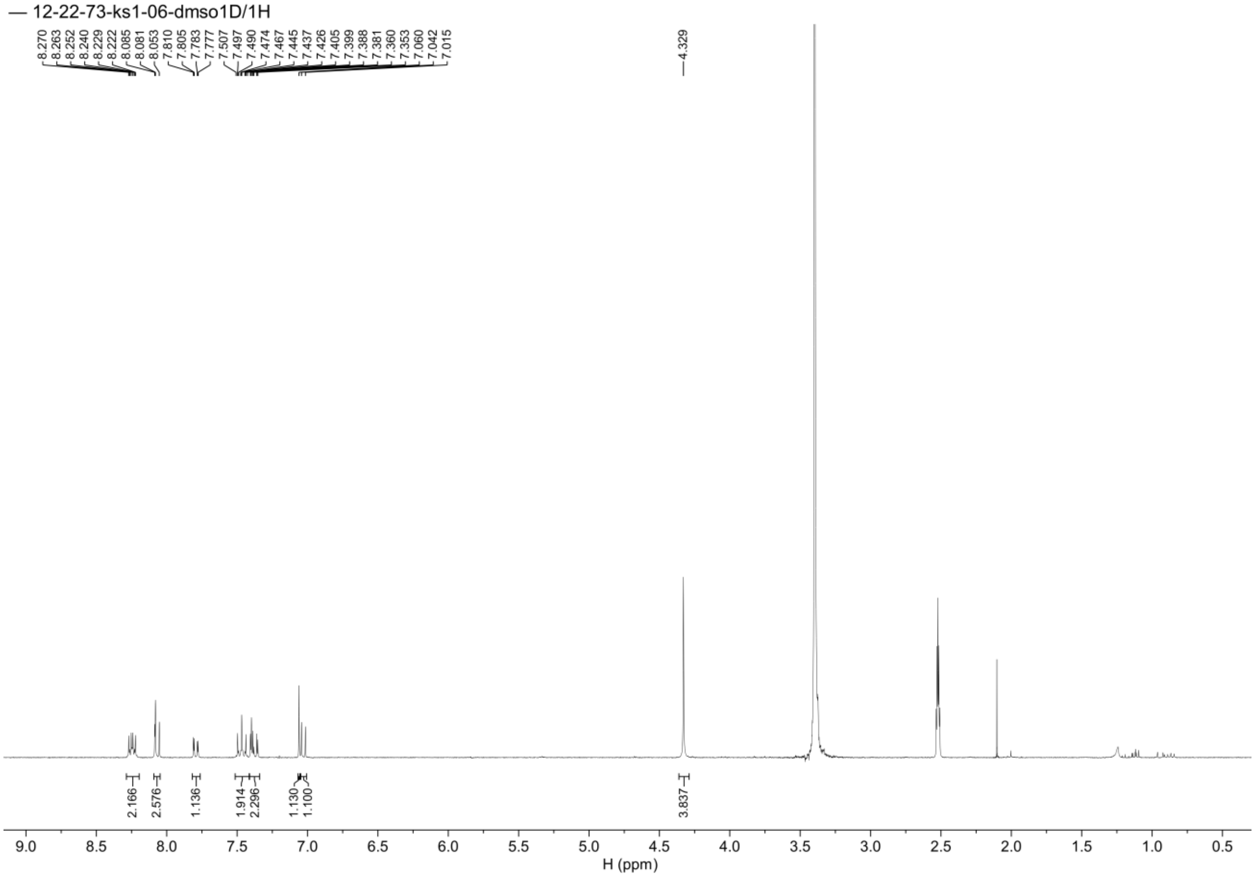

# $^{1}$H NMR of Compound 17

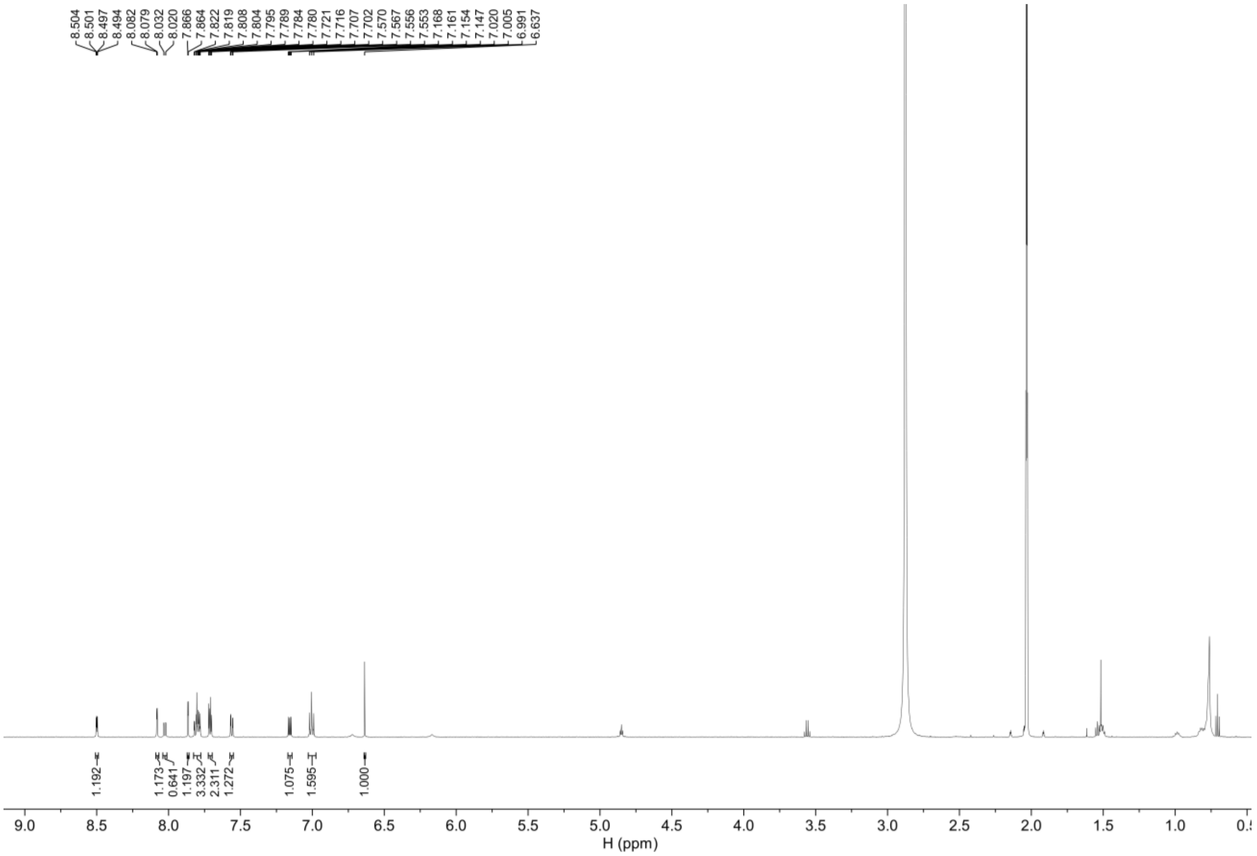

# $^1$H NMR of Compound 18

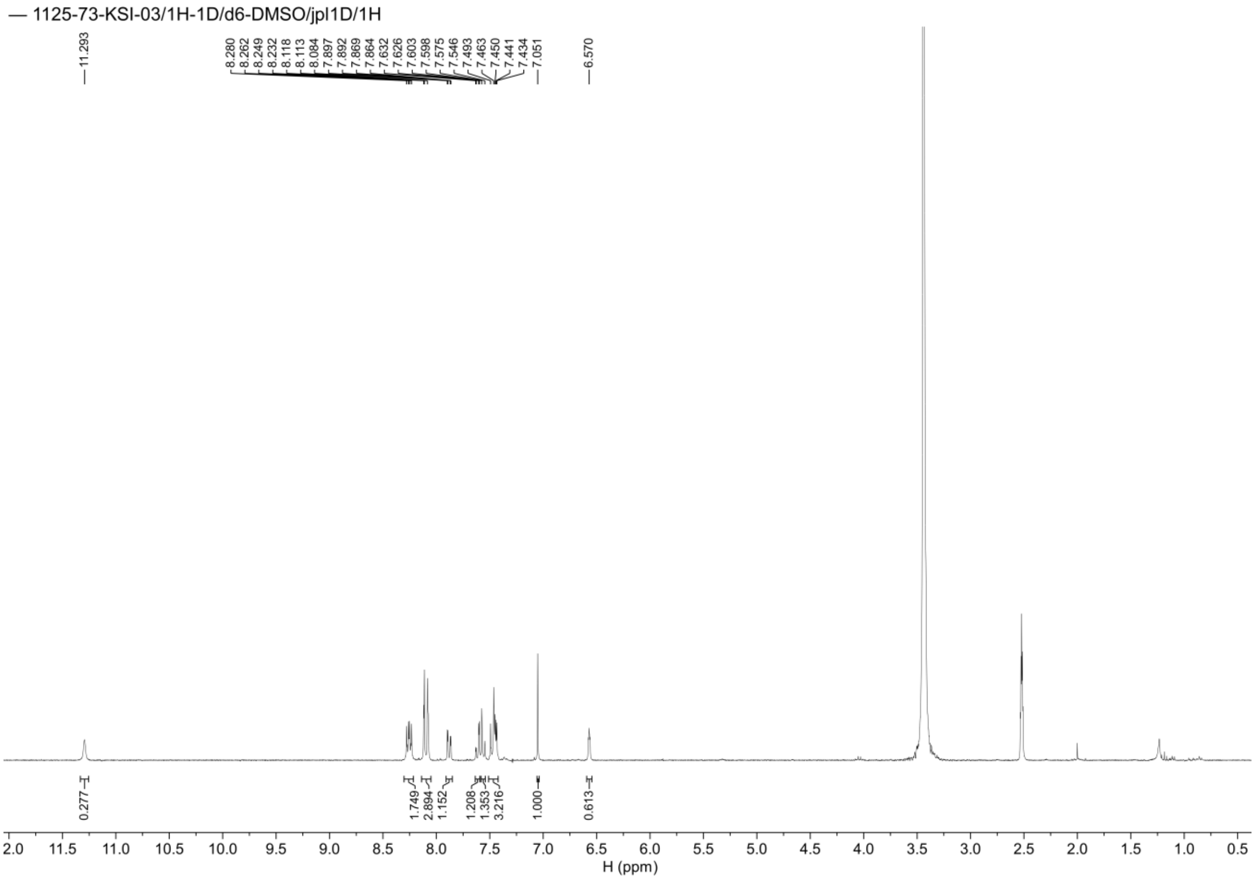

# $^{1}$H NMR of Compound 19

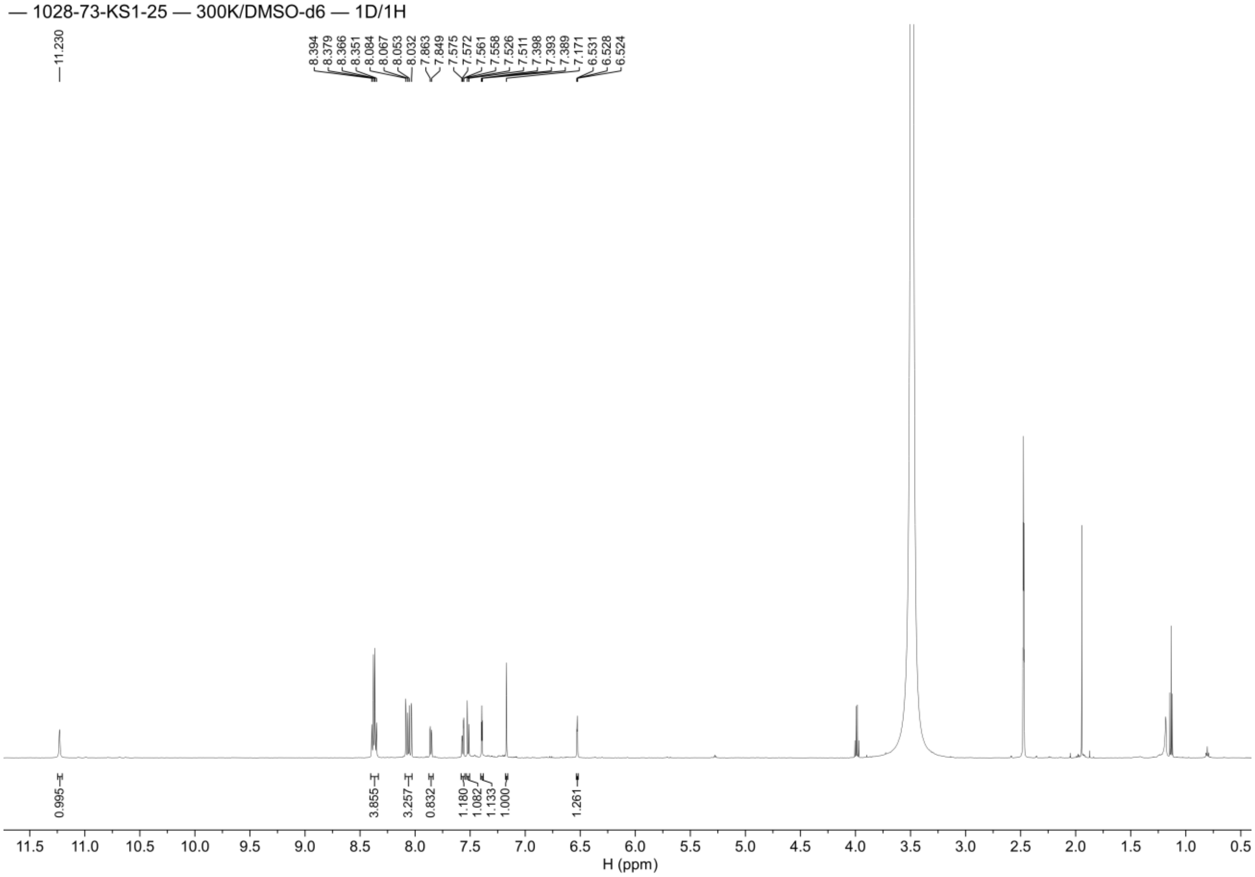

# $^{1}$H NMR of Compound 20

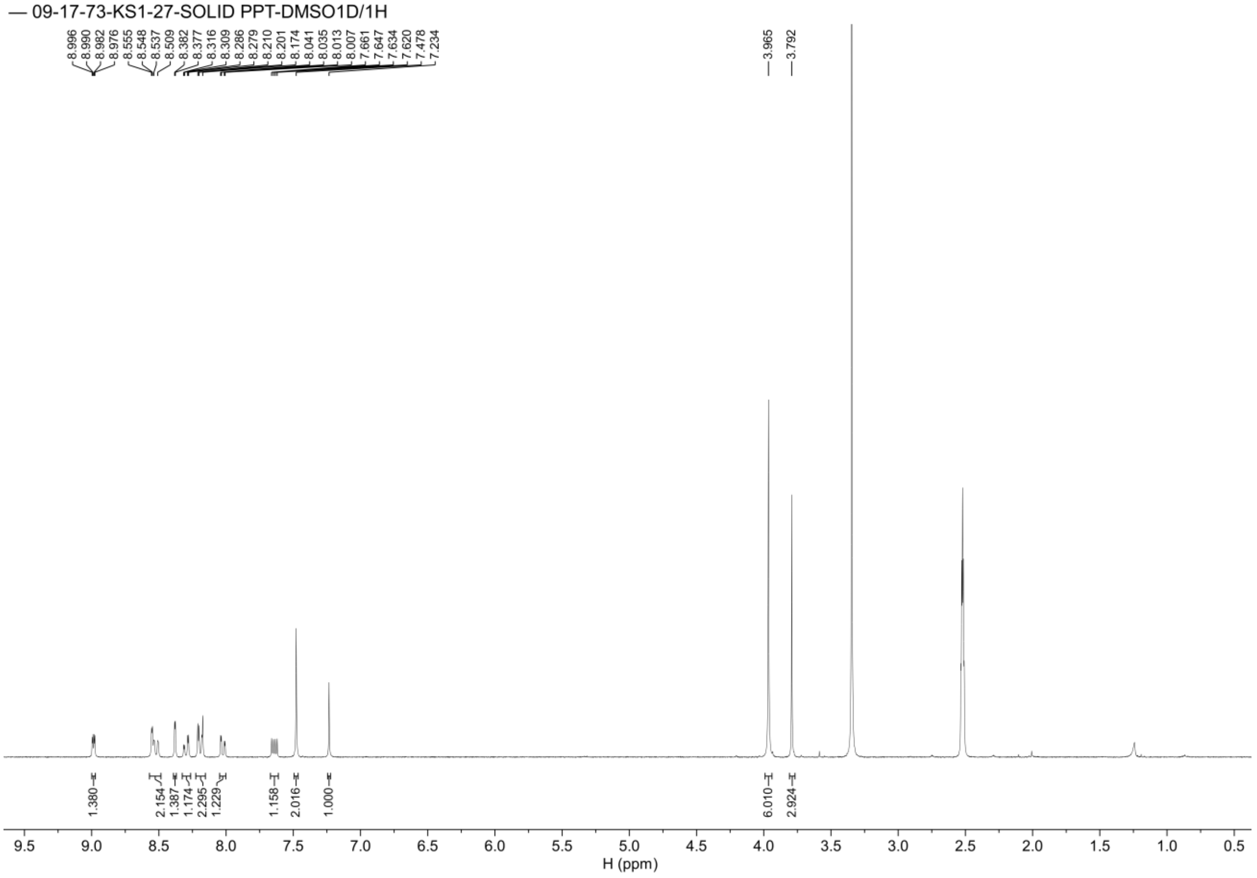

# $^{1}$H NMR of Compound 21

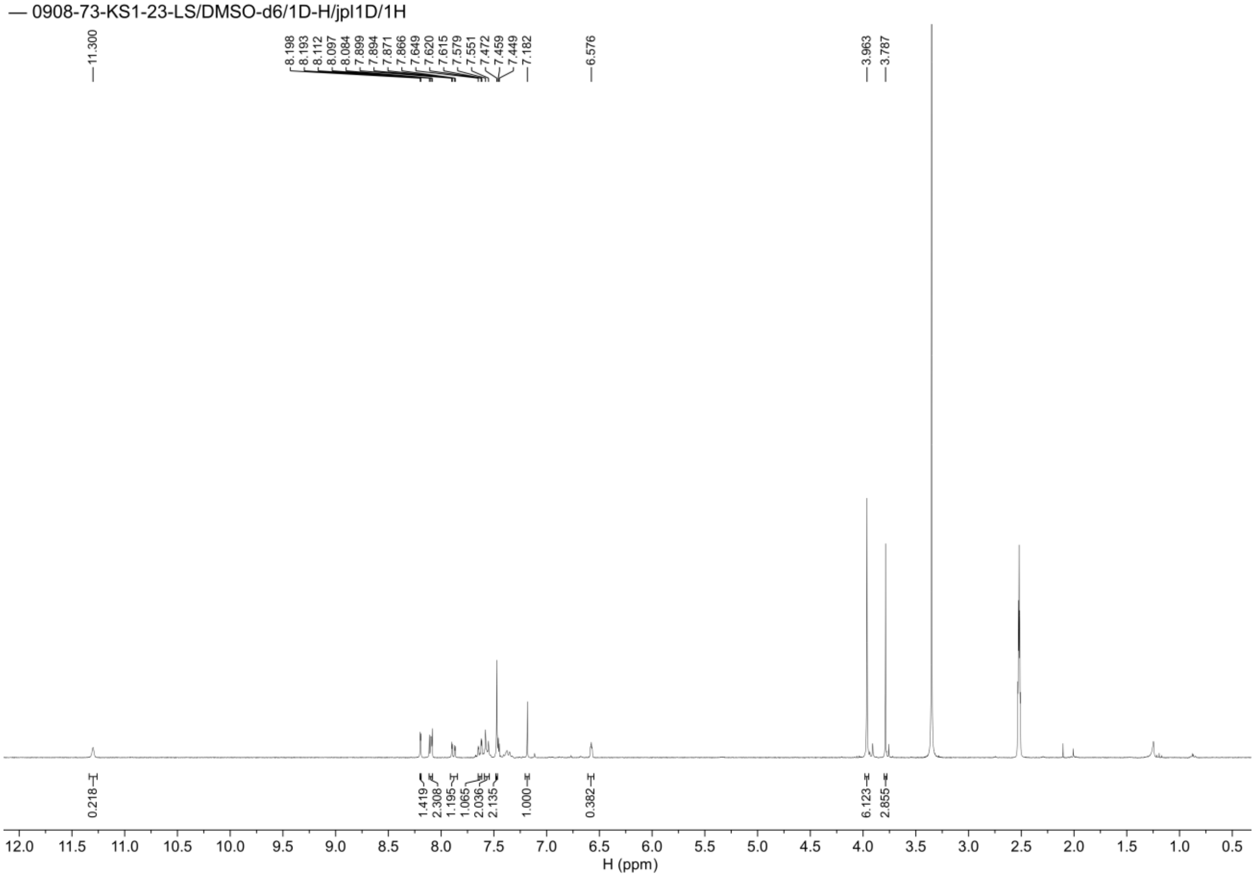

## $^{13}$C NMR of Compound 1

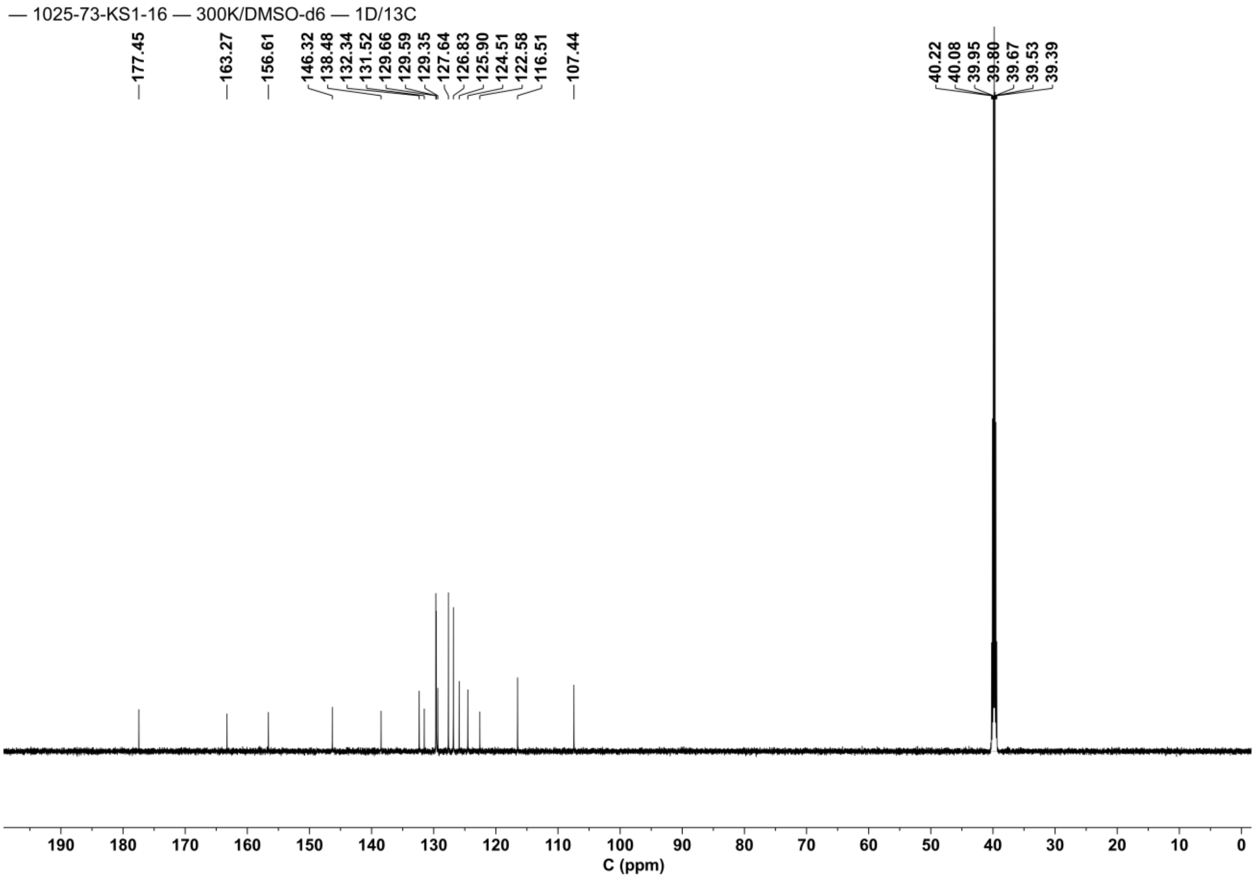

## $^{13}C$ NMR of Compound 2

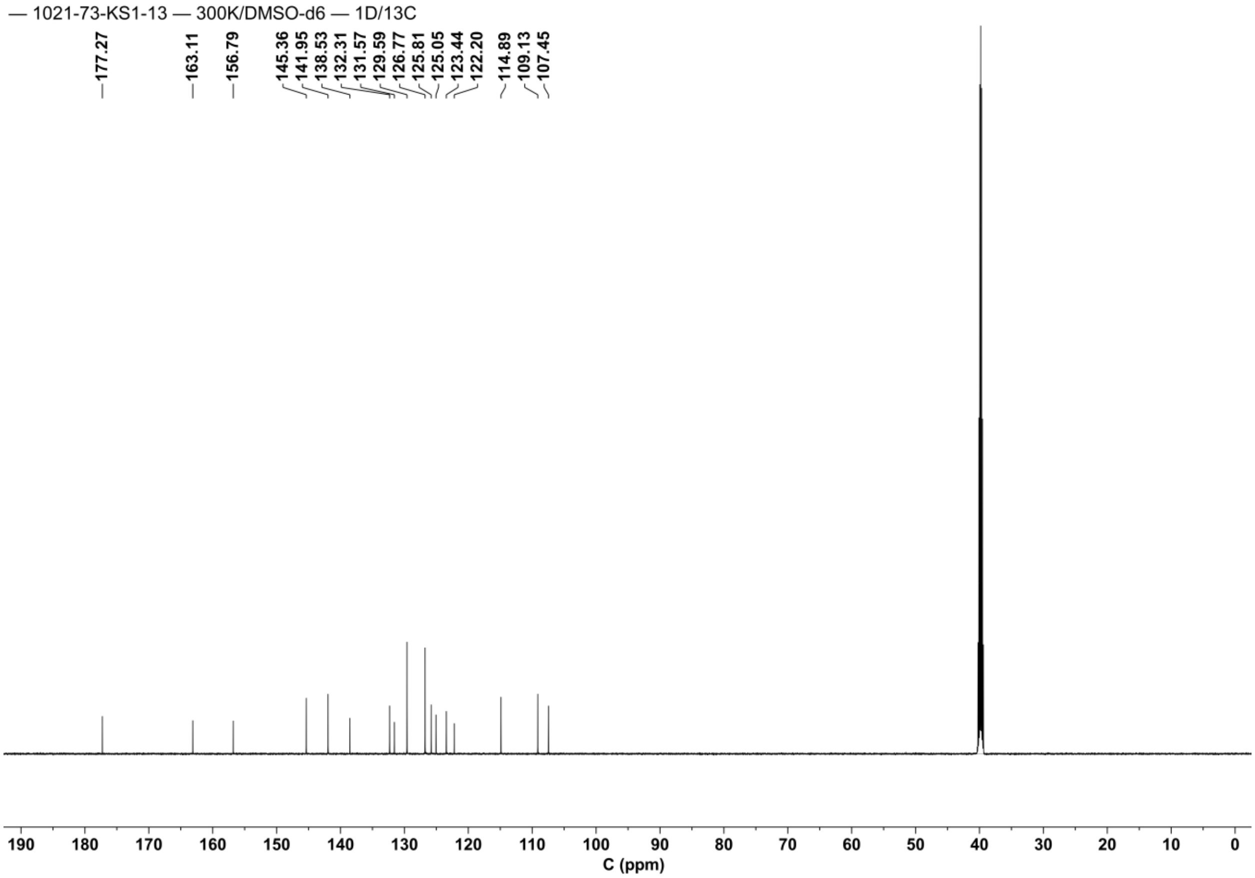

# $^{13}$C NMR of Compound 3

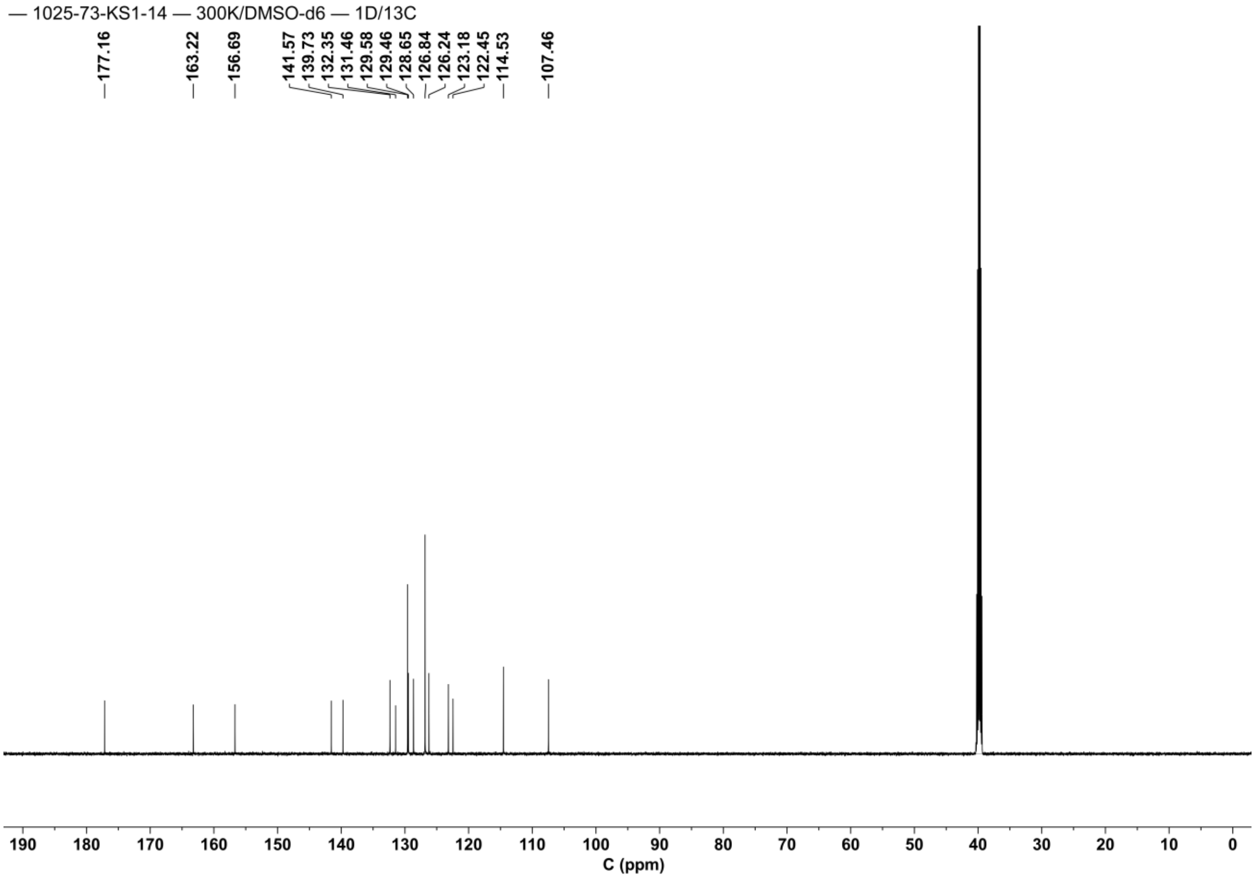

# $^{13}$C NMR of Compound 4

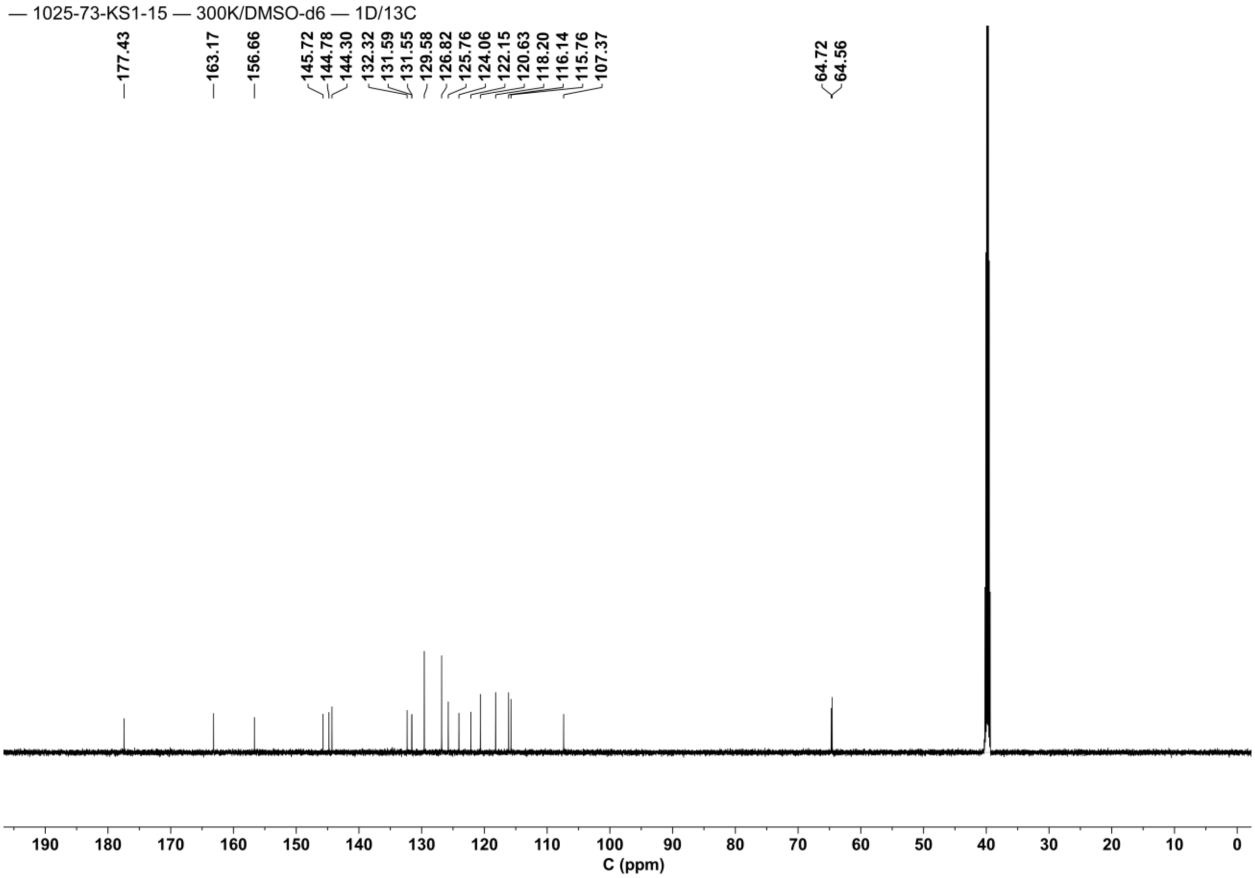

# $^{13}$C NMR of Compound 5

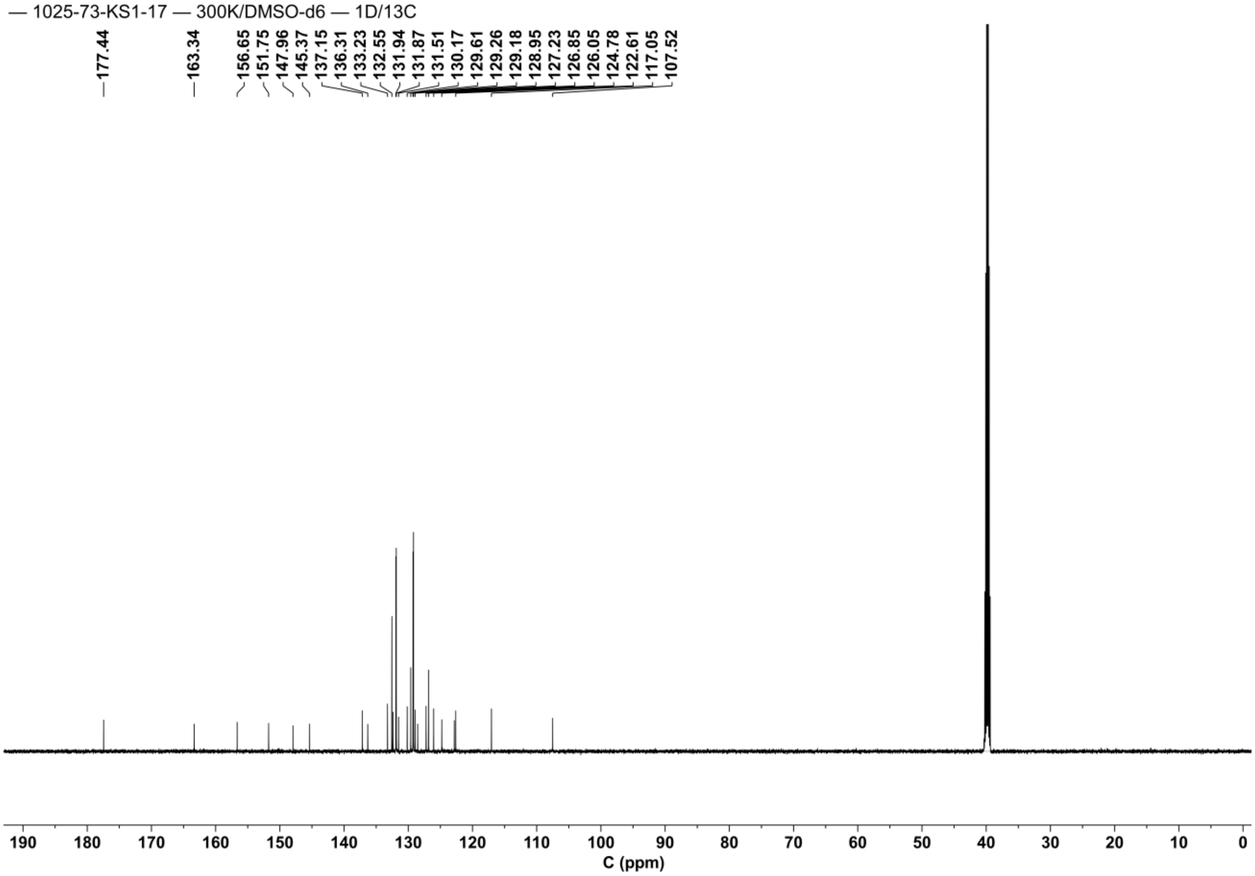

# $^{13}$C NMR of Compound 6

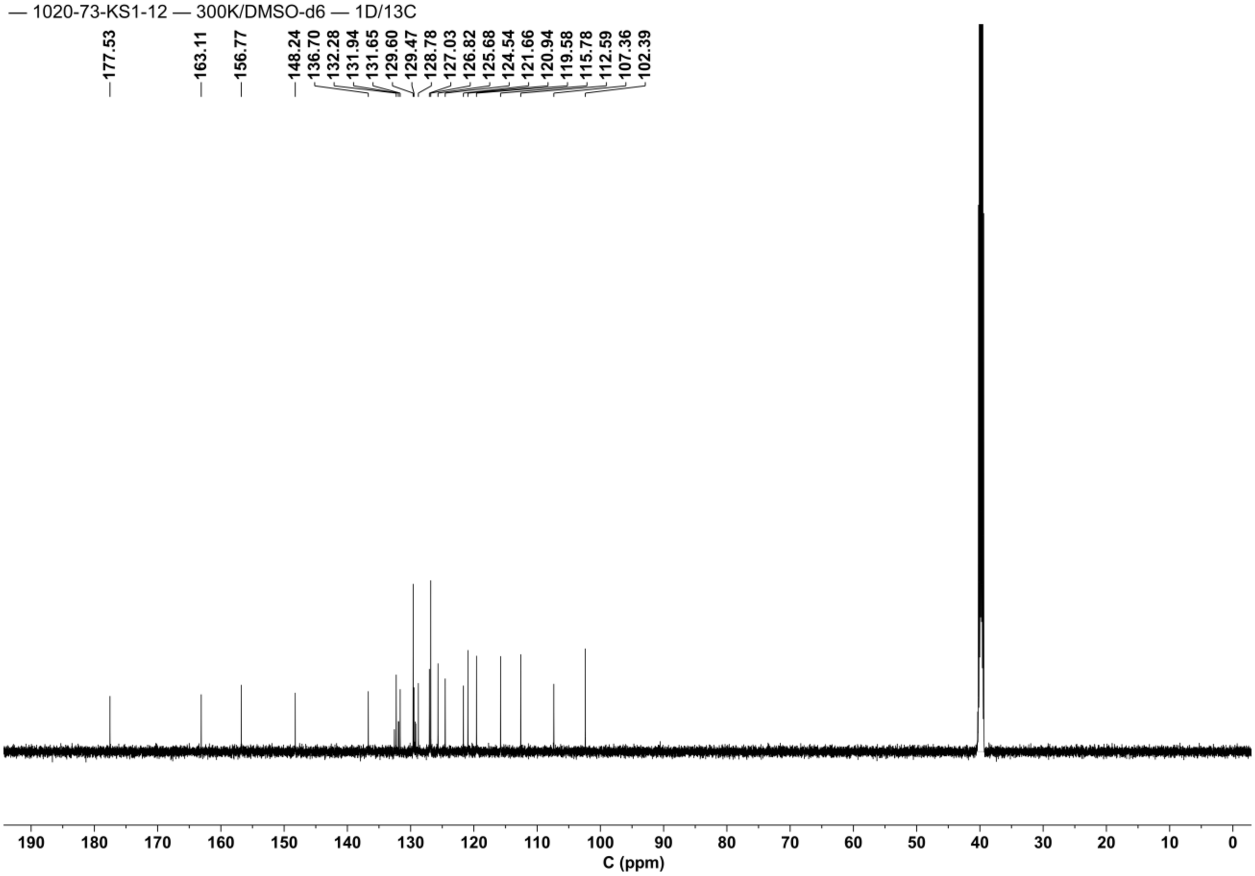

# $^{13}$C NMR of Compound 7

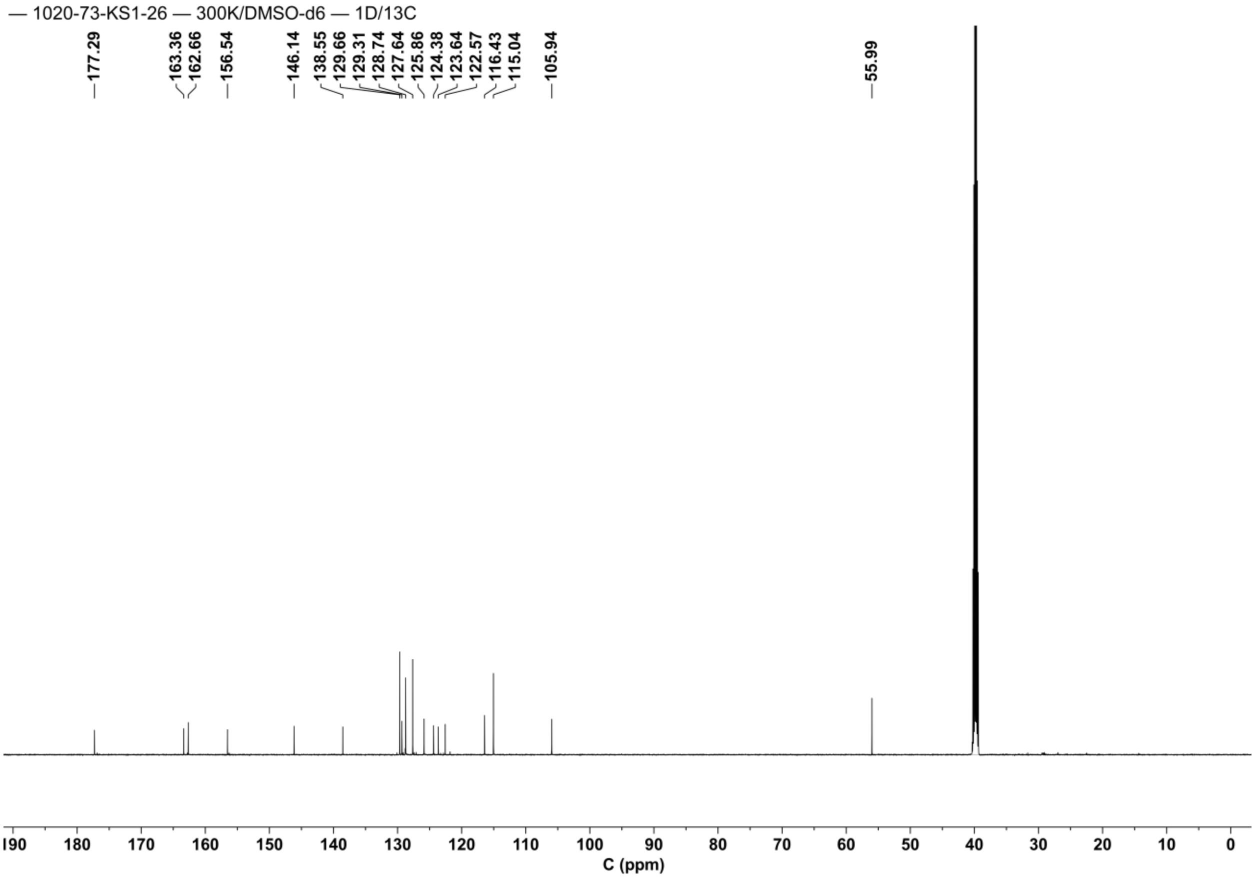

# $^{13}C$ NMR of Compound 8

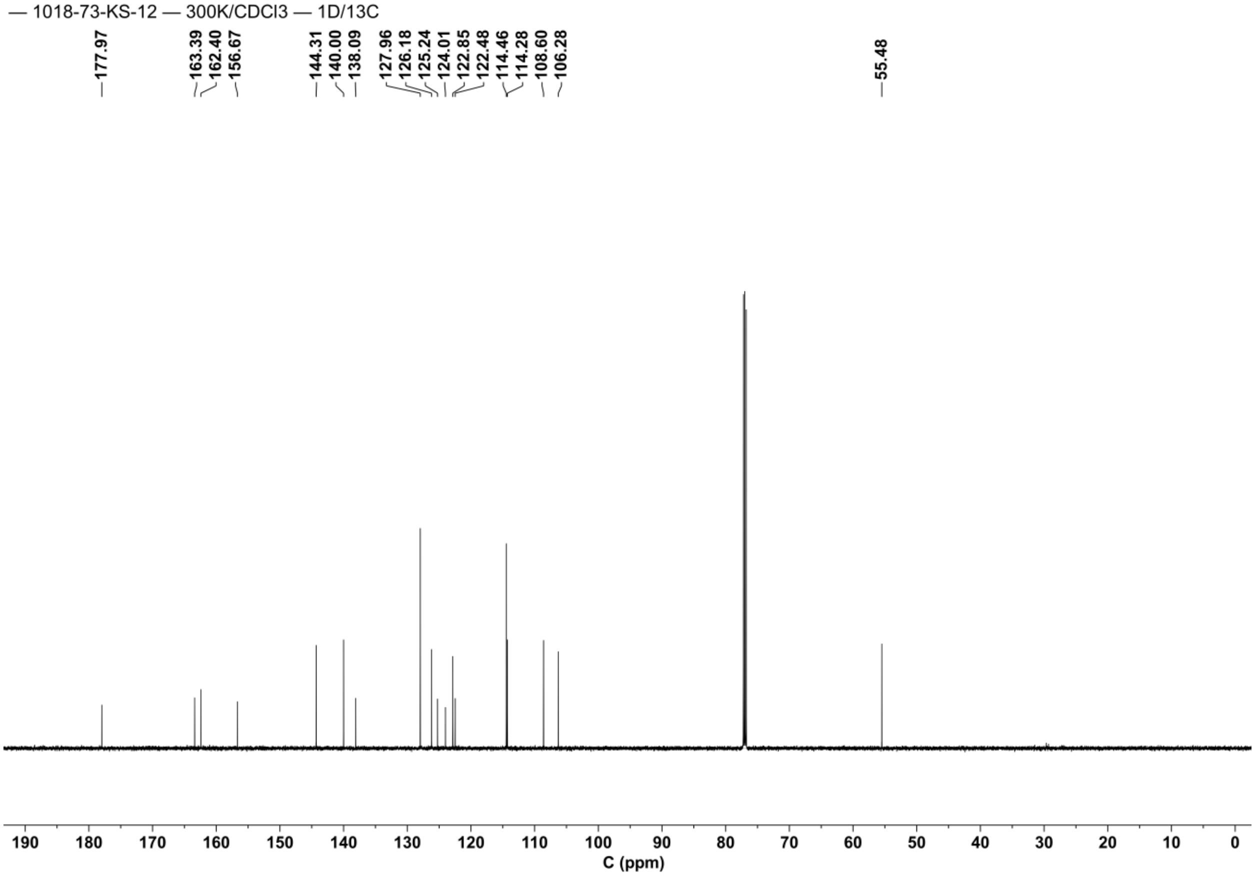

# $^{13}C$ NMR of Compound 9

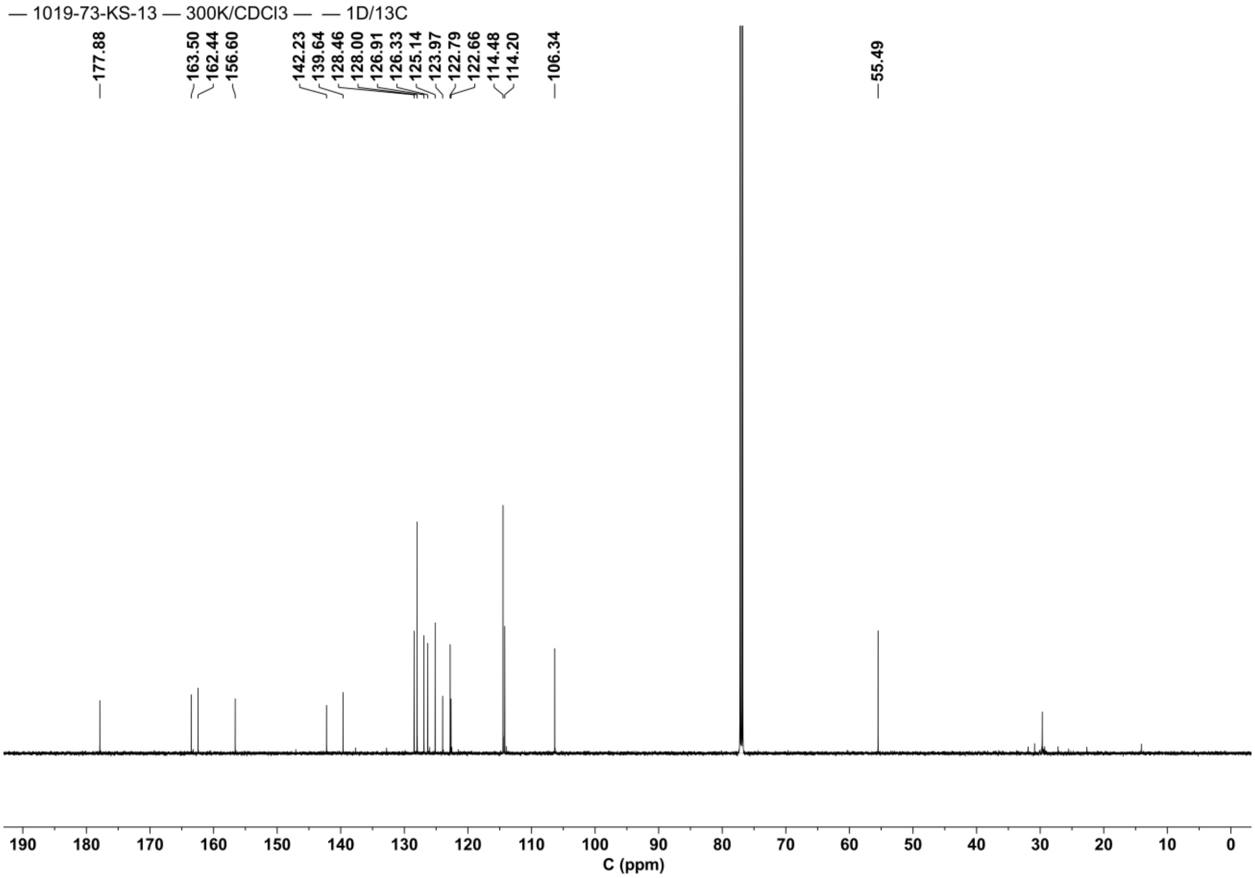

# $^{13}C$ NMR of Compound 10

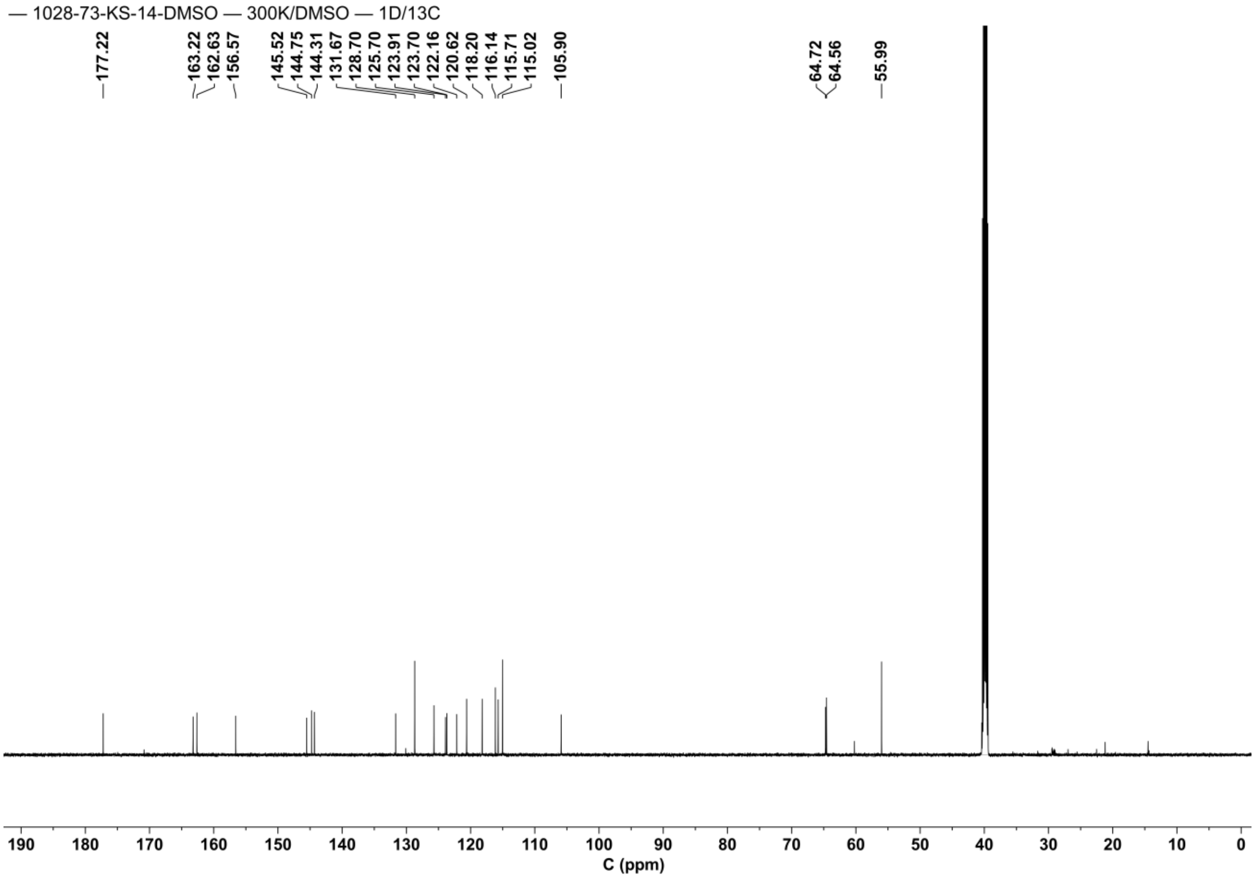

# $^{13}$C NMR of Compound 11

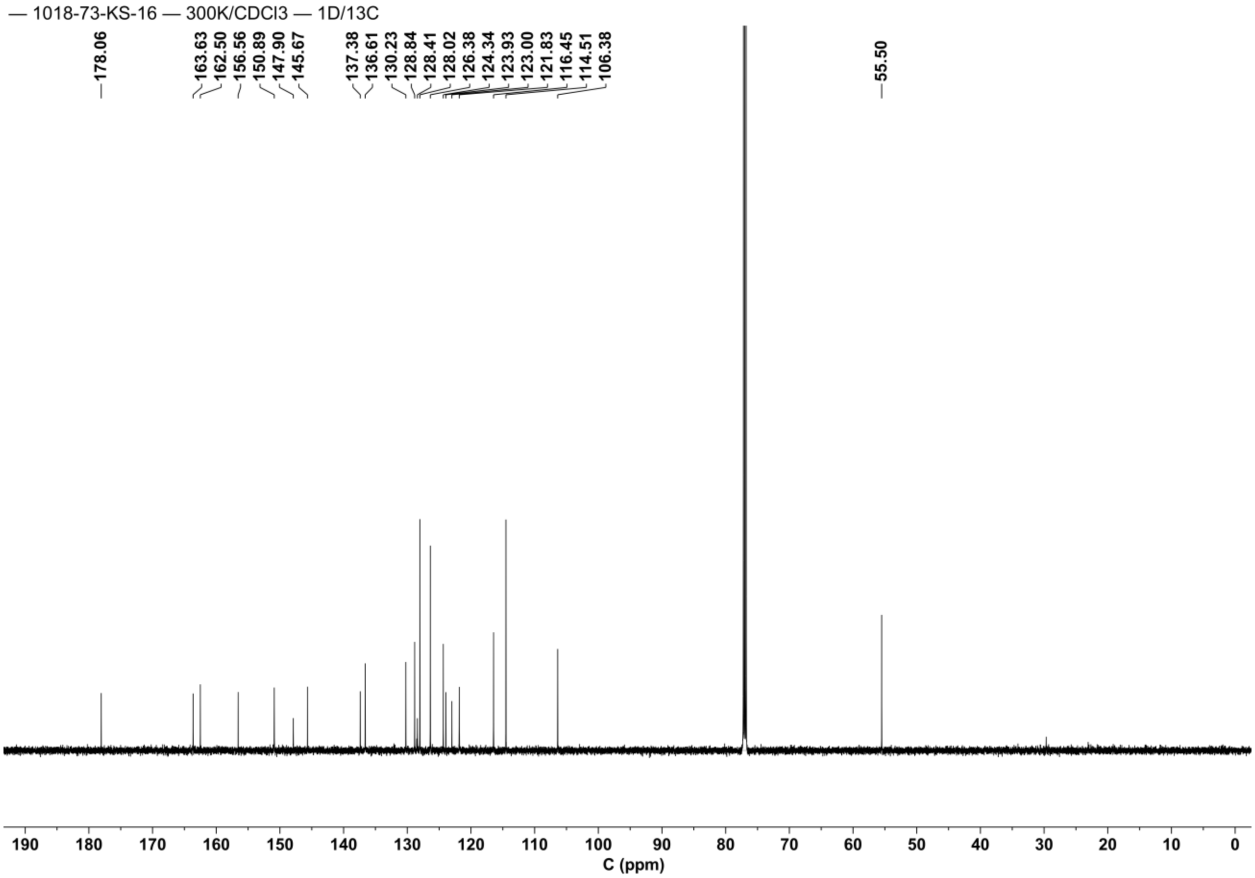

# $^{13}$C NMR of Compound 12

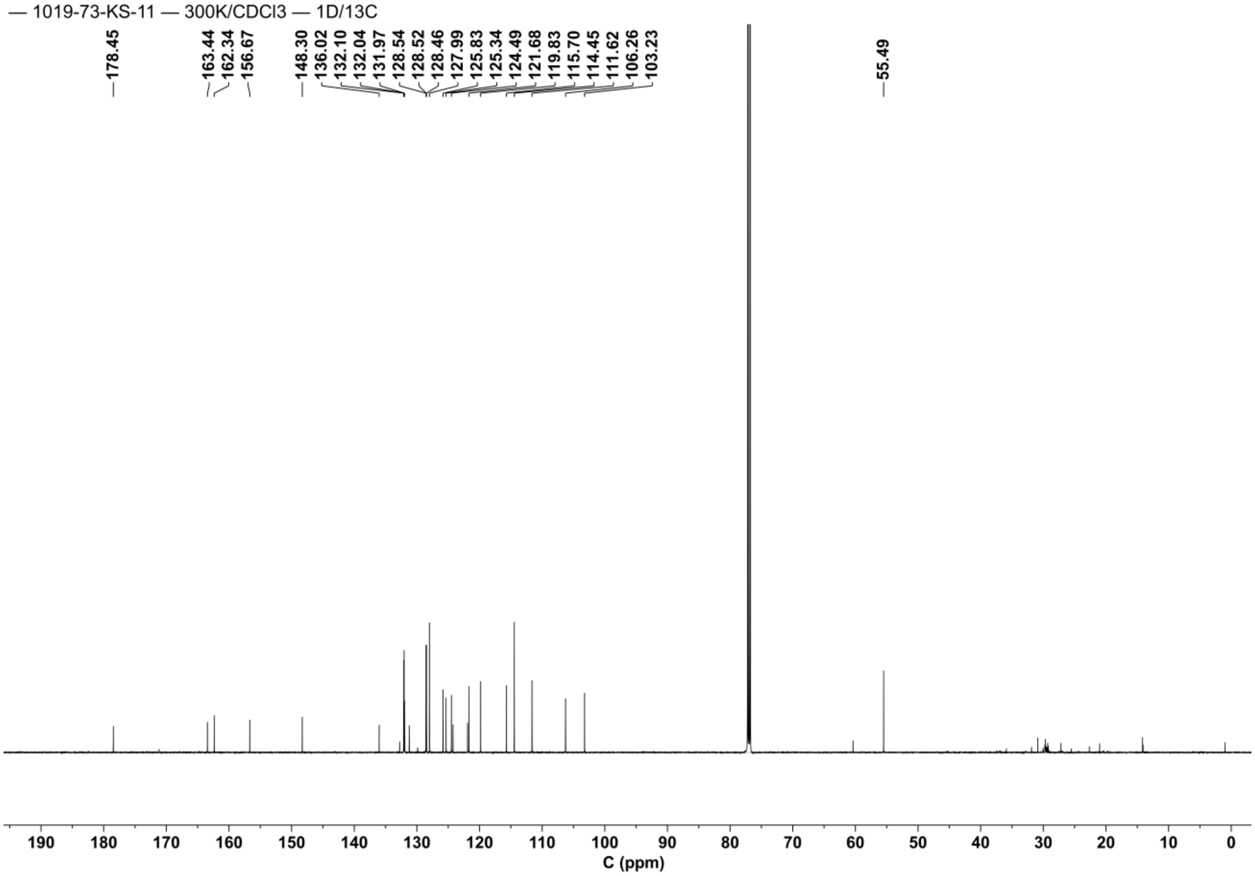

# $^{13}$C NMR of Compound 13

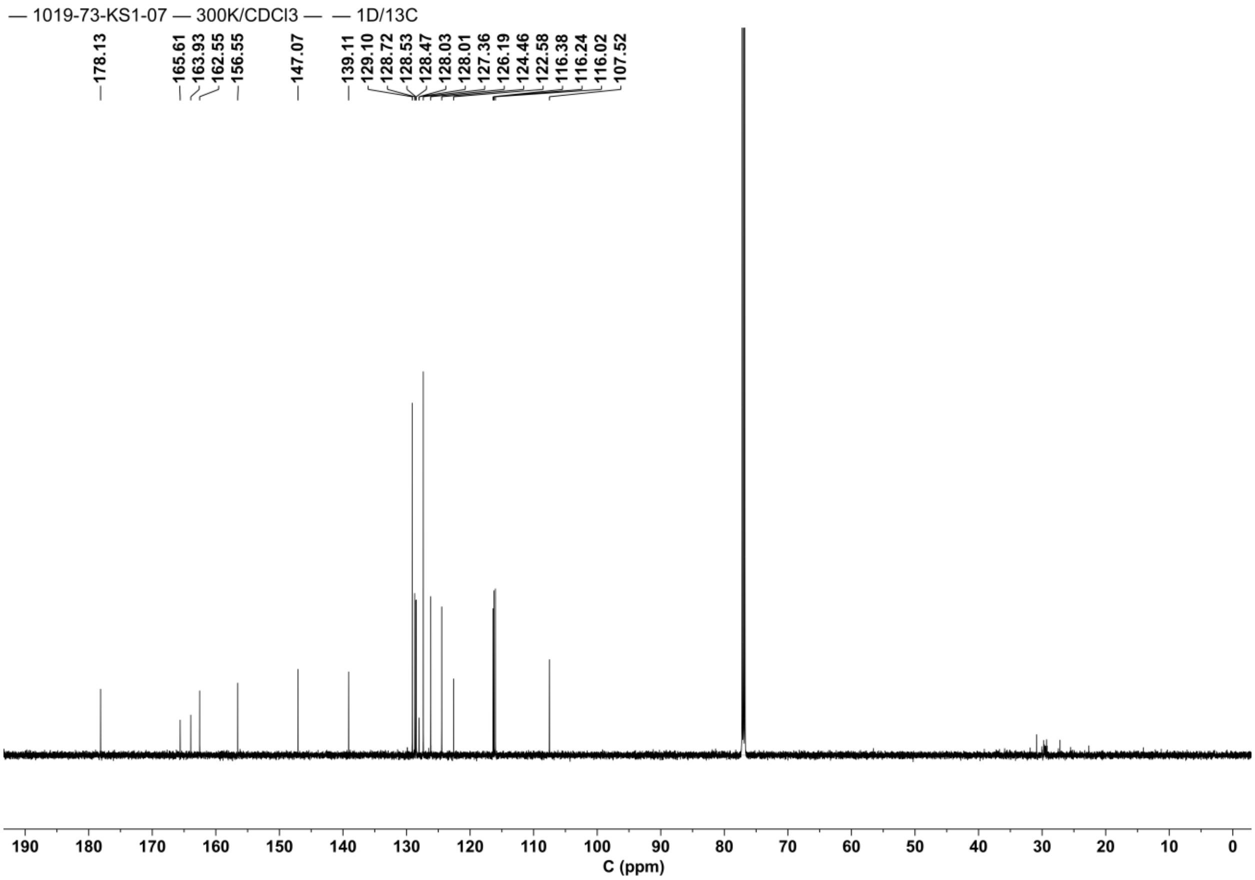

# $^{13}C$ NMR of Compound 14

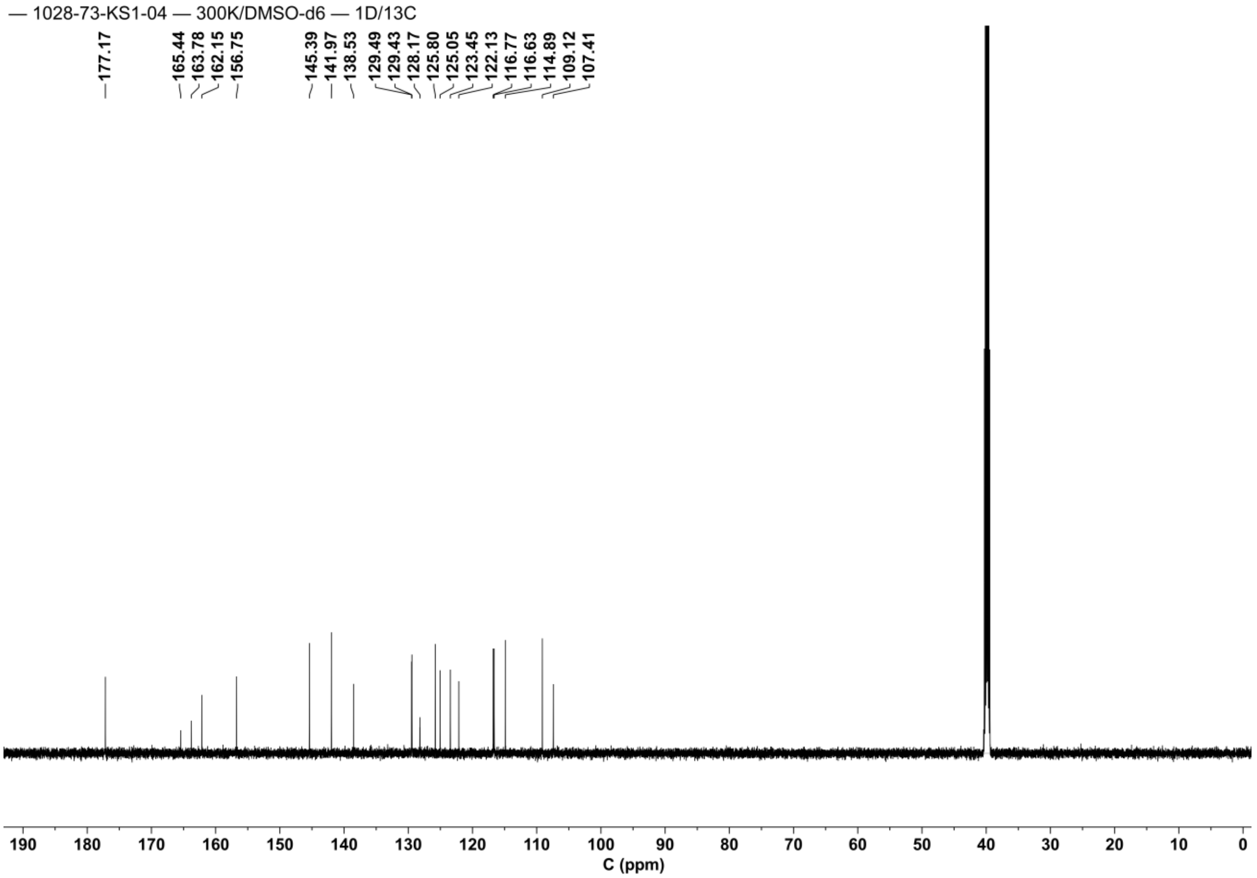

# $^{13}$C NMR of Compound 15

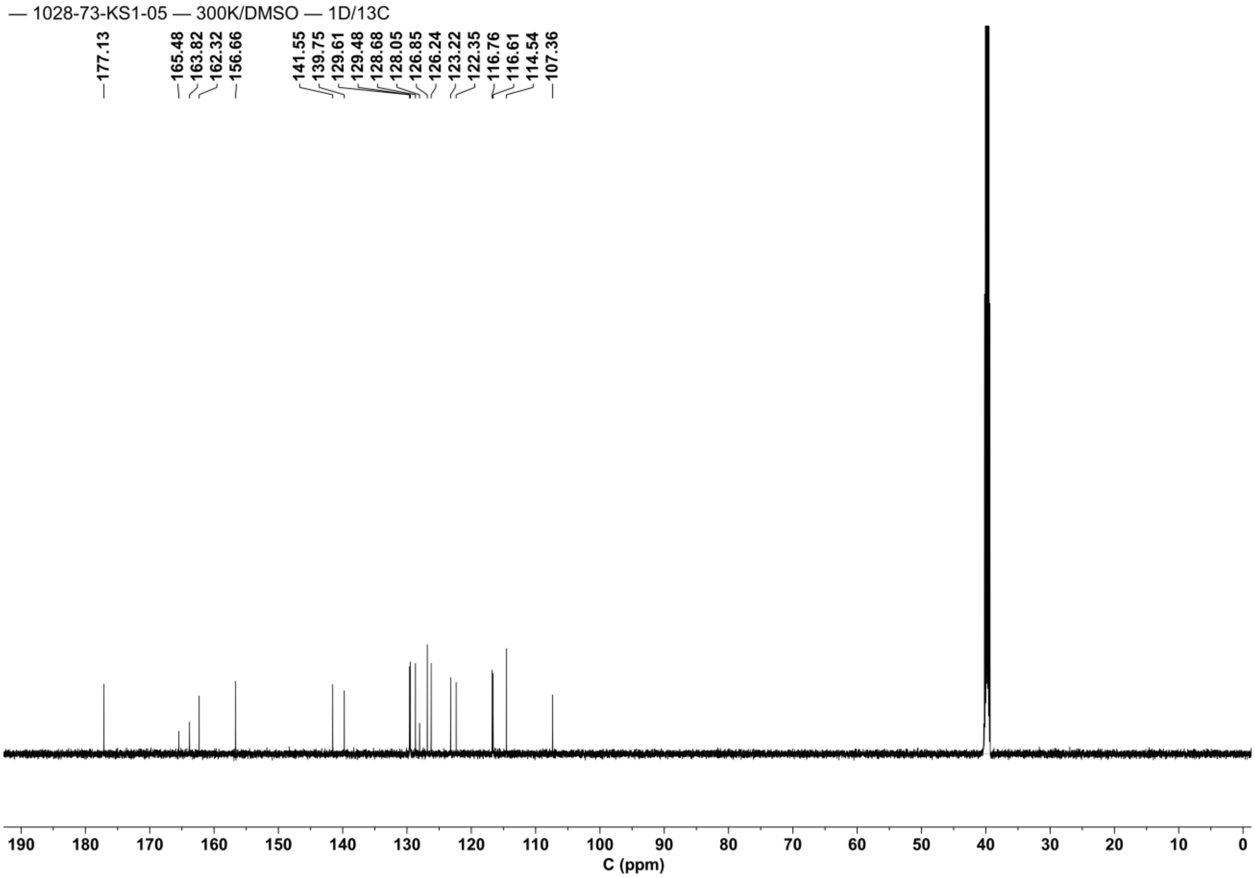

# $^{13}C$ NMR of Compound 16

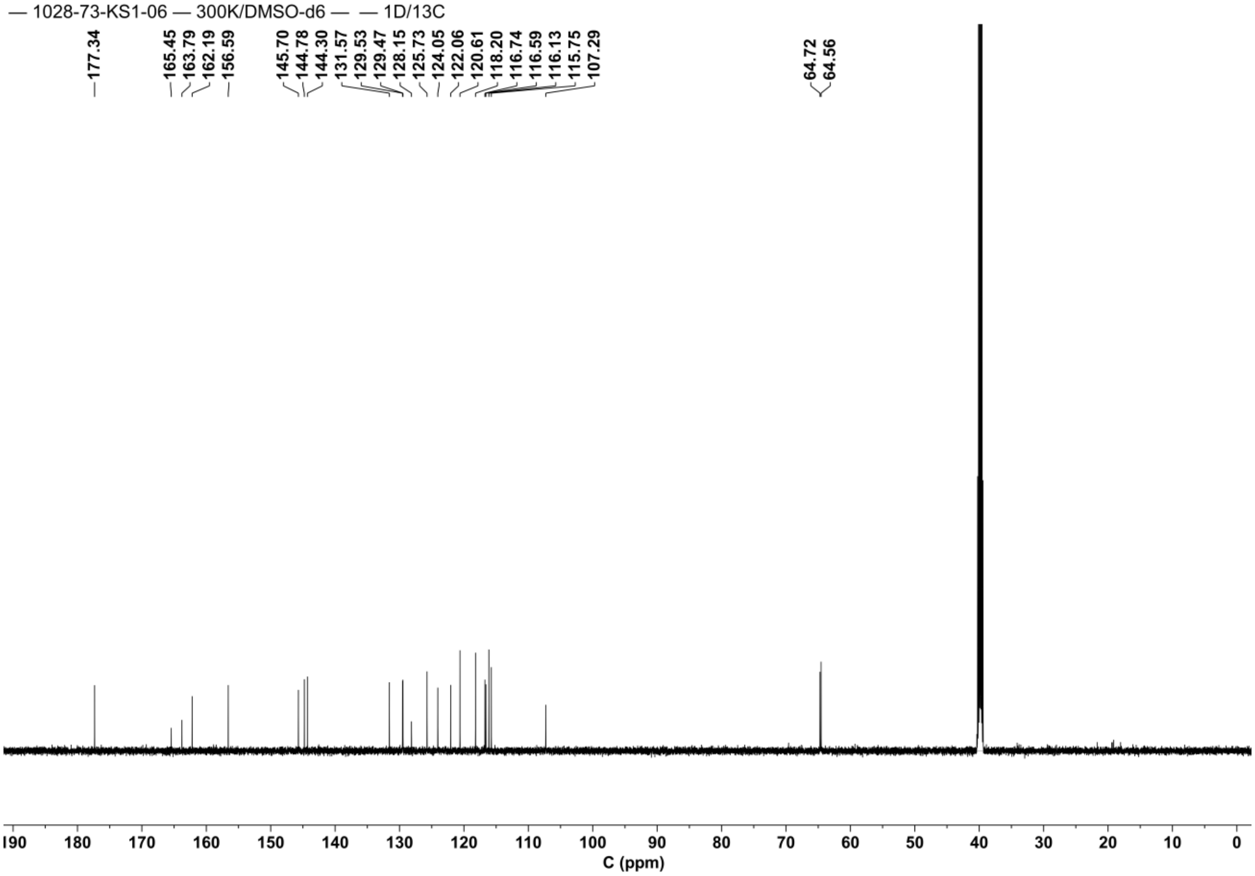

## $^{13}C$ NMR of Compound 17

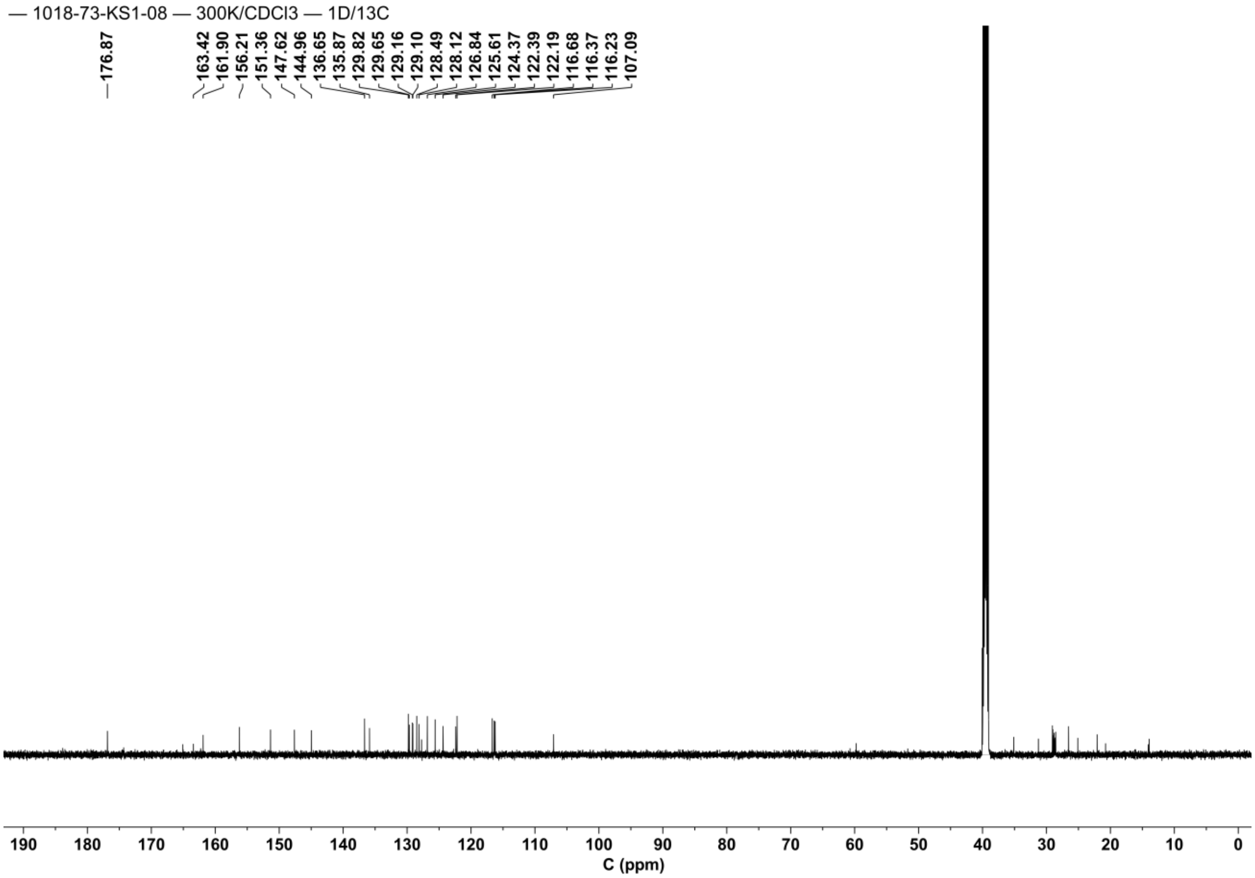

## $^{13}$C NMR of Compound 18

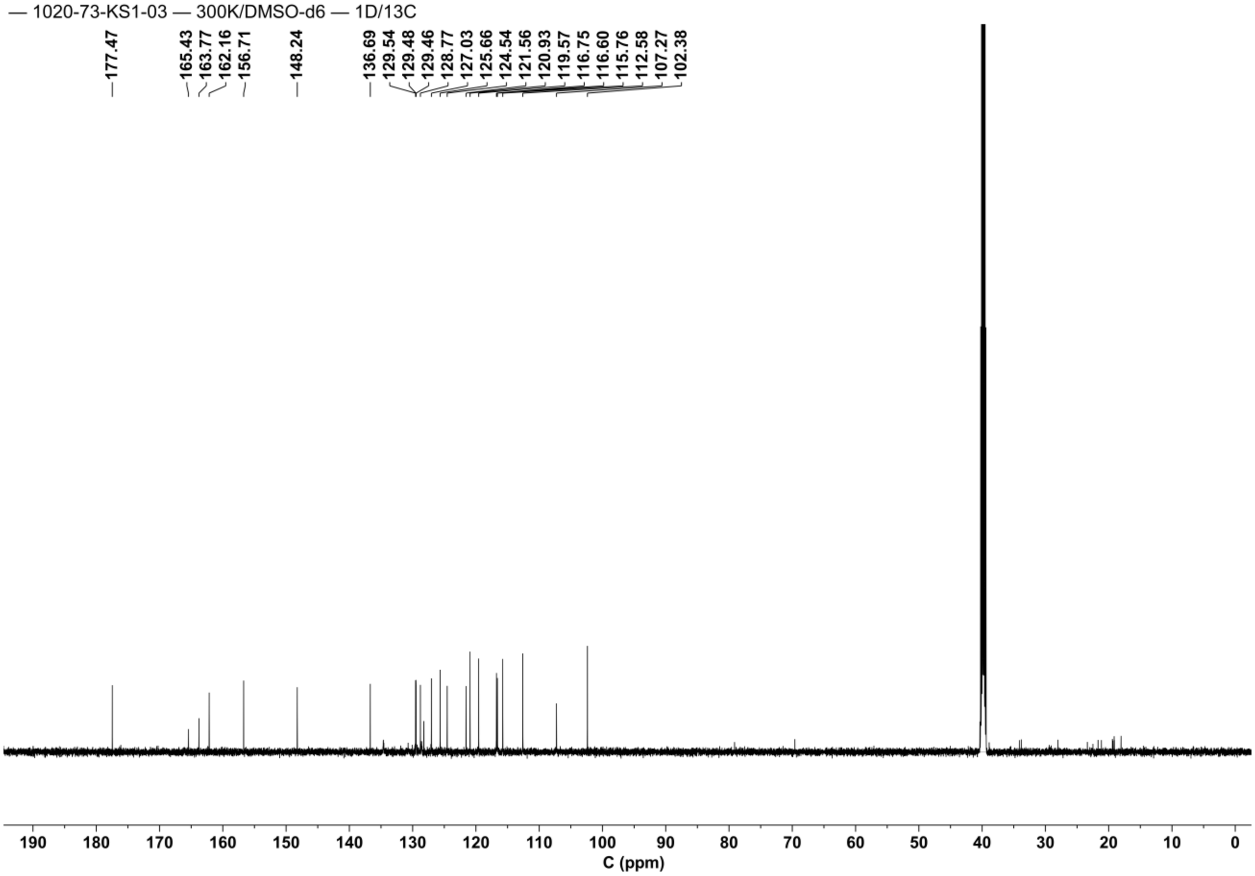

# $^{13}C$ NMR of Compound 19

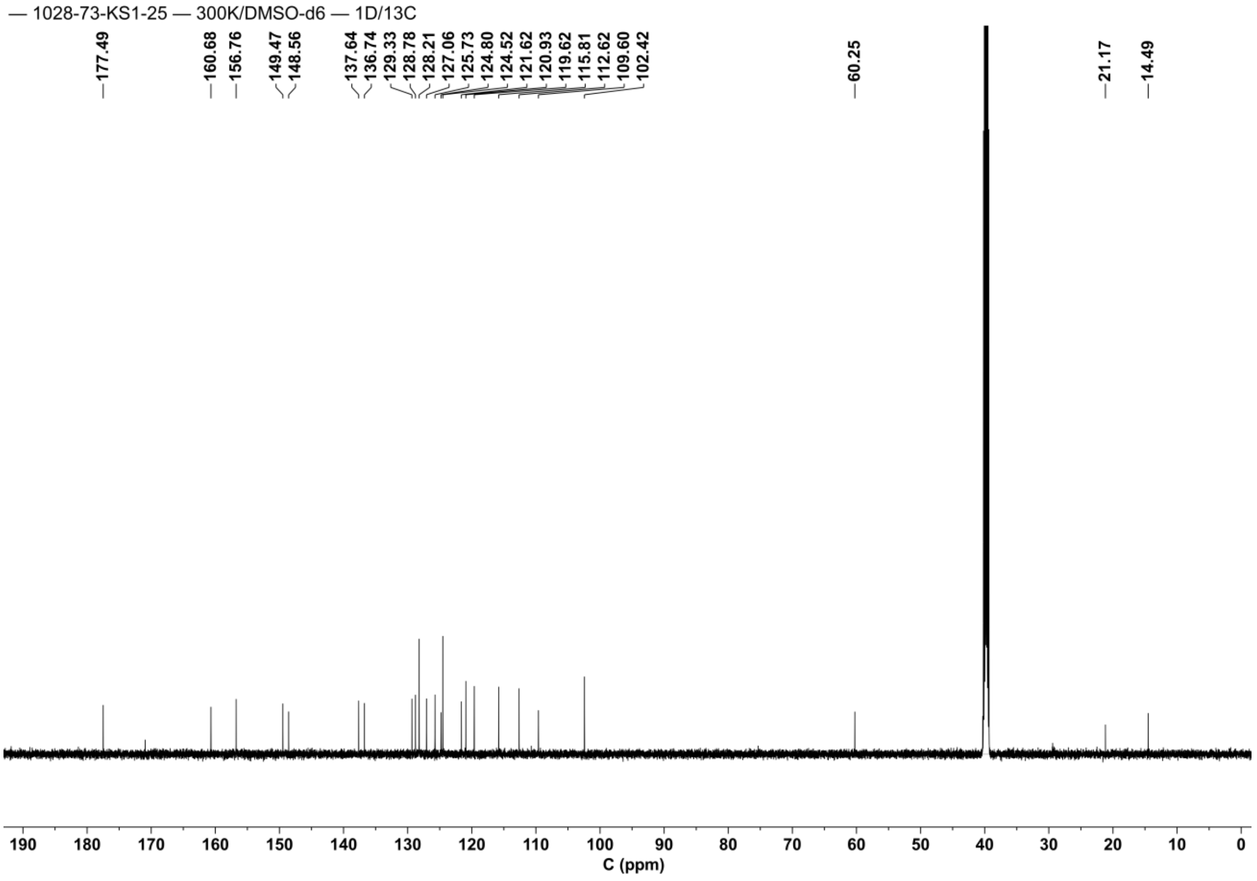

## $^{13}C$ NMR of Compound 20

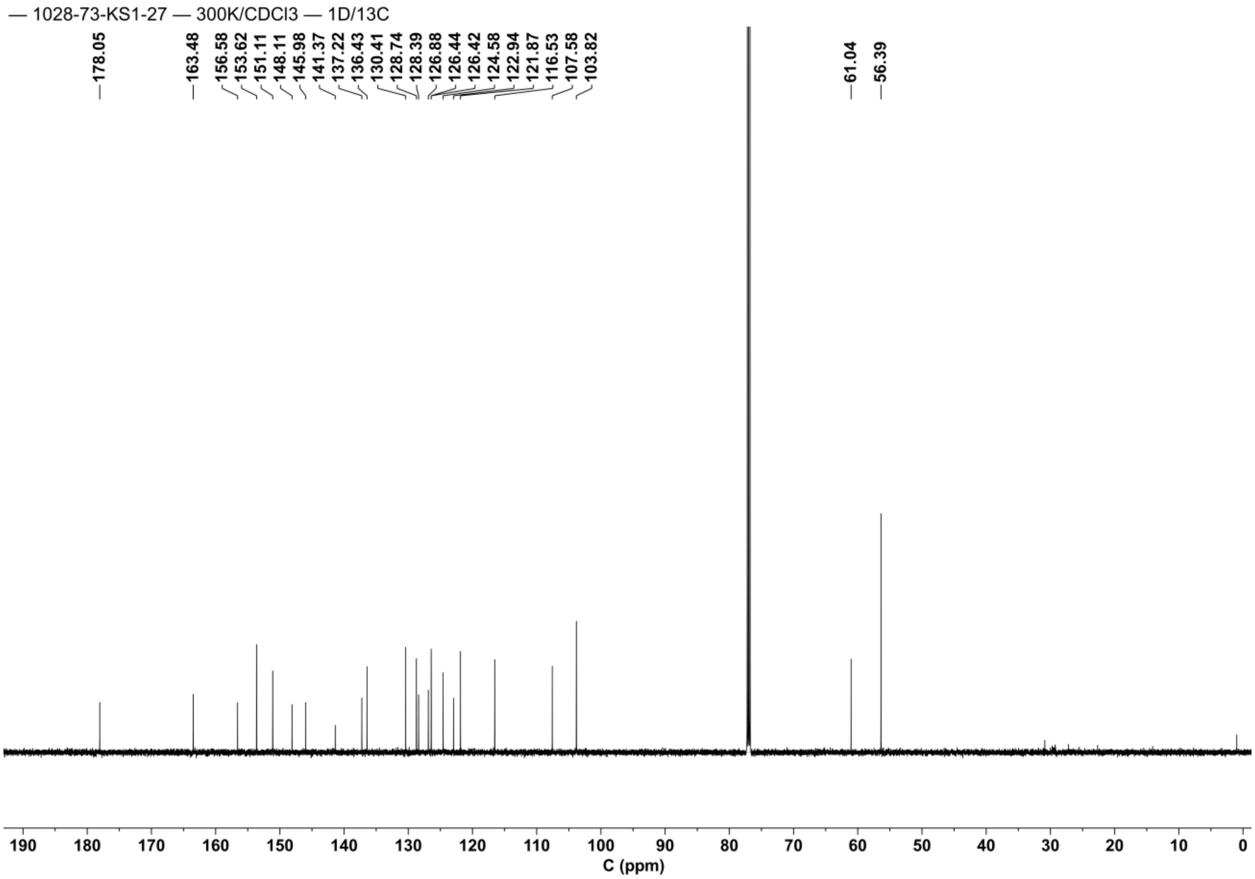

# $^{13}$C NMR of Compound 21

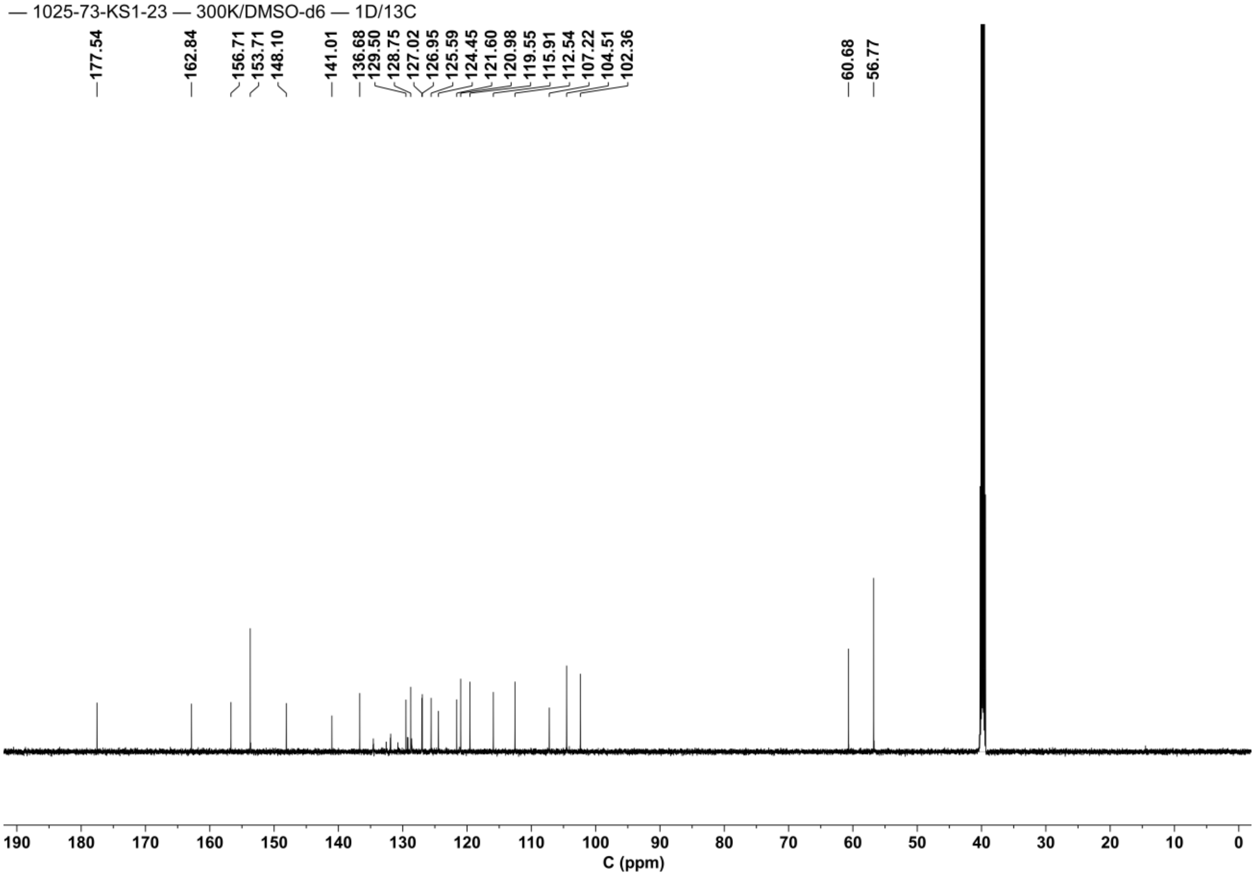

# HPLC Purity of Compound 1

SHIMADZU LabSolutions **Analysis Report**

## <Sample Information>

| | | | |
|---|---|---|---|
| Sample Name | : 73-KS1-16 | | |
| Sample ID | : | | |
| Data Filename | : 20221017_73-KS1-16_007.lcd | | |
| Method Filename | : NHRI purity test.lcm | | |
| Batch Filename | : 101722_73KS1_1.lcb | | |
| Vial # | : 1-16 | Sample Type | : Unknown |
| Injection Volume | : 20 uL | | |
| Date Acquired | : 2022/10/18 05:25:32 | Acquired by | : System Administrator |
| Date Processed | : 2022/10/18 06:25:34 | Processed by | : System Administrator |

## <Chromatogram>

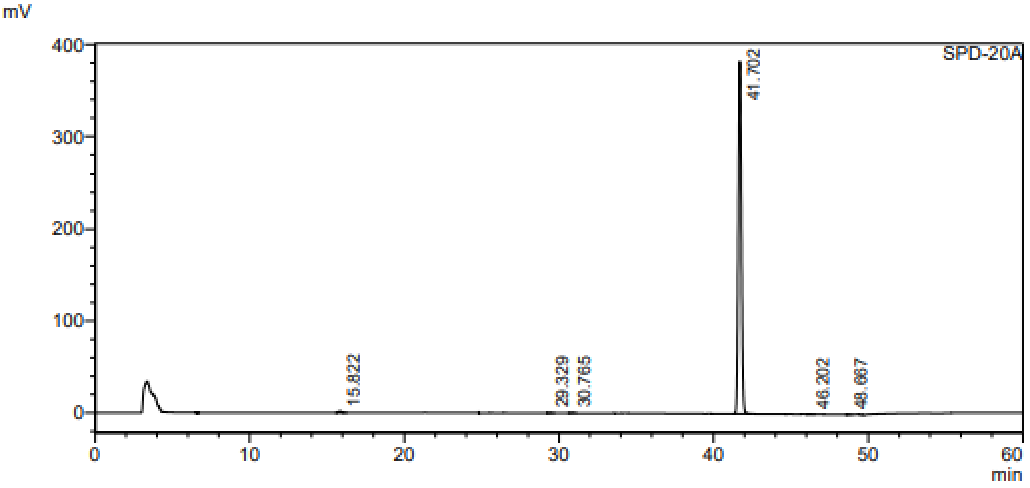


Peak Table

SPD-20A

| Peak# | Ret. Time | Area | Height | Conc. | Area% |
|---|---|---|---|---|---|
| 1 | 15.822 | 23460 | 1544 | 0.429 | 0.429 |
| 2 | 29.329 | 3947 | 300 | 0.072 | 0.072 |
| 3 | 30.765 | 4281 | 349 | 0.078 | 0.078 |
| 4 | 41.702 | 5429526 | 382926 | 99.319 | 99.319 |
| 5 | 46.202 | 3570 | 308 | 0.065 | 0.065 |
| 6 | 48.667 | 1985 | 189 | 0.036 | 0.036 |
| Total | | 5466768 | 385617 | | 100.000 |

# HPLC Purity of Compound 2

SHIMADZU LabSolutions

## Analysis Report

### <Sample Information>

Sample Name : 73-KS1-13
Sample ID :
Data Filename : 20221018_73-KS1-13_006.lcd
Method Filename : NHRI purity test.lcm
Batch Filename : 101822_KS1_2.lcb
Vial # : 1-15 Sample Type : Unknown
Injection Volume : 20 uL
Date Acquired : 2022/10/18 10:28:37 Acquired by : System Administrator
Date Processed : 2022/10/18 11:28:39 Processed by : System Administrator

### <Chromatogram>

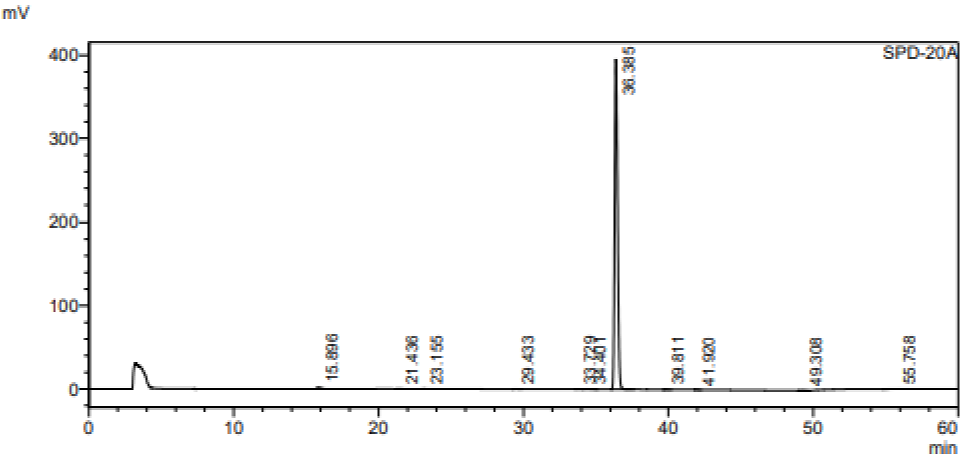


Peak Table

SPD-20A

| Peak# | Ret. Time | Area | Height | Conc. | Area% |
|---|---|---|---|---|---|
| 1 | 15.896 | 24744 | 1566 | 0.432 | 0.432 |
| 2 | 21.436 | 1998 | 148 | 0.035 | 0.035 |
| 3 | 23.155 | 2817 | 192 | 0.049 | 0.049 |
| 4 | 29.433 | 4297 | 308 | 0.075 | 0.075 |
| 5 | 33.729 | 1902 | 125 | 0.033 | 0.033 |
| 6 | 34.401 | 8671 | 353 | 0.152 | 0.152 |
| 7 | 36.385 | 5659731 | 396083 | 98.895 | 98.895 |
| 8 | 39.811 | 2274 | 162 | 0.040 | 0.040 |
| 9 | 41.920 | 1323 | 102 | 0.023 | 0.023 |
| 10 | 49.308 | 10748 | 515 | 0.188 | 0.188 |
| 11 | 55.758 | 4463 | 52 | 0.078 | 0.078 |
| Total | | 5722969 | 399607 | | 100.000 |

# HPLC Purity of Compound 3

SHIMADZU LabSolutions **Analysis Report**

### <Sample Information>

| | | | |
|---|---|---|---|
| Sample Name | : 73-KS1-14 | | |
| Sample ID | : | | |
| Data Filename | : 20221026_73-KS1-14_007.lcd | | |
| Method Filename | : NHRI purity test.lcm | | |
| Batch Filename | : 10262022_73KS1_1.lcb | | |
| Vial # | : 1-23 | Sample Type | : Unknown |
| Injection Volume | : 20 uL | | |
| Date Acquired | : 2022/10/26 10:50:38 | Acquired by | : System Administrator |
| Date Processed | : 2022/10/26 11:50:41 | Processed by | : System Administrator |

### <Chromatogram>

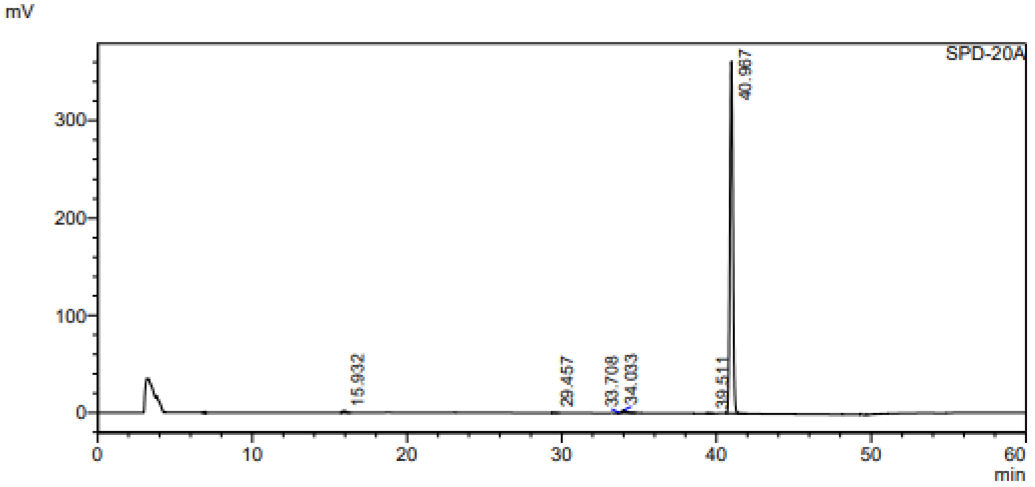


Peak Table

SPD-20A

| Peak# | Ret. Time | Area | Height | Conc. | Area% |
|---|---|---|---|---|---|
| 1 | 15.932 | 23978 | 1722 | 0.499 | 0.499 |
| 2 | 29.457 | 3680 | 324 | 0.077 | 0.077 |
| 3 | 33.708 | 3198 | 276 | 0.067 | 0.067 |
| 4 | 34.033 | 48118 | 3005 | 1.001 | 1.001 |
| 5 | 39.511 | 4020 | 365 | 0.084 | 0.084 |
| 6 | 40.967 | 4725102 | 361569 | 98.274 | 98.274 |
| Total | | 4808096 | 367261 | | 100.000 |

## HPLC Purity of Compound 4

SHIMADZU LabSolutions **Analysis Report**

### <Sample Information>

Sample Name : 73-KS1-15
Sample ID :
Data Filename : 20221026_73-KS1-15_008.lcd
Method Filename : NHRI purity test.lcm
Batch Filename : 10262022_73KS1_1.lcb
Vial # : 1-24
Sample Type : Unknown
Injection Volume : 20 uL
Date Acquired : 2022/10/26 11:51:03
Acquired by : System Administrator
Date Processed : 2022/10/27 12:51:05
Processed by : System Administrator

### <Chromatogram>

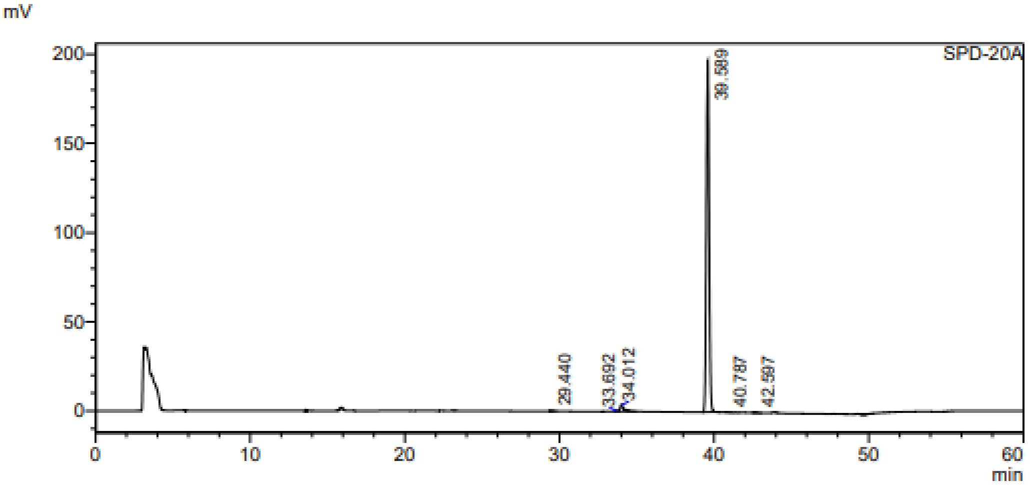


Peak Table

SPD-20A

| Peak# | Ret. Time | Area | Height | Conc. | Area% |
|---|---|---|---|---|---|
| 1 | 29.440 | 3789 | 326 | 0.153 | 0.153 |
| 2 | 33.692 | 3436 | 301 | 0.139 | 0.139 |
| 3 | 34.012 | 59443 | 3554 | 2.402 | 2.402 |
| 4 | 39.589 | 2400234 | 196793 | 97.006 | 97.006 |
| 5 | 40.787 | 2681 | 261 | 0.108 | 0.108 |
| 6 | 42.597 | 4745 | 365 | 0.192 | 0.192 |
| Total | | 2474327 | 201600 | | 100.000 |

# HPLC Purity of Compound 5

SHIMADZU LabSolutions **Analysis Report**

## <Sample Information>

| | | | |
|---|---|---|---|
| Sample Name | : 73-KS1-17 | | |
| Sample ID | : | | |
| Data Filename | : 20221018_73-KS1-17_007.lcd | | |
| Method Filename | : NHRI purity test.lcm | | |
| Batch Filename | : 101822_KS1_2.lcb | | |
| Vial # | : 1-16 | Sample Type | : Unknown |
| Injection Volume | : 20 uL | | |
| Date Acquired | : 2022/10/18 11:29:00 | Acquired by | : System Administrator |
| Date Processed | : 2022/10/19 12:29:02 | Processed by | : System Administrator |

## <Chromatogram>

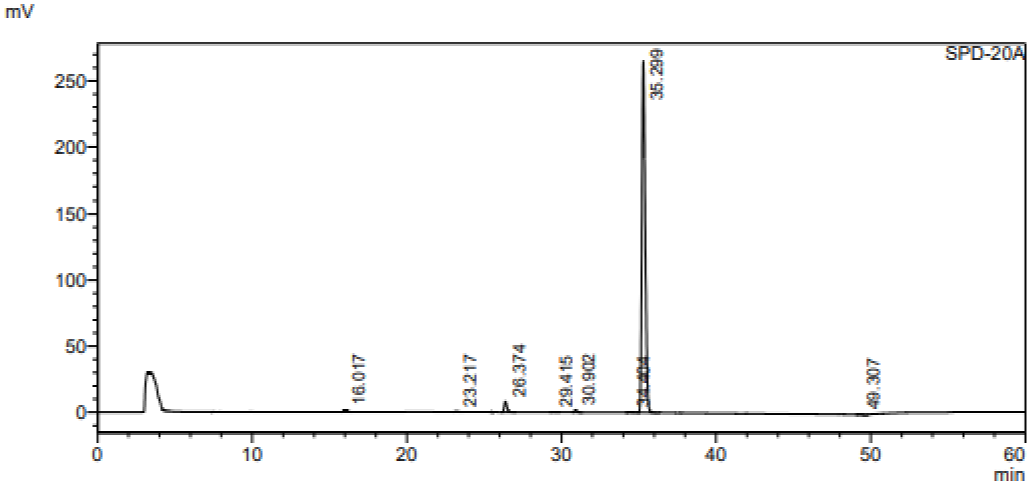


Peak Table

SPD-20A

| Peak# | Ret. Time | Area | Height | Conc. | Area% |
|---|---|---|---|---|---|
| 1 | 16.017 | 23972 | 1575 | 0.584 | 0.584 |
| 2 | 23.217 | 7661 | 561 | 0.187 | 0.187 |
| 3 | 26.374 | 98214 | 8076 | 2.391 | 2.391 |
| 4 | 29.415 | 4023 | 292 | 0.098 | 0.098 |
| 5 | 30.902 | 21445 | 1762 | 0.522 | 0.522 |
| 6 | 34.404 | 6734 | 316 | 0.164 | 0.164 |
| 7 | 35.299 | 3936890 | 265168 | 95.855 | 95.855 |
| 8 | 49.307 | 8186 | 383 | 0.199 | 0.199 |
| Total | | 4107124 | 278131 | | 100.000 |

# HPLC Purity of Compound 6

SHIMADZU LabSolutions

## Analysis Report

### <Sample Information>

| | | | |
|---|---|---|---|
| Sample Name | : 73-KS1-12 | | |
| Sample ID | : | | |
| Data Filename | : 20221018_73-KS1-12_005.lcd | | |
| Method Filename | : NHRI purity test.lcm | | |
| Batch Filename | : 101822_KS1_2.lcb | | |
| Vial # | : 1-14 | Sample Type | : Unknown |
| Injection Volume | : 20 uL | | |
| Date Acquired | : 2022/10/18 09:28:14 | Acquired by | : System Administrator |
| Date Processed | : 2022/10/18 10:28:16 | Processed by | : System Administrator |

### <Chromatogram>

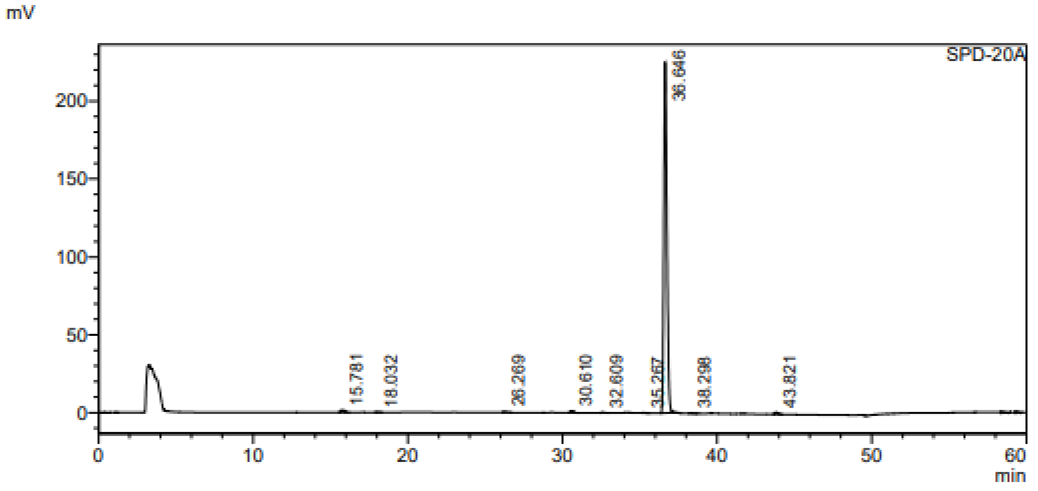


Peak Table

SPD-20A

| Peak# | Ret. Time | Area | Height | Conc. | Area% |
|---|---|---|---|---|---|
| 1 | 15.781 | 23388 | 1529 | 0.730 | 0.730 |
| 2 | 18.032 | 3929 | 330 | 0.123 | 0.123 |
| 3 | 26.269 | 8249 | 634 | 0.258 | 0.258 |
| 4 | 30.610 | 16191 | 1267 | 0.505 | 0.505 |
| 5 | 32.609 | 6438 | 472 | 0.201 | 0.201 |
| 6 | 35.267 | 3616 | 289 | 0.113 | 0.113 |
| 7 | 36.646 | 3120412 | 225204 | 97.416 | 97.416 |
| 8 | 38.298 | 4265 | 359 | 0.133 | 0.133 |
| 9 | 43.821 | 16706 | 1154 | 0.522 | 0.522 |
| Total | | 3203193 | 231239 | | 100.000 |

# HPLC Purity of Compound 7

SHIMADZU LabSolutions **Analysis Report**

## <Sample Information>

| | | | |
|---|---|---|---|
| Sample Name | : 73-KS1-26 | | |
| Sample ID | : | | |
| Data Filename | : 20221018_73-KS1-26_008.lcd | | |
| Method Filename | : NHRI purity test.lcm | | |
| Batch Filename | : 101822_KS1_2.lcb | | |
| Vial # | : 1-17 | Sample Type | : Unknown |
| Injection Volume | : 20 uL | | |
| Date Acquired | : 2022/10/19 12:29:24 | Acquired by | : System Administrator |
| Date Processed | : 2022/10/19 01:29:27 | Processed by | : System Administrator |

## <Chromatogram>

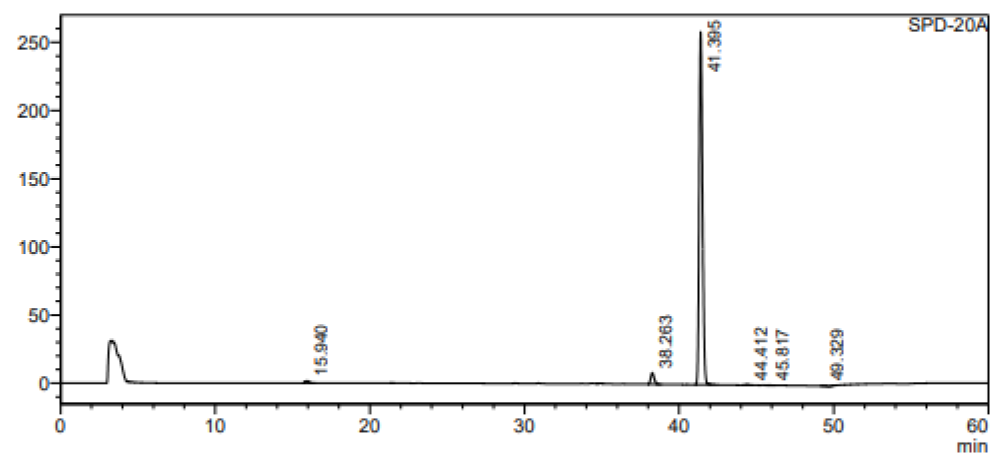


Peak Table

SPD-20A

| Peak# | Ret. Time | Area | Height | Conc. | Area% |
|---|---|---|---|---|---|
| 1 | 15.940 | 24184 | 1582 | 0.599 | 0.599 |
| 2 | 38.263 | 126360 | 8417 | 3.128 | 3.128 |
| 3 | 41.395 | 3879395 | 258043 | 96.040 | 96.040 |
| 4 | 44.412 | 2792 | 221 | 0.069 | 0.069 |
| 5 | 45.817 | 1564 | 132 | 0.039 | 0.039 |
| 6 | 49.329 | 5058 | 267 | 0.125 | 0.125 |
| Total | | 4039352 | 268664 | | 100.000 |

# HPLC Purity of Compound 8

SHIMADZU LabSolutions **Analysis Report**

## <Sample Information>

Sample Name : 73-KS-12
Sample ID :
Data Filename : 20221018_73-KS-12_001.lcd
Method Filename : NHRI purity test.lcm
Batch Filename : 101822_KS1_2.lcb
Vial # : 1-10 Sample Type : Unknown
Injection Volume : 20 uL
Date Acquired : 2022/10/18 05:26:40 Acquired by : System Administrator
Date Processed : 2022/10/18 06:26:42 Processed by : System Administrator

## <Chromatogram>

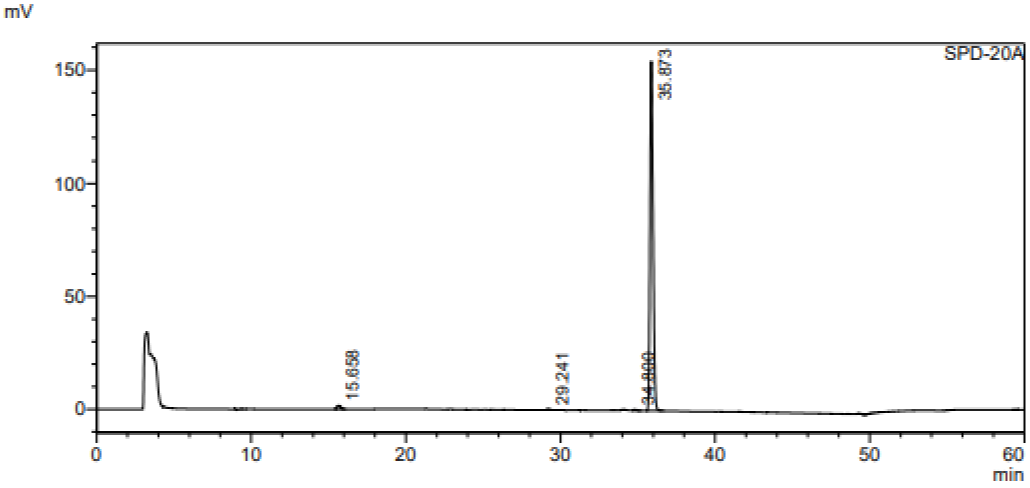


Peak Table

SPD-20A

| Peak# | Ret. Time | Area | Height | Conc. | Area% |
|---|---|---|---|---|---|
| 1 | 15.658 | 23352 | 1520 | 1.060 | 1.060 |
| 2 | 29.241 | 5271 | 441 | 0.239 | 0.239 |
| 3 | 34.800 | 5800 | 420 | 0.263 | 0.263 |
| 4 | 35.873 | 2169055 | 154568 | 98.438 | 98.438 |
| Total | | 2203478 | 156948 | | 100.000 |

# HPLC Purity of Compound 9

SHIMADZU LabSolutions **Analysis Report**

**<Sample Information>**

| | | | |
|---|---|---|---|
| Sample Name | : 73-KS1-28 | | |
| Sample ID | : | | |
| Data Filename | : 20221017_73-KS1-28_001.lcd | | |
| Method Filename | : NHRI purity test.lcm | | |
| Batch Filename | : 101722_73KS1_1.lcb | | |
| Vial # | : 1-10 | Sample Type | : Unknown |
| Injection Volume | : 20 uL | | |
| Date Acquired | : 2022/10/17 11:23:12 | Acquired by | : System Administrator |
| Date Processed | : 2022/10/18 12:23:15 | Processed by | : System Administrator |

**<Chromatogram>**

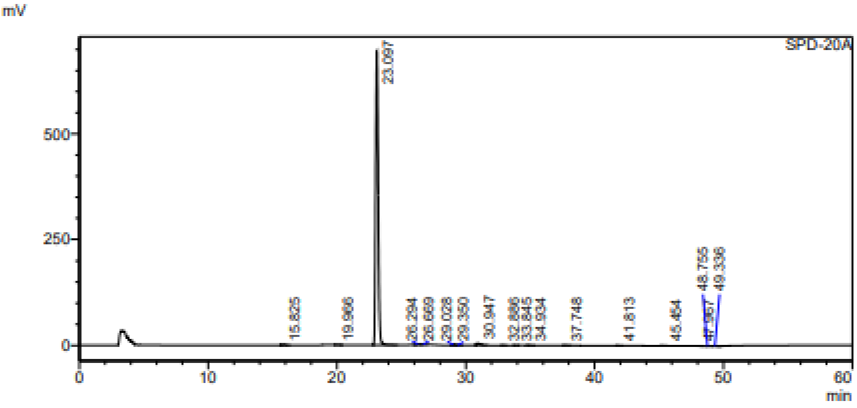


Peak Table

SPD-20A

| Peak# | Ret. Time | Area | Height | Conc. | Area% |
|---|---|---|---|---|---|
| 1 | 15.825 | 23899 | 1568 | 0.244 | 0.244 |
| 2 | 19.966 | 3520 | 225 | 0.036 | 0.036 |
| 3 | 23.097 | 9582441 | 694346 | 97.789 | 97.789 |
| 4 | 26.294 | 17330 | 1387 | 0.177 | 0.177 |
| 5 | 26.669 | 9864 | 620 | 0.101 | 0.101 |
| 6 | 29.028 | 18845 | 1422 | 0.192 | 0.192 |
| 7 | 29.350 | 3242 | 275 | 0.033 | 0.033 |
| 8 | 30.947 | 93831 | 4705 | 0.958 | 0.958 |
| 9 | 32.886 | 3023 | 238 | 0.031 | 0.031 |
| 10 | 33.845 | 2206 | 210 | 0.023 | 0.023 |
| 11 | 34.934 | 3491 | 247 | 0.036 | 0.036 |
| 12 | 37.748 | 8325 | 575 | 0.085 | 0.085 |
| 13 | 41.813 | 5368 | 432 | 0.055 | 0.055 |
| 14 | 45.454 | 10211 | 429 | 0.104 | 0.104 |
| 15 | 47.967 | 4381 | 246 | 0.045 | 0.045 |
| 16 | 48.755 | 2028 | 175 | 0.021 | 0.021 |
| 17 | 49.336 | 7108 | 392 | 0.073 | 0.073 |
| Total | | 9799115 | 707492 | | 100.000 |

# HPLC Purity of Compound 10

SHIMADZU LabSolutions **Analysis Report**

## <Sample Information>

| | | | |
|---|---|---|---|
| Sample Name | : 73-KS-14 | | |
| Sample ID | : | | |
| Data Filename | : 20221017_73-KS-14_006.lcd | | |
| Method Filename | : NHRI purity test.lcm | | |
| Batch Filename | : 101722_73KS1_1.lcb | | |
| Vial # | : 1-15 | Sample Type | : Unknown |
| Injection Volume | : 20 uL | | |
| Date Acquired | : 2022/10/18 04:25:08 | Acquired by | : System Administrator |
| Date Processed | : 2022/10/18 05:25:10 | Processed by | : System Administrator |

## <Chromatogram>

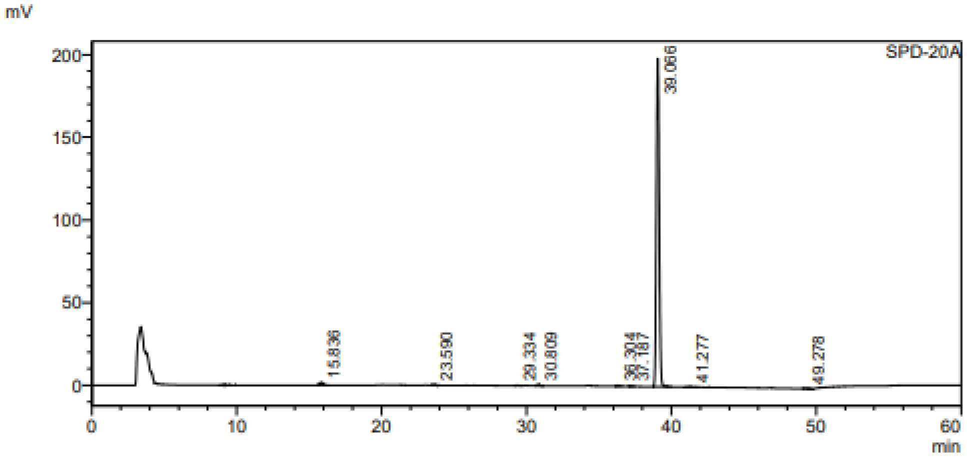


Peak Table

SPD-20A

| Peak# | Ret. Time | Area | Height | Conc. | Area% |
|---|---|---|---|---|---|
| 1 | 15.836 | 23710 | 1544 | 0.855 | 0.855 |
| 2 | 23.590 | 5497 | 478 | 0.198 | 0.198 |
| 3 | 29.334 | 4058 | 310 | 0.146 | 0.146 |
| 4 | 30.809 | 9336 | 741 | 0.337 | 0.337 |
| 5 | 36.304 | 6524 | 556 | 0.235 | 0.235 |
| 6 | 37.187 | 2222 | 199 | 0.080 | 0.080 |
| 7 | 39.066 | 2707452 | 198929 | 97.658 | 97.658 |
| 8 | 41.277 | 2854 | 230 | 0.103 | 0.103 |
| 9 | 49.278 | 10736 | 448 | 0.387 | 0.387 |
| Total | | 2772389 | 203433 | | 100.000 |

# HPLC Purity of Compound 11

SHIMADZU LabSolutions **Analysis Report**

## <Sample Information>

| | | | |
|---|---|---|---|
| Sample Name | : 73-KS-16 | | |
| Sample ID | : | | |
| Data Filename | : 20221018_73-KS-16_003.lcd | | |
| Method Filename | : NHRI purity test.lcm | | |
| Batch Filename | : 101822_KS1_2.lcb | | |
| Vial # | : 1-12 | Sample Type | : Unknown |
| Injection Volume | : 20 uL | | |
| Date Acquired | : 2022/10/18 07:27:29 | Acquired by | : System Administrator |
| Date Processed | : 2022/10/18 08:27:31 | Processed by | : System Administrator |

## <Chromatogram>

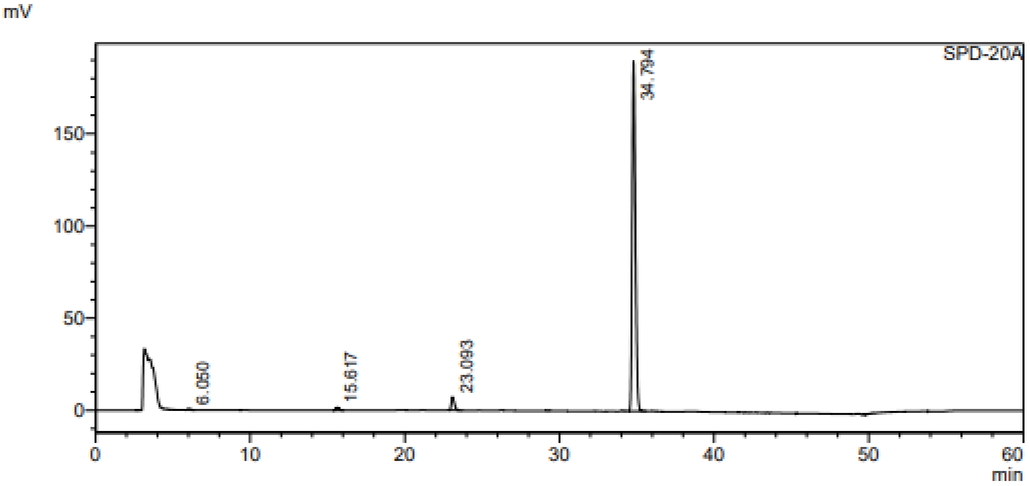


Peak Table

SPD-20A

| Peak# | Ret. Time | Area | Height | Conc. | Area% |
|---|---|---|---|---|---|
| 1 | 6.050 | 5898 | 572 | 0.204 | 0.204 |
| 2 | 15.617 | 23140 | 1512 | 0.799 | 0.799 |
| 3 | 23.093 | 93528 | 6968 | 3.230 | 3.230 |
| 4 | 34.794 | 2772716 | 189845 | 95.767 | 95.767 |
| Total | | 2895283 | 198896 | | 100.000 |

# HPLC Purity of Compound 12

SHIMADZU LabSolutions **Analysis Report**

## <Sample Information>

| | | | |
|---|---|---|---|
| Sample Name | : 73-KS-11 | | |
| Sample ID | : | | |
| Data Filename | : 20221017_73-KS-11_003.lcd | | |
| Method Filename | : NHRI purity test.lcm | | |
| Batch Filename | : 101722_73KS1_1.lcb | | |
| Vial # | : 1-12 | Sample Type | : Unknown |
| Injection Volume | : 20 uL | | |
| Date Acquired | : 2022/10/18 01:23:58 | Acquired by | : System Administrator |
| Date Processed | : 2022/10/18 02:24:00 | Processed by | : System Administrator |

## <Chromatogram>

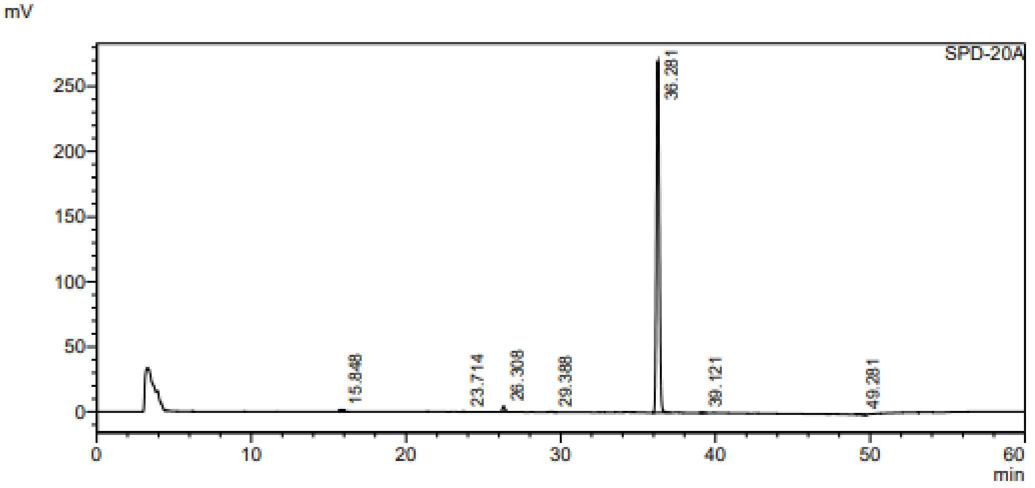


Peak Table

SPD-20A

| Peak# | Ret. Time | Area | Height | Conc. | Area% |
|---|---|---|---|---|---|
| 1 | 15.848 | 23499 | 1534 | 0.647 | 0.647 |
| 2 | 23.714 | 3263 | 318 | 0.090 | 0.090 |
| 3 | 26.308 | 52705 | 4376 | 1.452 | 1.452 |
| 4 | 29.388 | 4770 | 333 | 0.131 | 0.131 |
| 5 | 36.281 | 3534695 | 269957 | 97.352 | 97.352 |
| 6 | 39.121 | 3763 | 323 | 0.104 | 0.104 |
| 7 | 49.281 | 8145 | 423 | 0.224 | 0.224 |
| Total | | 3630838 | 277264 | | 100.000 |

# HPLC Purity of Compound 13

SHIMADZU LabSolutions **Analysis Report**

## <Sample Information>

Sample Name : 73-KS1-07
Sample ID :
Data Filename : 20221026_73-KS1-07_005.lcd
Method Filename : NHRI purity test.lcm
Batch Filename : 10262022_73KS1_1.lcb
Vial # : 1-21 Sample Type : Unknown
Injection Volume : 20 uL
Date Acquired : 2022/10/26 08:49:49 Acquired by : System Administrator
Date Processed : 2022/10/27 10:44:45 Processed by : System Administrator

## <Chromatogram>

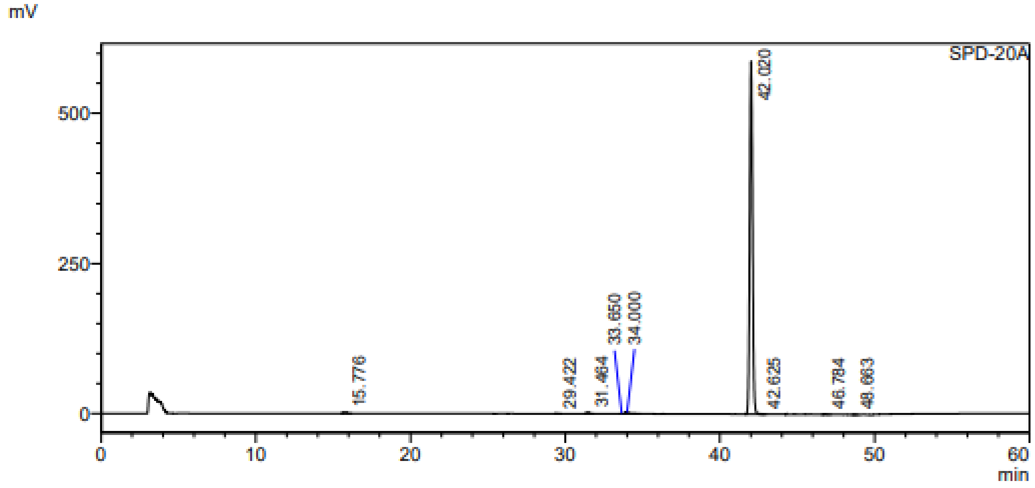


Peak Table

SPD-20A

| Peak# | Ret. Time | Area | Height | Conc. | Area% |
|---|---|---|---|---|---|
| 1 | 15.776 | 24207 | 1734 | 0.317 | 0.317 |
| 2 | 29.422 | 3682 | 343 | 0.048 | 0.048 |
| 3 | 31.464 | 26801 | 2374 | 0.351 | 0.351 |
| 4 | 33.650 | 3415 | 275 | 0.045 | 0.045 |
| 5 | 34.000 | 43007 | 2610 | 0.563 | 0.563 |
| 6 | 42.020 | 7525837 | 588049 | 98.500 | 98.500 |
| 7 | 42.625 | 1391 | 195 | 0.018 | 0.018 |
| 8 | 46.784 | 9672 | 786 | 0.127 | 0.127 |
| 9 | 48.663 | 2440 | 246 | 0.032 | 0.032 |
| Total | | 7640453 | 596612 | | 100.000 |

# HPLC Purity of Compound 14

SHIMADZU LabSolutions

## Analysis Report

### <Sample Information>

| | | | |
|---|---|---|---|
| Sample Name | : 73-KS1-04 | | |
| Sample ID | : | | |
| Data Filename | : 20221017_73-KS1-04_002.lcd | | |
| Method Filename | : NHRI purity test.lcm | | |
| Batch Filename | : 101722_73KS1_1.lcb | | |
| Vial # | : 1-11 | Sample Type | : Unknown |
| Injection Volume | : 20 uL | | |
| Date Acquired | : 2022/10/18 12:23:35 | Acquired by | : System Administrator |
| Date Processed | : 2022/10/18 01:23:38 | Processed by | : System Administrator |

### <Chromatogram>

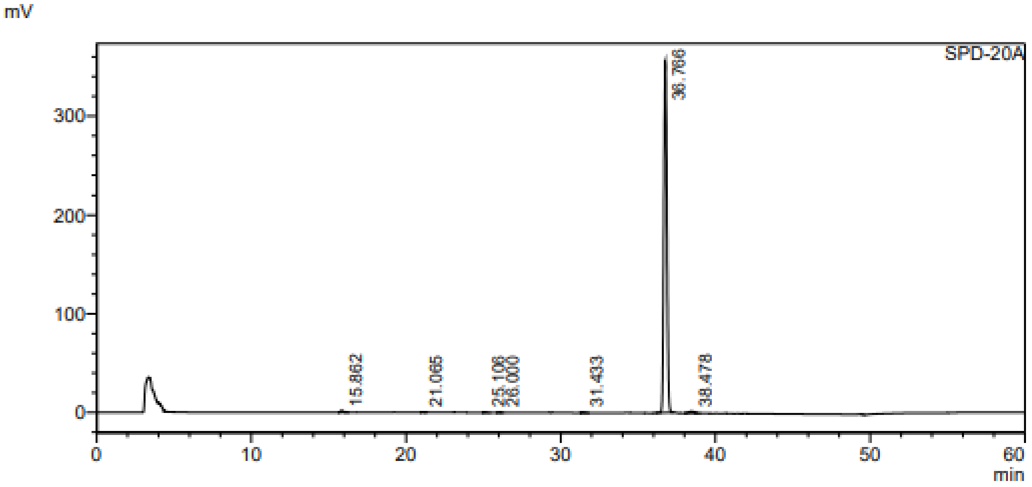


Peak Table

SPD-20A

| Peak# | Ret. Time | Area | Height | Conc. | Area% |
|---|---|---|---|---|---|
| 1 | 15.862 | 23866 | 1551 | 0.486 | 0.486 |
| 2 | 21.065 | 4939 | 525 | 0.101 | 0.101 |
| 3 | 25.106 | 7258 | 649 | 0.148 | 0.148 |
| 4 | 26.000 | 5681 | 530 | 0.116 | 0.116 |
| 5 | 31.433 | 8209 | 621 | 0.167 | 0.167 |
| 6 | 36.766 | 4815865 | 355685 | 98.077 | 98.077 |
| 7 | 38.478 | 44496 | 1872 | 0.906 | 0.906 |
| Total | | 4910313 | 361433 | | 100.000 |

# HPLC Purity of Compound 15

Shimadzu LabSolutions

## Analysis Report

### <Sample Information>

| | | | |
|---|---|---|---|
| Sample Name | : 73-KS1-05 | | |
| Sample ID | : | | |
| Data Filename | : 20221026_73-KS1-05_003.lcd | | |
| Method Filename | : NHRI purity test.lcm | | |
| Batch Filename | : 10262022_73KS1_1.lcb | | |
| Vial # | : 1-19 | Sample Type | : Unknown |
| Injection Volume | : 20 uL | | |
| Date Acquired | : 2022/10/26 06:48:57 | Acquired by | : System Administrator |
| Date Processed | : 2022/10/26 07:49:02 | Processed by | : System Administrator |

### <Chromatogram>

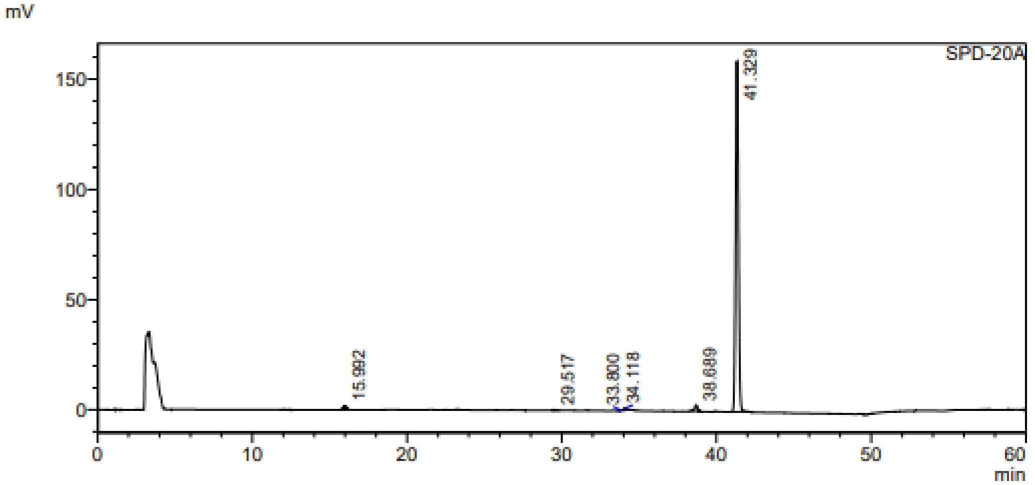


Peak Table

SPD-20A

| Peak# | Ret. Time | Area | Height | Conc. | Area% |
|---|---|---|---|---|---|
| 1 | 15.992 | 24546 | 1725 | 1.168 | 1.168 |
| 2 | 29.517 | 3202 | 309 | 0.152 | 0.152 |
| 3 | 33.800 | 1782 | 180 | 0.085 | 0.085 |
| 4 | 34.118 | 8026 | 753 | 0.382 | 0.382 |
| 5 | 38.689 | 30920 | 2641 | 1.472 | 1.472 |
| 6 | 41.329 | 2032638 | 158962 | 96.741 | 96.741 |
| Total | | 2101116 | 164570 | | 100.000 |

# HPLC Purity of Compound 16

SHIMADZU LabSolutions **Analysis Report**

### <Sample Information>

| | | | |
|---|---|---|---|
| Sample Name | : 73-KS1-06 | | |
| Sample ID | : | | |
| Data Filename | : 20221026_73-KS1-06_004.lcd | | |
| Method Filename | : NHRI purity test.lcm | | |
| Batch Filename | : 10262022_73KS1_1.lcb | | |
| Vial # | : 1-20 | Sample Type | : Unknown |
| Injection Volume | : 20 uL | | |
| Date Acquired | : 2022/10/26 07:49:24 | Acquired by | : System Administrator |
| Date Processed | : 2022/10/27 10:45:02 | Processed by | : System Administrator |

### <Chromatogram>

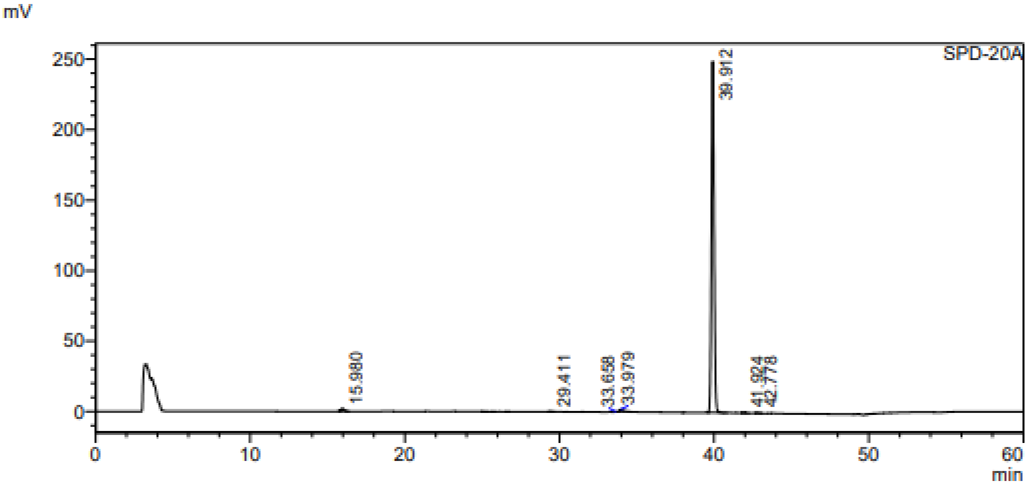


Peak Table

SPD-20A

| Peak# | Ret. Time | Area | Height | Conc. | Area% |
|---|---|---|---|---|---|
| 1 | 15.980 | 24229 | 1738 | 0.794 | 0.794 |
| 2 | 29.411 | 2954 | 302 | 0.097 | 0.097 |
| 3 | 33.658 | 1914 | 190 | 0.063 | 0.063 |
| 4 | 33.979 | 17836 | 1602 | 0.584 | 0.584 |
| 5 | 39.912 | 2995941 | 249445 | 98.136 | 98.136 |
| 6 | 41.924 | 6281 | 462 | 0.206 | 0.206 |
| 7 | 42.778 | 3691 | 346 | 0.121 | 0.121 |
| Total | | 3052846 | 254086 | | 100.000 |

# HPLC Purity of Compound 17

SHIMADZU LabSolutions **Analysis Report**

### <Sample Information>

| | | | |
|---|---|---|---|
| Sample Name | : 73-KS1-08 | | |
| Sample ID | : | | |
| Data Filename | : 20221026_73-KS1-08_006.lcd | | |
| Method Filename | : NHRI purity test.lcm | | |
| Batch Filename | : 10262022_73KS1_1.lcb | | |
| Vial # | : 1-22 | Sample Type | : Unknown |
| Injection Volume | : 20 uL | | |
| Date Acquired | : 2022/10/26 09:50:13 | Acquired by | : System Administrator |
| Date Processed | : 2022/10/26 10:50:17 | Processed by | : System Administrator |

### <Chromatogram>

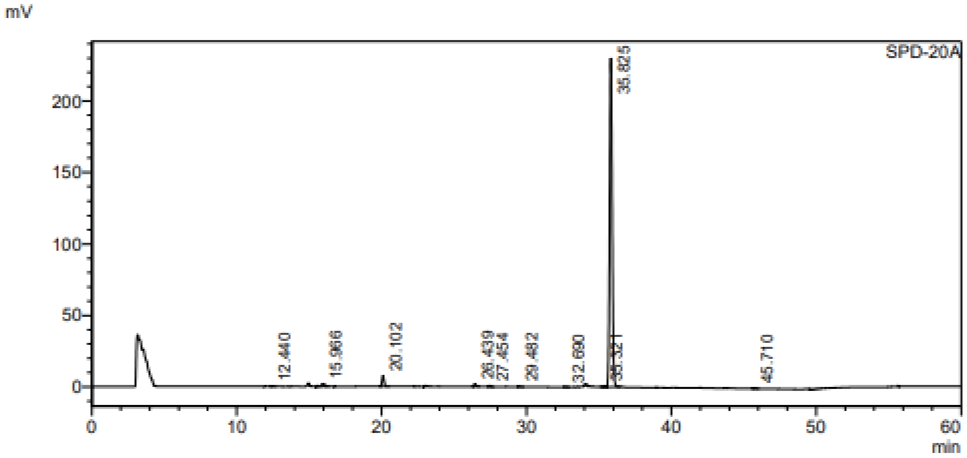


Peak Table

SPD-20A

| Peak# | Ret. Time | Area | Height | Conc. | Area% |
|---|---|---|---|---|---|
| 1 | 12.440 | 4458 | 477 | 0.150 | 0.150 |
| 2 | 15.966 | 23836 | 1732 | 0.800 | 0.800 |
| 3 | 20.102 | 77449 | 7529 | 2.601 | 2.601 |
| 4 | 26.439 | 16000 | 1665 | 0.537 | 0.537 |
| 5 | 27.454 | 7648 | 812 | 0.257 | 0.257 |
| 6 | 29.482 | 4620 | 450 | 0.155 | 0.155 |
| 7 | 32.690 | 2778 | 279 | 0.093 | 0.093 |
| 8 | 35.321 | 3622 | 356 | 0.122 | 0.122 |
| 9 | 35.825 | 2833764 | 230165 | 95.157 | 95.157 |
| 10 | 45.710 | 3810 | 347 | 0.128 | 0.128 |
| Total | | 2977984 | 243812 | | 100.000 |

# HPLC Purity of Compound 18

SHIMADZU LabSolutions **Analysis Report**

### <Sample Information>

Sample Name : 73-Flavone
Sample ID :
Data Filename : 202359_73-Flavone_004.lcd
Method Filename : NHRI purity test.lcm
Batch Filename : 0509 Flavone.lcb
Vial # : 1-40
Sample Type : Unknown
Injection Volume : 20 uL
Date Acquired : 2023/5/9 08:08:42
Acquired by : System Administrator
Date Processed : 2023/6/30 03:03:53
Processed by : System Administrator

### <Chromatogram>

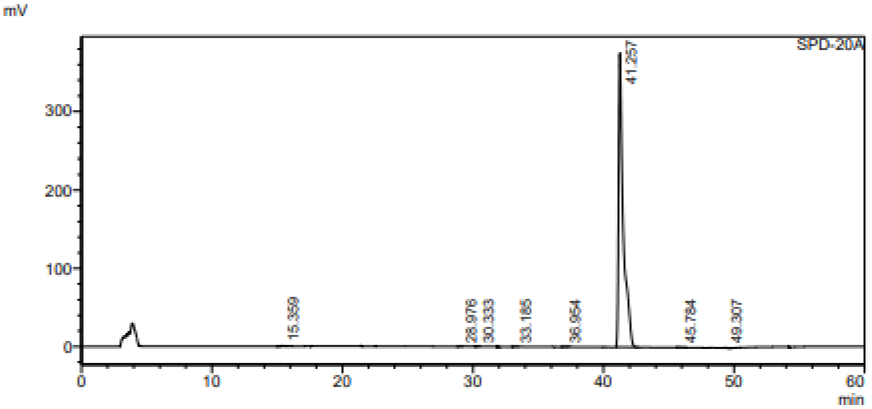


Peak Table

SPD-20A

| Peak# | Ret. Time | Area | Height | Conc. | Area% |
|---|---|---|---|---|---|
| 1 | 15.359 | 15697 | 826 | 0.167 | 0.167 |
| 2 | 28.976 | 1705 | 176 | 0.018 | 0.018 |
| 3 | 30.333 | 1881 | 201 | 0.020 | 0.020 |
| 4 | 33.185 | 1924 | 154 | 0.020 | 0.020 |
| 5 | 36.954 | 8093 | 458 | 0.086 | 0.086 |
| 6 | 41.257 | 9350582 | 375514 | 99.551 | 99.551 |
| 7 | 45.784 | 4136 | 312 | 0.044 | 0.044 |
| 8 | 49.307 | 8708 | 430 | 0.093 | 0.093 |
| Total | | 9392725 | 378071 | | 100.000 |

# HPLC Purity of Compound 19

SHIMADZU LabSolutions **Analysis Report**

## <Sample Information>

| | | | |
|---|---|---|---|
| Sample Name | : 73-KS1-25 | | |
| Sample ID | : | | |
| Data Filename | : 20221026_73-KS1-25_010.lcd | | |
| Method Filename | : NHRI purity test.lcm | | |
| Batch Filename | : 10262022_73KS1_1.lcb | | |
| Vial # | : 1-26 | Sample Type | : Unknown |
| Injection Volume | : 20 uL | | |
| Date Acquired | : 2022/10/27 01:51:53 | Acquired by | : System Administrator |
| Date Processed | : 2022/10/27 02:51:56 | Processed by | : System Administrator |

## <Chromatogram>

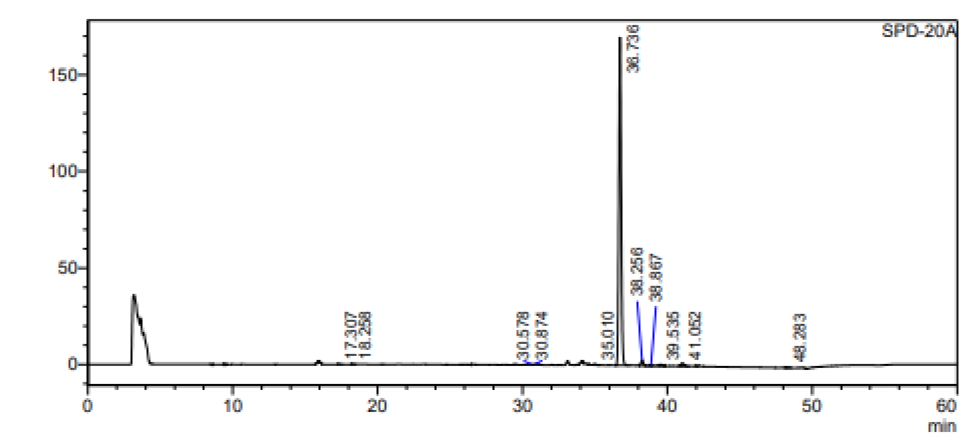


Peak Table

SPD-20A

| Peak# | Ret. Time | Area | Height | Conc. | Area% |
|---|---|---|---|---|---|
| 1 | 17.307 | 1871 | 209 | 0.095 | 0.095 |
| 2 | 18.258 | 2779 | 297 | 0.142 | 0.142 |
| 3 | 30.578 | 3194 | 355 | 0.163 | 0.163 |
| 4 | 30.874 | 2131 | 267 | 0.109 | 0.109 |
| 5 | 35.010 | 4345 | 391 | 0.221 | 0.221 |
| 6 | 36.736 | 1886634 | 170034 | 96.080 | 96.080 |
| 7 | 38.256 | 31822 | 2925 | 1.621 | 1.621 |
| 8 | 38.867 | 3415 | 346 | 0.174 | 0.174 |
| 9 | 39.535 | 8351 | 726 | 0.425 | 0.425 |
| 10 | 41.052 | 16202 | 1322 | 0.825 | 0.825 |
| 11 | 48.283 | 2865 | 289 | 0.146 | 0.146 |
| Total | | 1963610 | 177161 | | 100.000 |

# HPLC Purity of Compound 20

SHIMADZU LabSolutions **Analysis Report**

## <Sample Information>

| | | | |
|---|---|---|---|
| Sample Name | : 73-KS1-27 | | |
| Sample ID | : | | |
| Data Filename | : 20221026_73-KS1-27_011.lcd | | |
| Method Filename | : NHRI purity test.lcm | | |
| Batch Filename | : 10262022_73KS1_1.lcb | | |
| Vial # | : 1-27 | Sample Type | : Unknown |
| Injection Volume | : 20 uL | | |
| Date Acquired | : 2022/10/27 02:52:19 | Acquired by | : System Administrator |
| Date Processed | : 2022/10/27 10:42:27 | Processed by | : System Administrator |

## <Chromatogram>

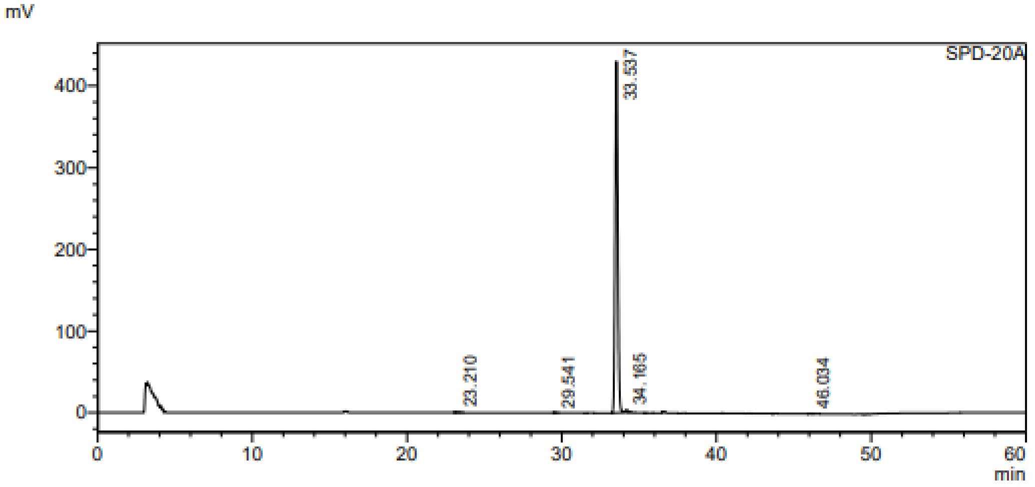


Peak Table

SPD-20A

| Peak# | Ret. Time | Area | Height | Conc. | Area% |
|---|---|---|---|---|---|
| 1 | 23.210 | 10130 | 735 | 0.193 | 0.193 |
| 2 | 29.541 | 2387 | 261 | 0.046 | 0.046 |
| 3 | 33.537 | 5184228 | 430198 | 98.924 | 98.924 |
| 4 | 34.165 | 37928 | 3056 | 0.724 | 0.724 |
| 5 | 46.034 | 5948 | 480 | 0.113 | 0.113 |
| Total | | 5240621 | 434730 | | 100.000 |

**HPLC Purity of Compound 21**

SHIMADZU LabSolutions **Analysis Report**

### <Sample Information>

| | | | | |
|---|---|---|---|---|
| Sample Name | : 73-KS1-23 | | | |
| Sample ID | : | | | |
| Data Filename | : 20221026_73-KS1-23_009.lcd | | | |
| Method Filename | : NHRI purity test.lcm | | | |
| Batch Filename | : 10262022_73KS1_1.lcb | | | |
| Vial # | : 1-25 | | Sample Type | : Unknown |
| Injection Volume | : 20 uL | | | |
| Date Acquired | : 2022/10/27 | 12:51:28 | Acquired by | : System Administrator |
| Date Processed | : 2022/10/27 | 01:51:30 | Processed by | : System Administrator |

### <Chromatogram>

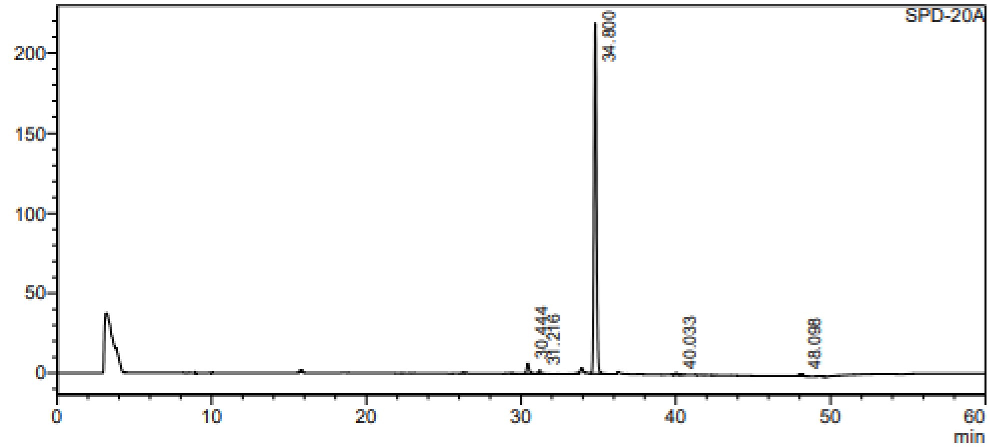


Peak Table

SPD-20A

| Peak# | Ret. Time | Area | Height | Conc. | Area% |
|---|---|---|---|---|---|
| 1 | 30.444 | 72631 | 6364 | 2.908 | 2.908 |
| 2 | 31.216 | 21903 | 2163 | 0.877 | 0.877 |
| 3 | 34.800 | 2374815 | 219003 | 95.096 | 95.096 |
| 4 | 40.033 | 11757 | 933 | 0.471 | 0.471 |
| 5 | 48.098 | 16182 | 1256 | 0.648 | 0.648 |
| Total | | 2497287 | 229719 | | 100.000 |

# HRMS Data of Compound 1

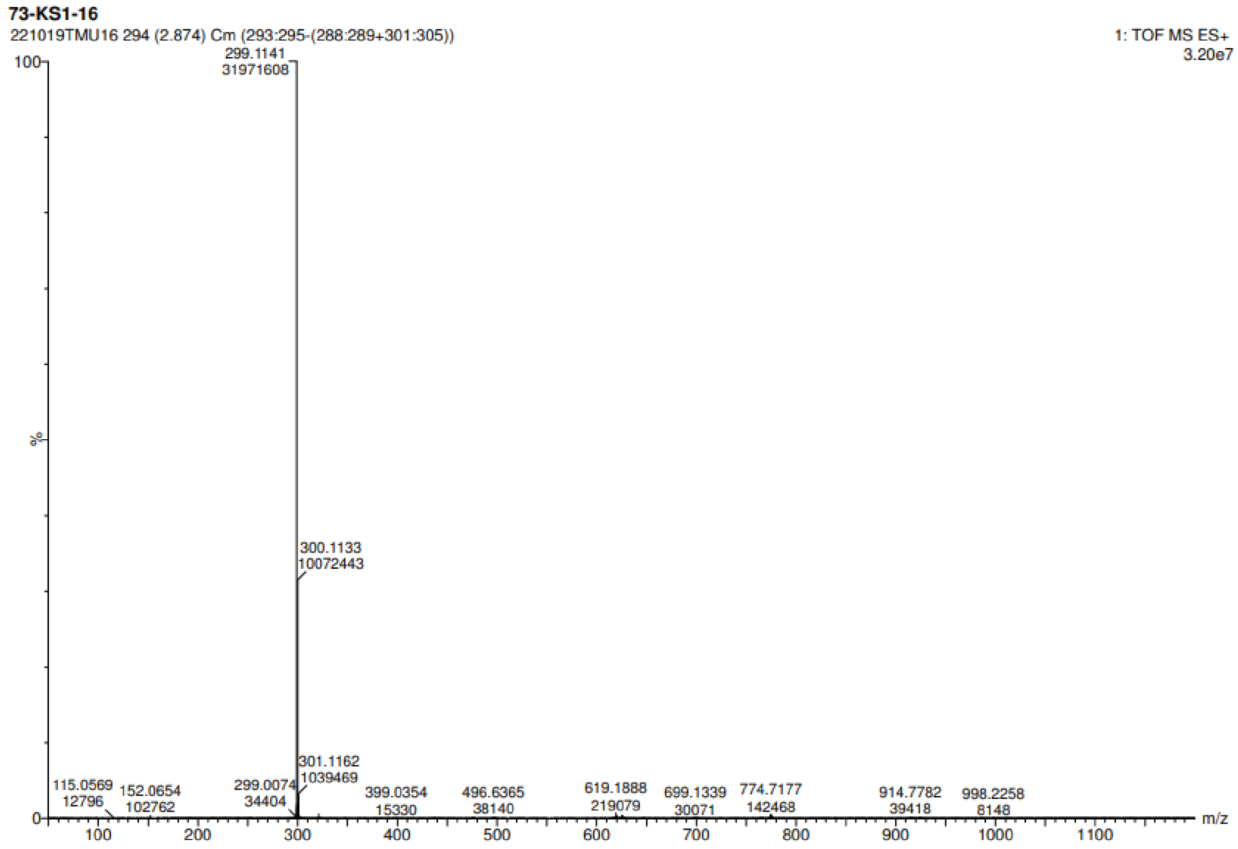

# HRMS Data of Compound 2

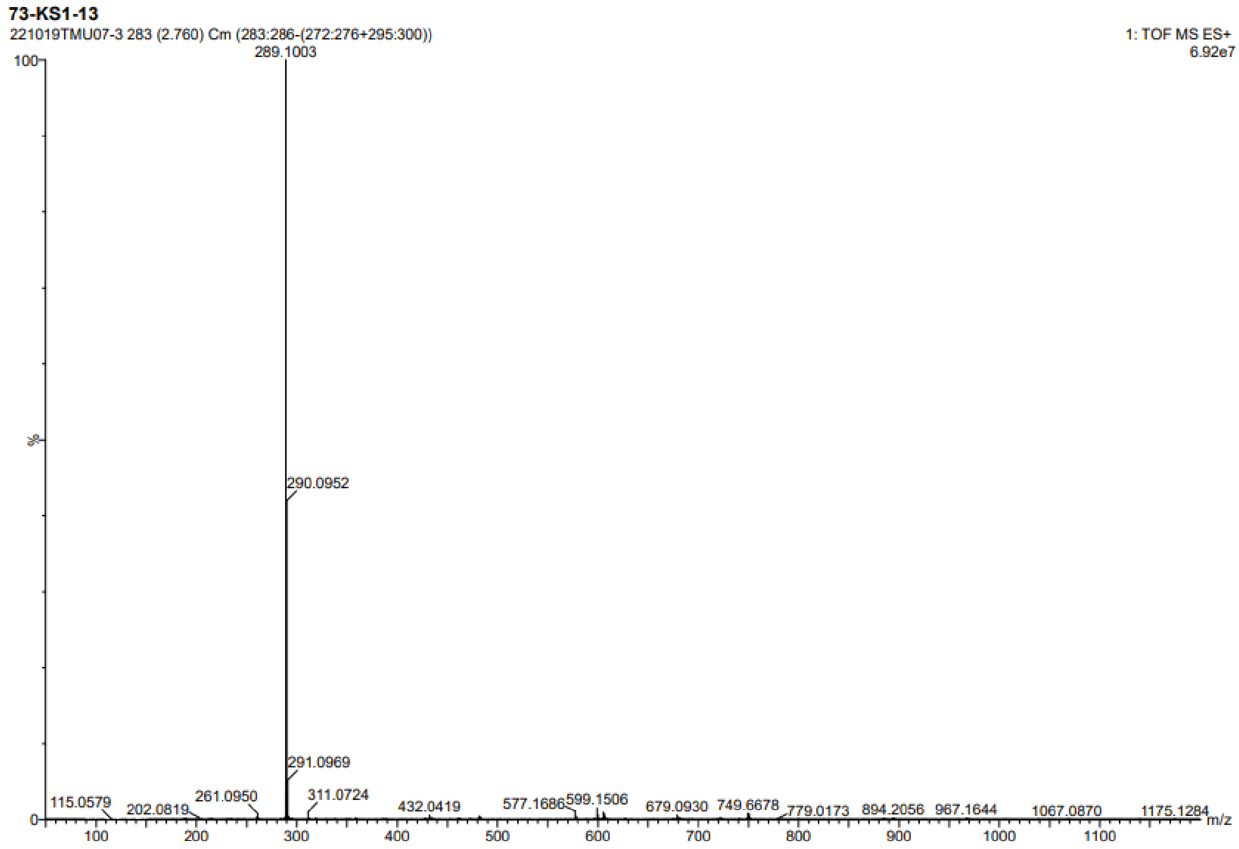

# HRMS Data of Compound 3

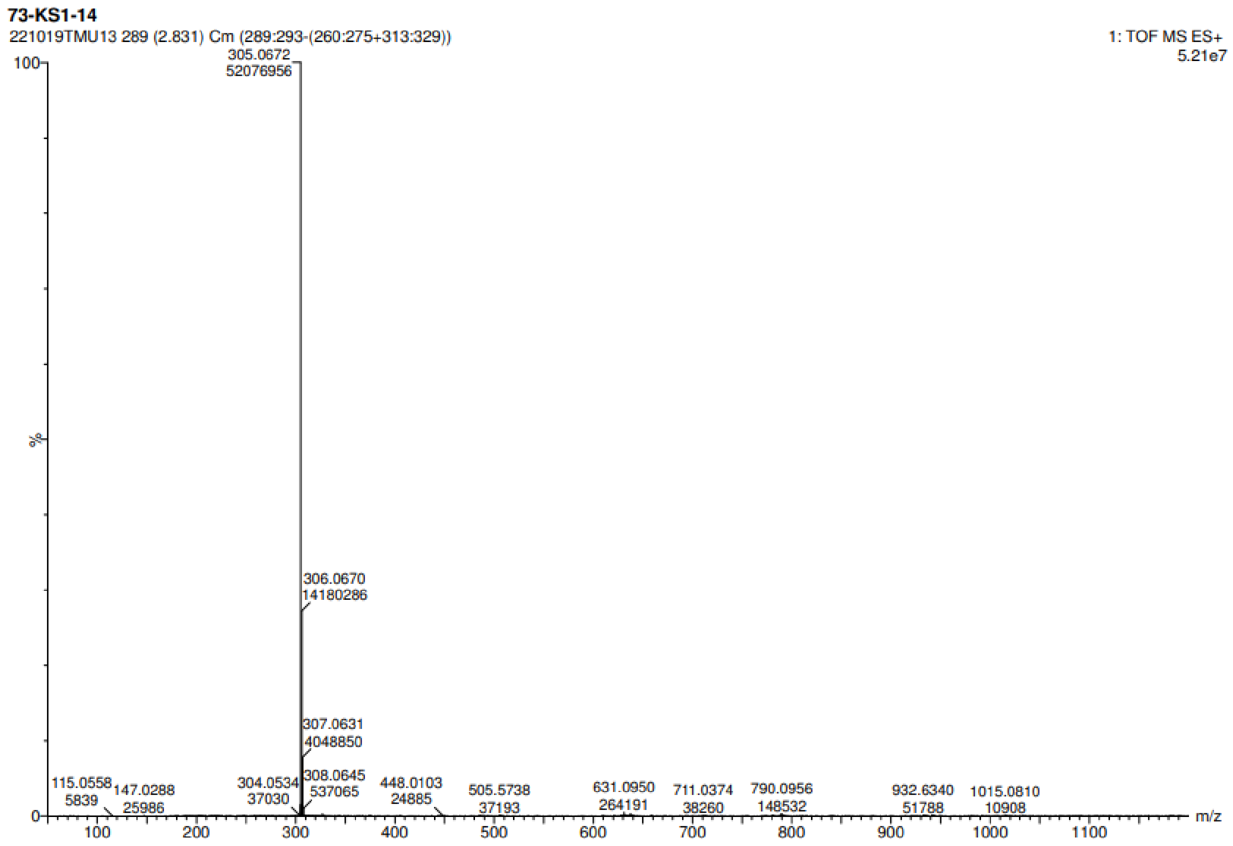

# HRMS Data of Compound 4

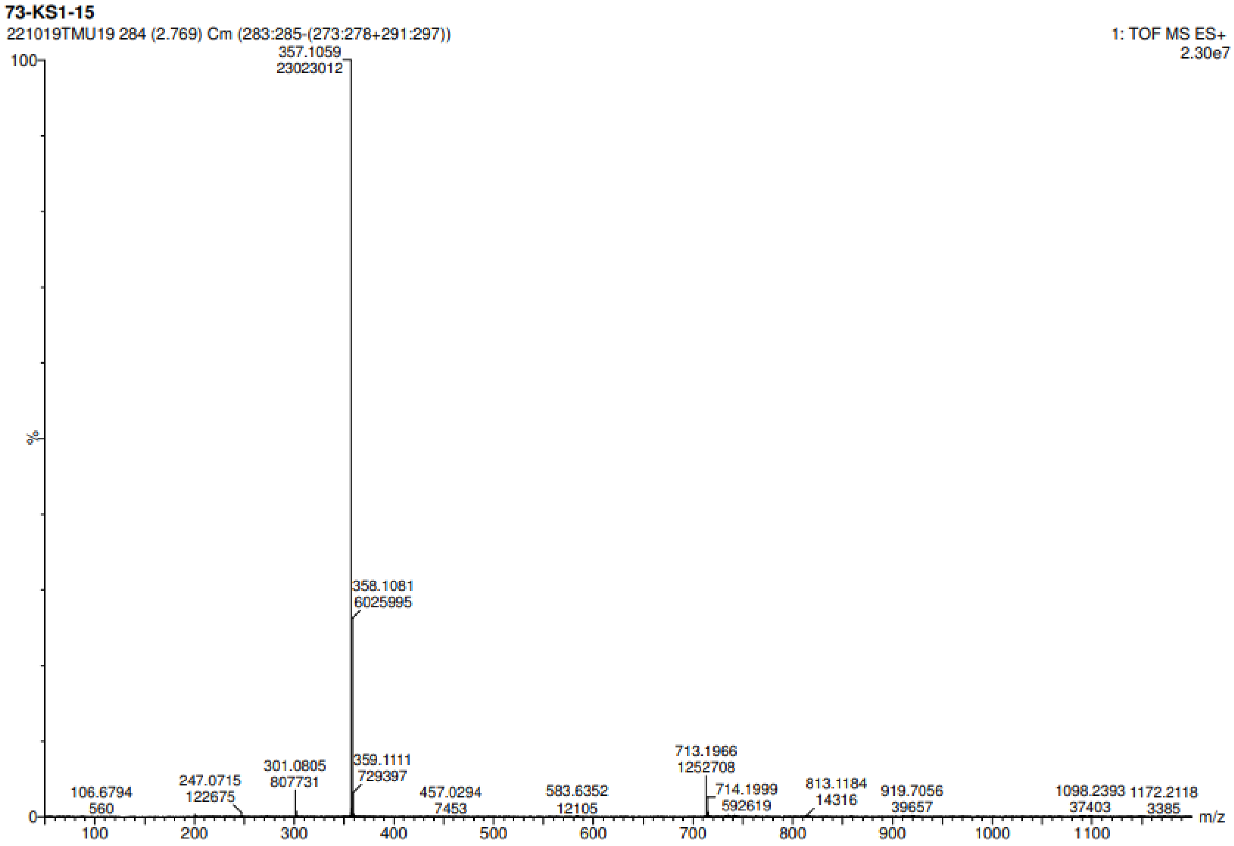

# HRMS Data of Compound 5

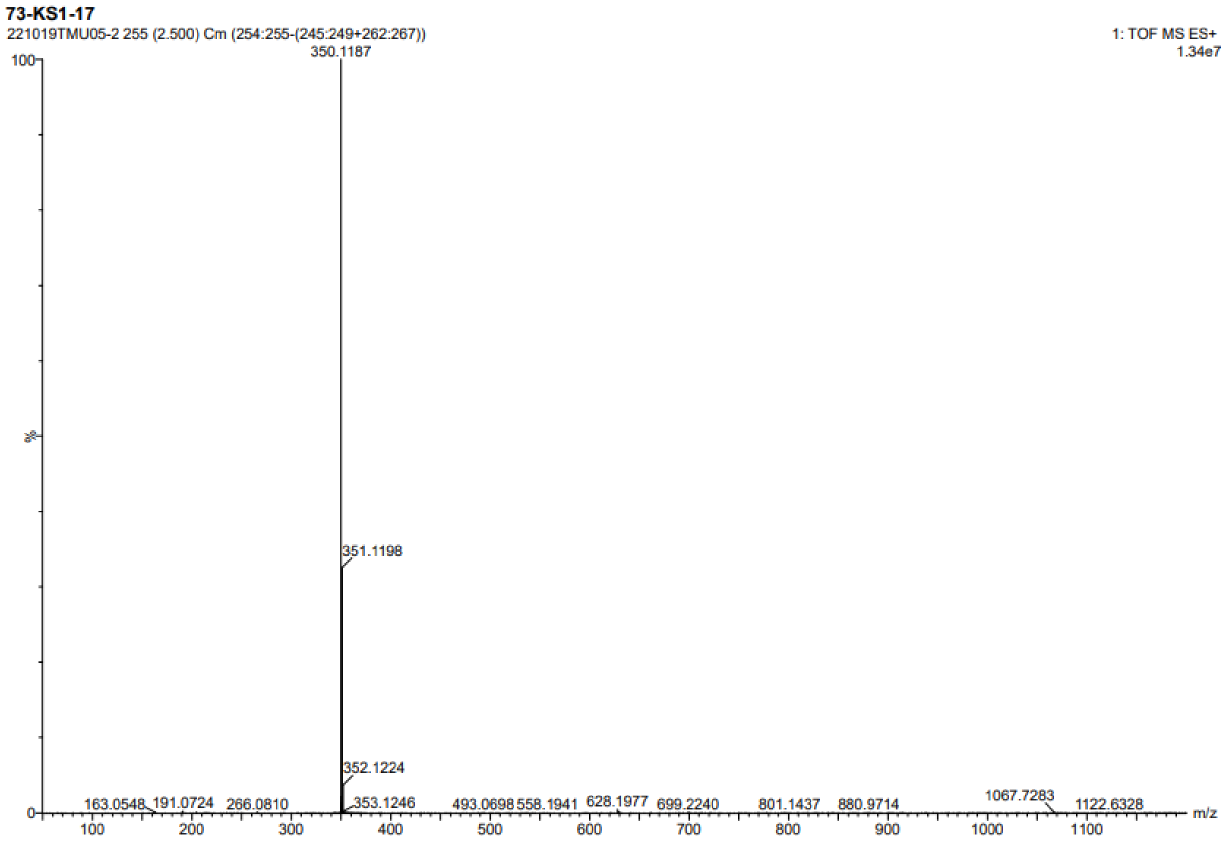

## HRMS Data of Compound 6

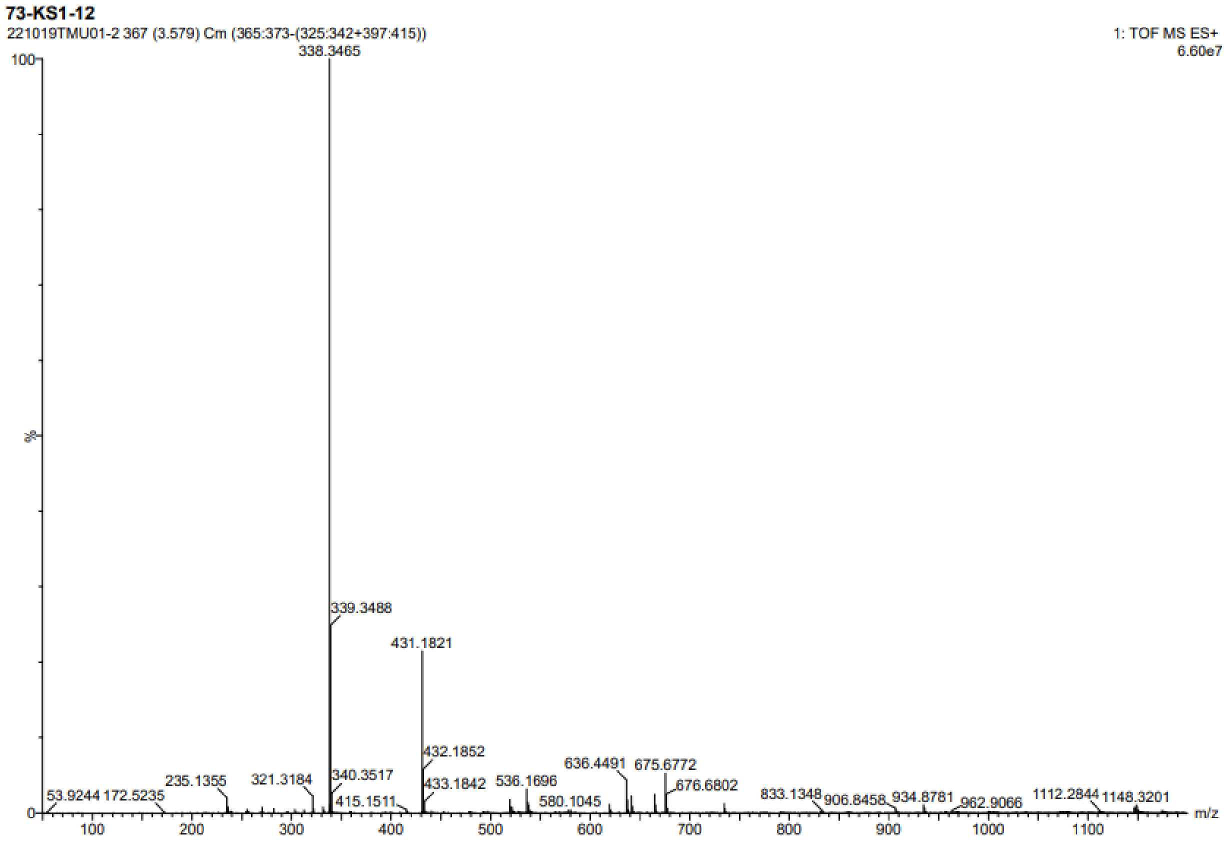

**HRMS Data of Compound 7**

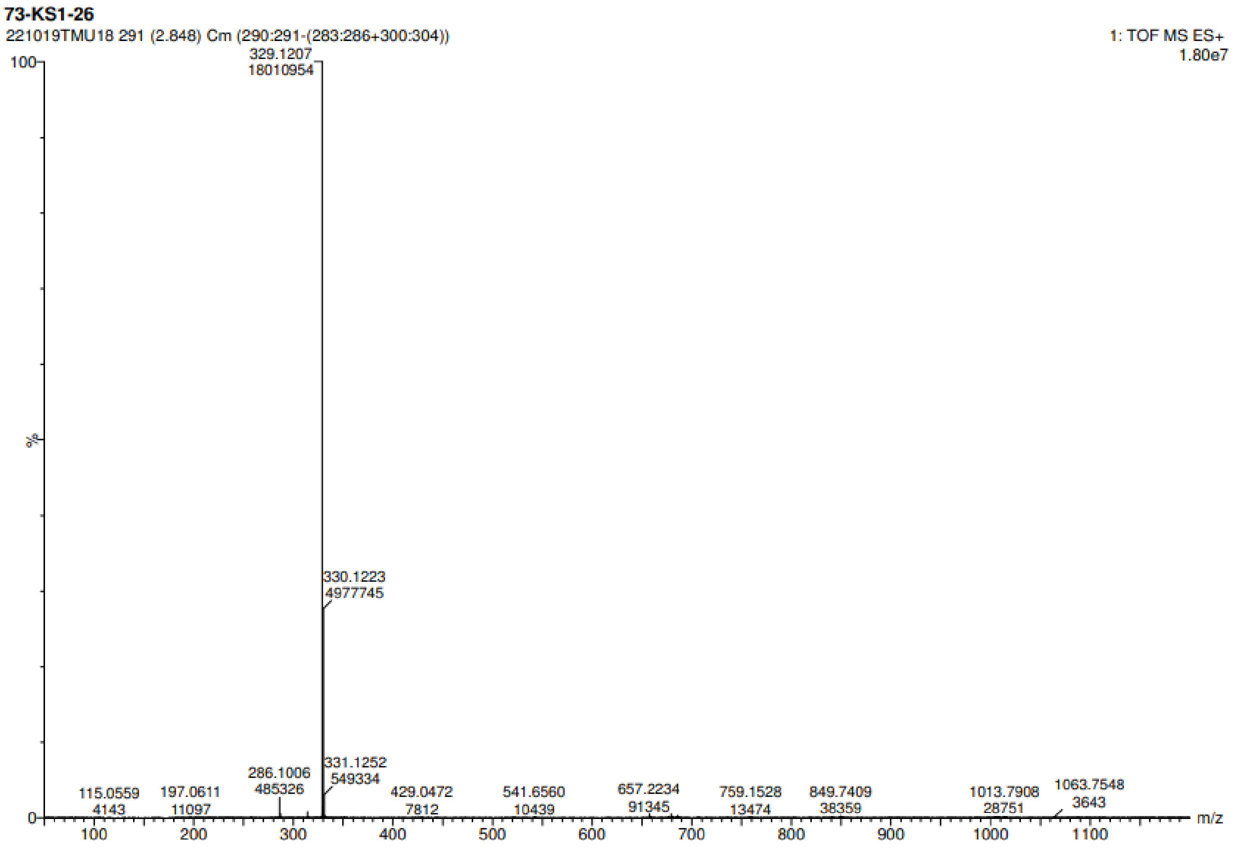

# HRMS Data of Compound 8

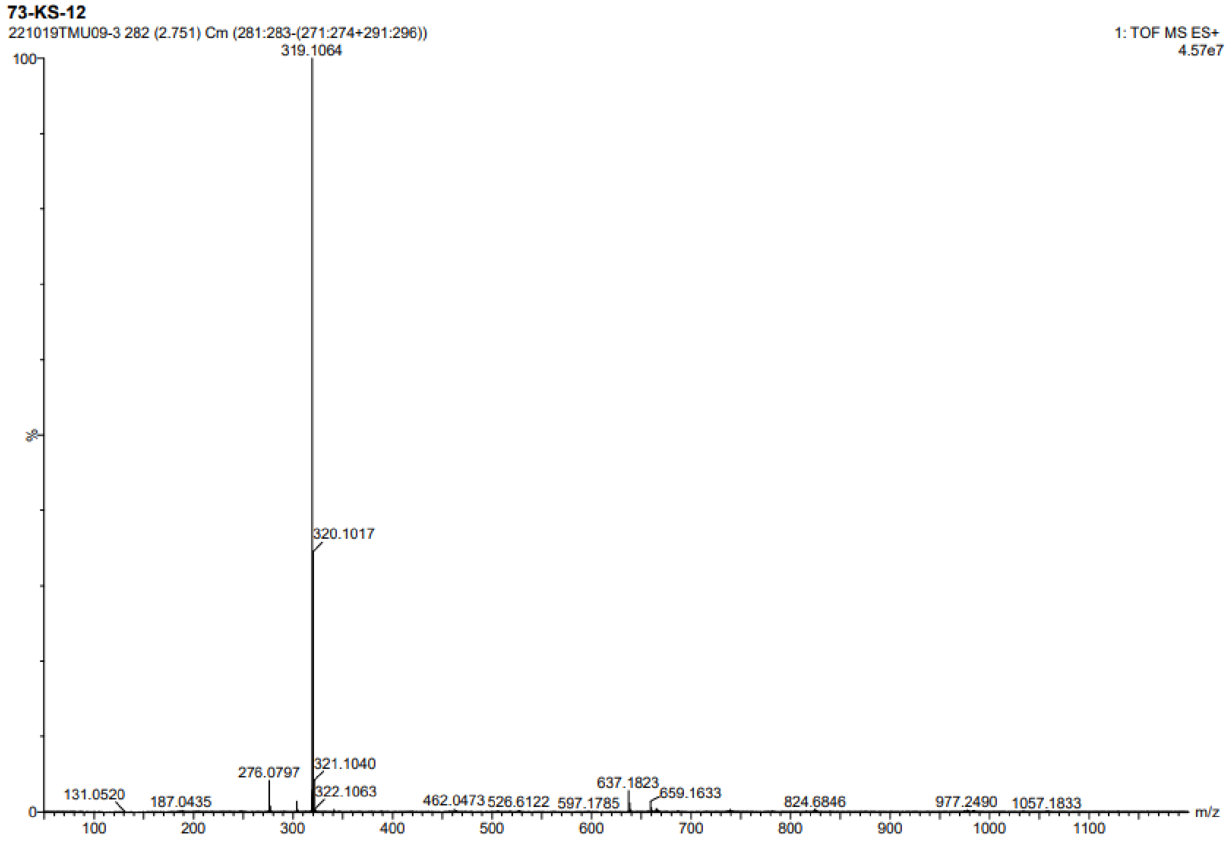

# HRMS Data of Compound 9

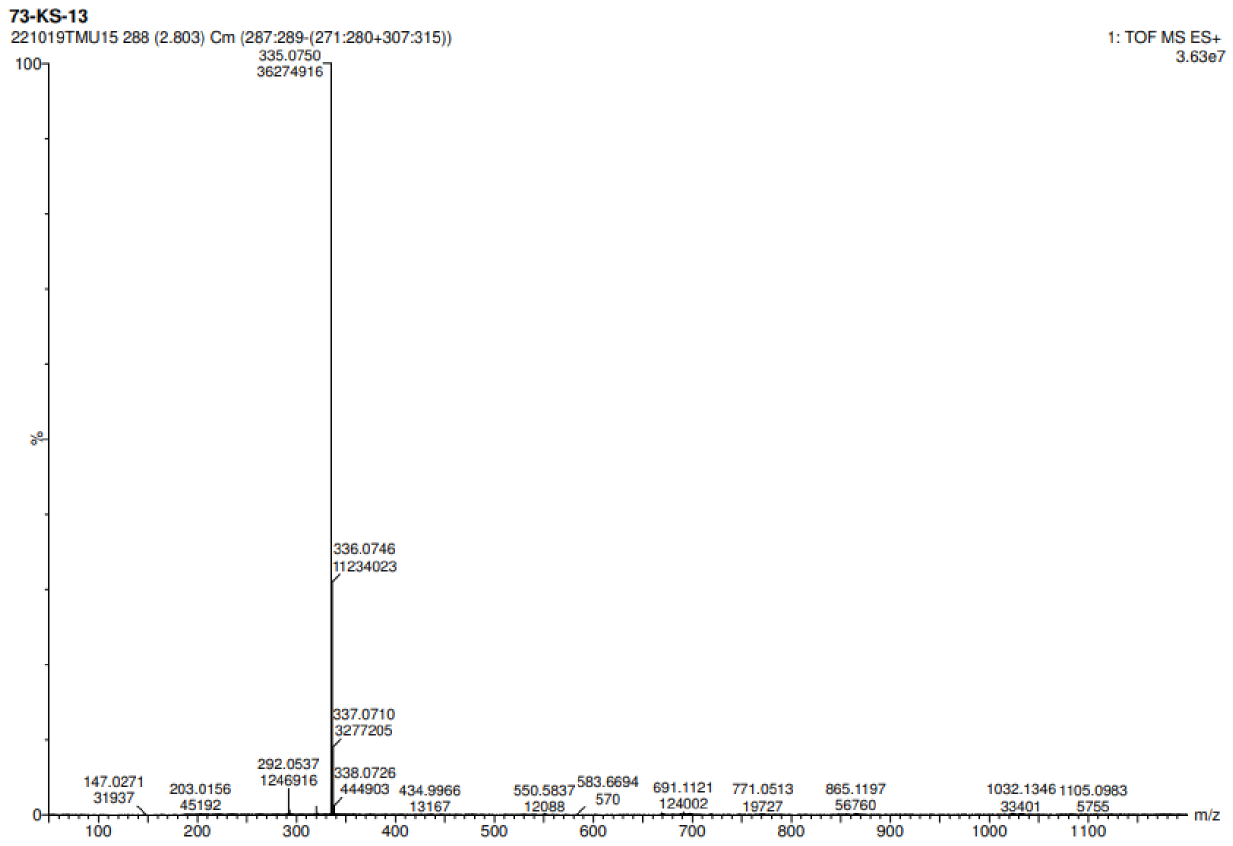

**HRMS Data of Compound 10**

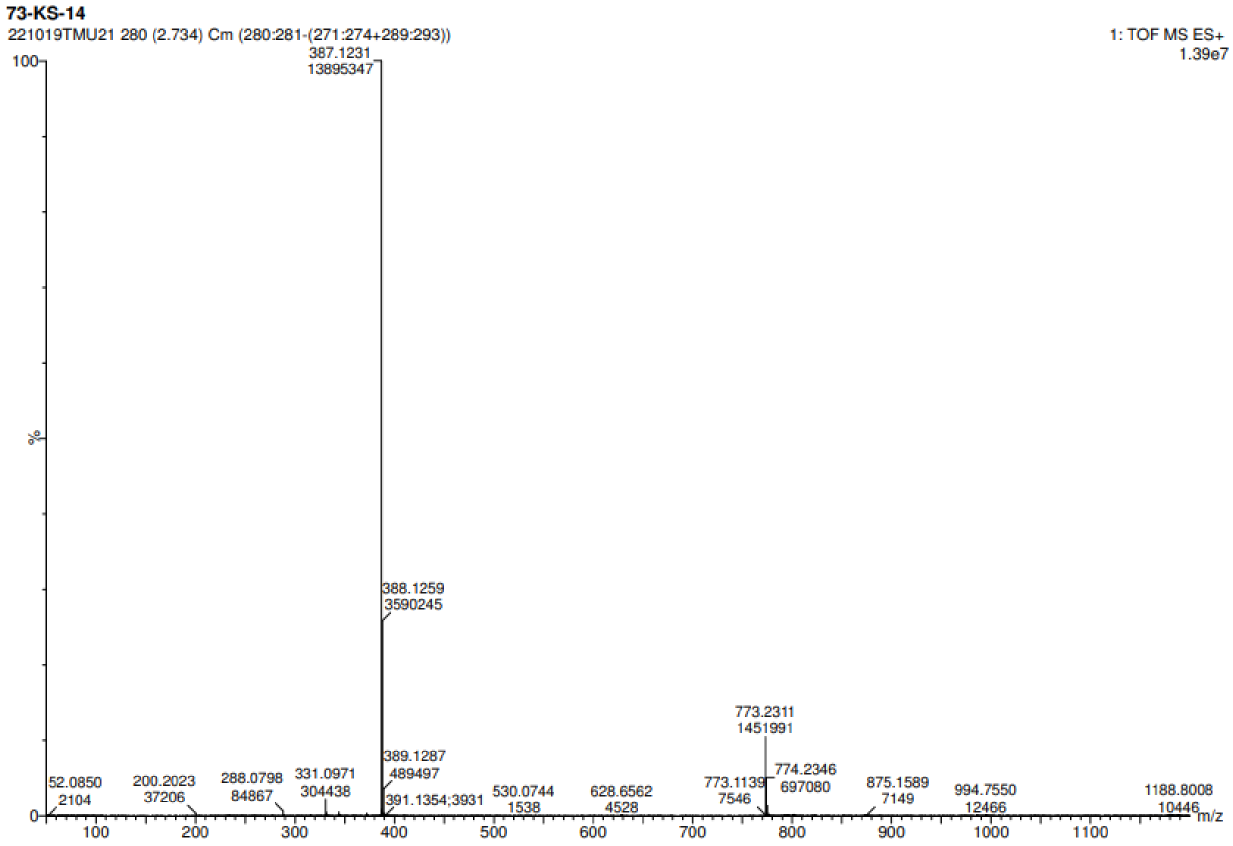

# HRMS Data of Compound 11

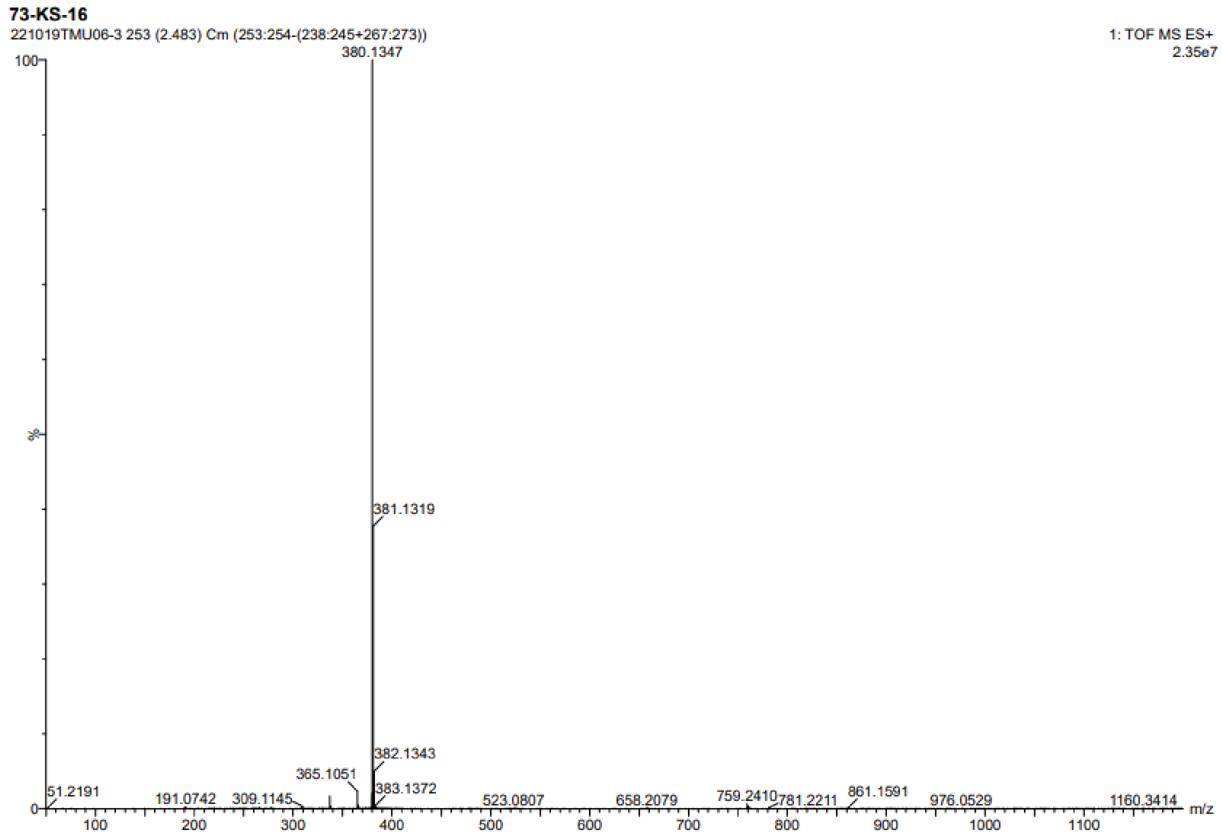

# HRMS Data of Compound 12

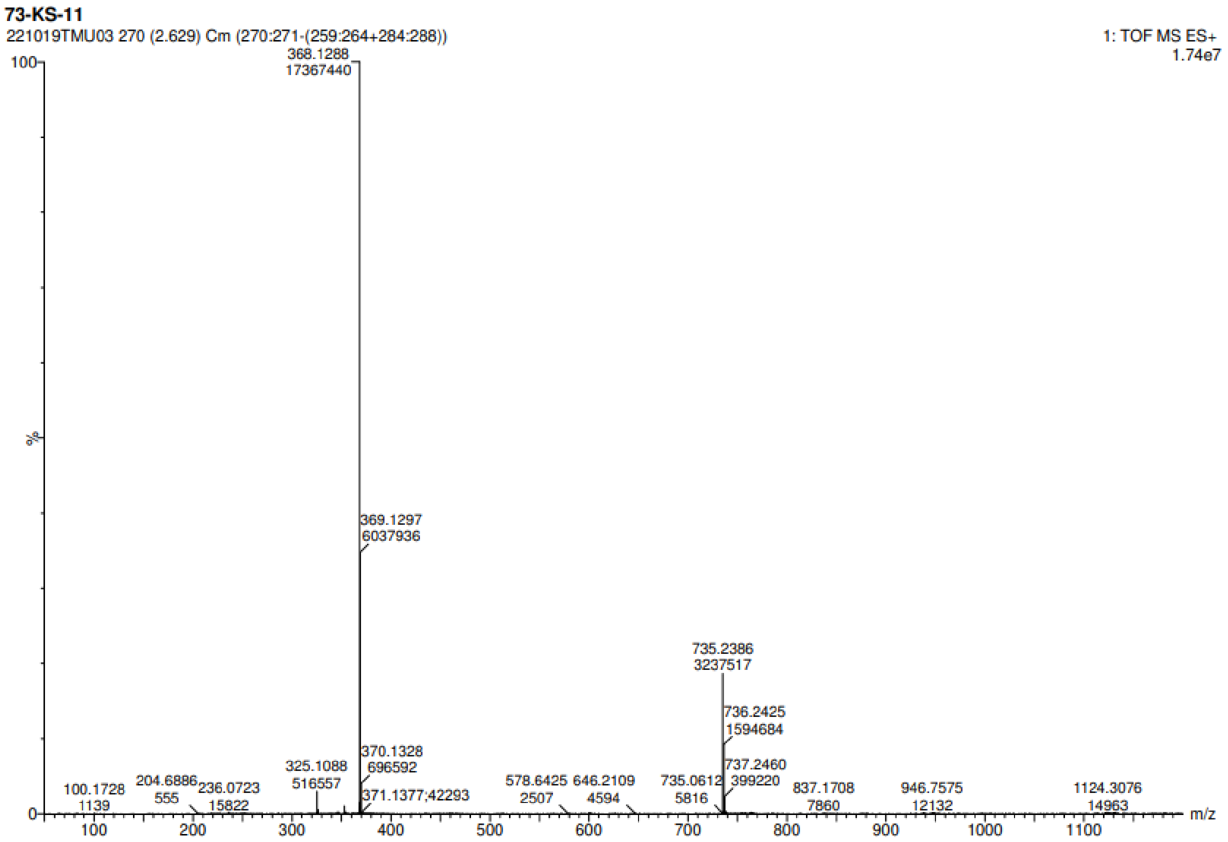

# HRMS Data of Compound 13

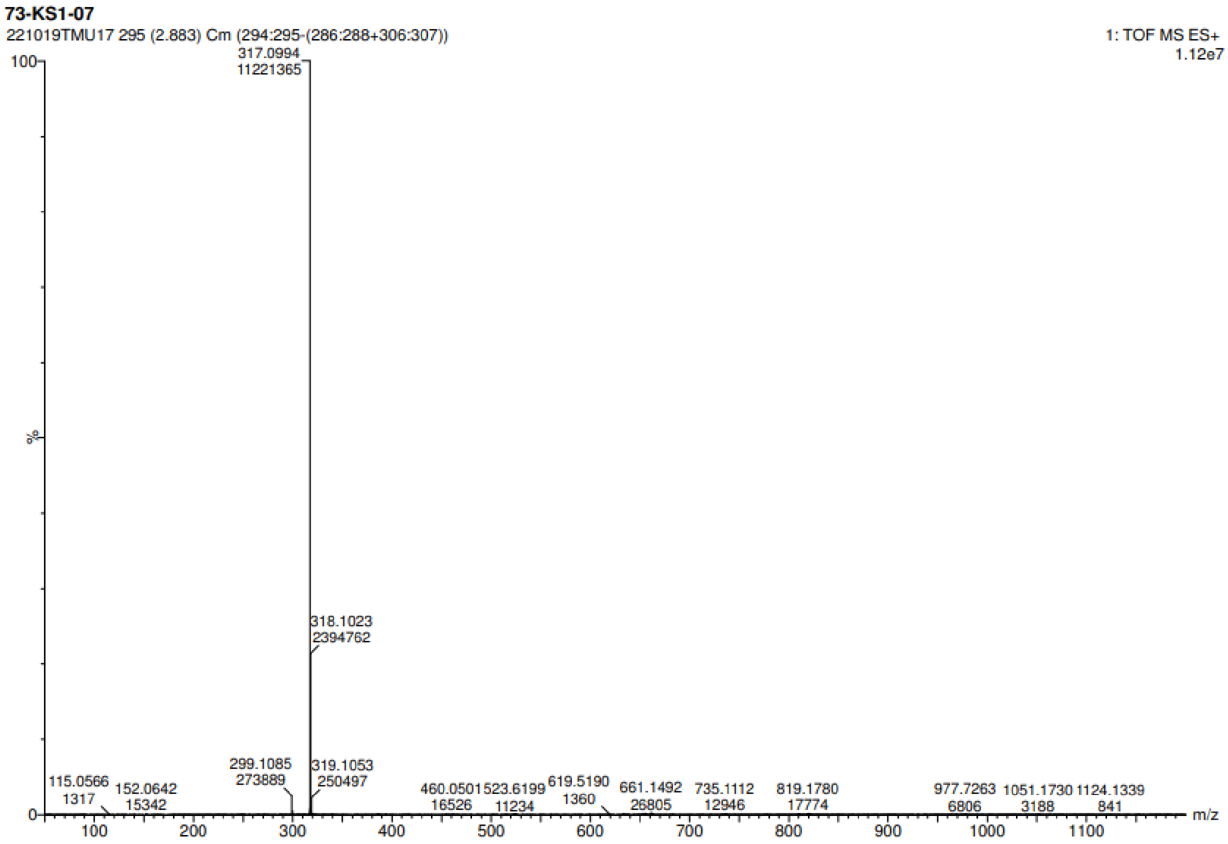

# HRMS Data of Compound 14

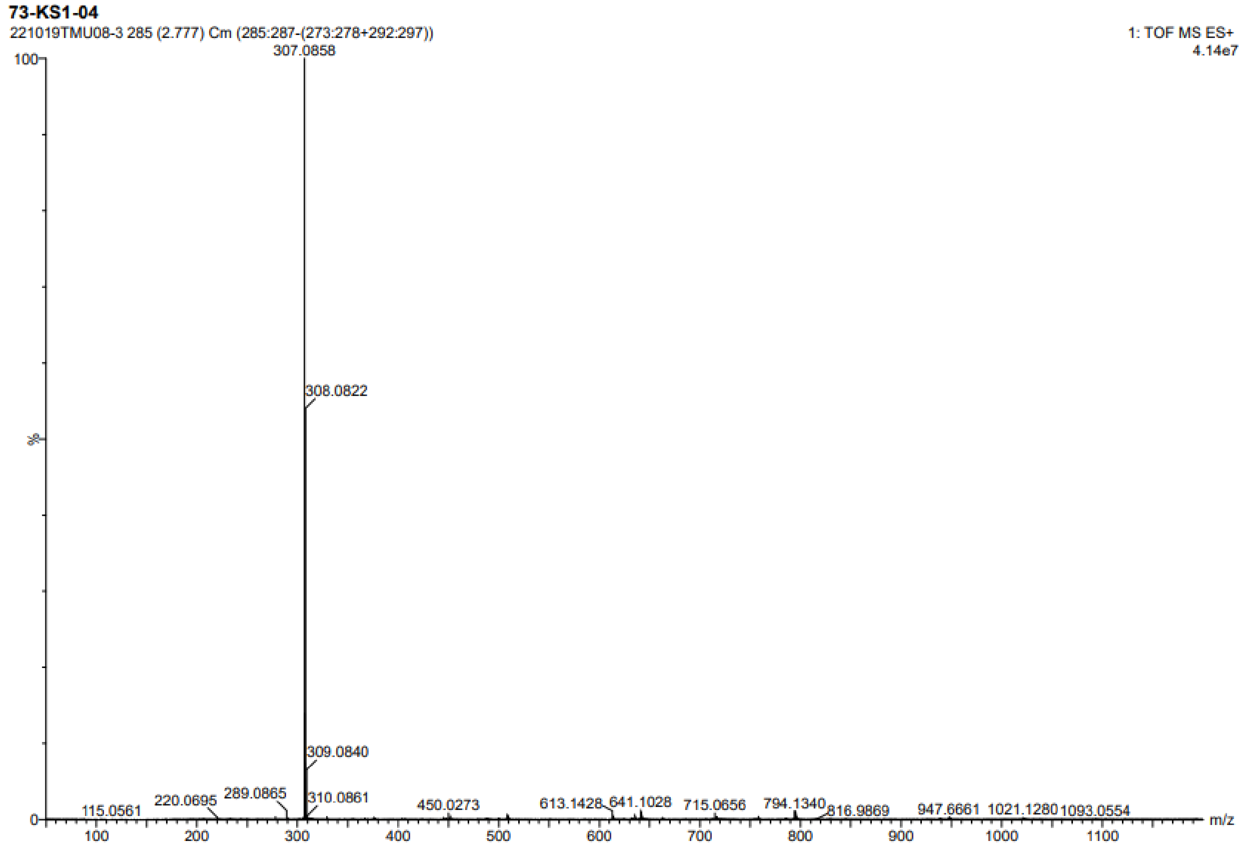

# HRMS Data of Compound 15

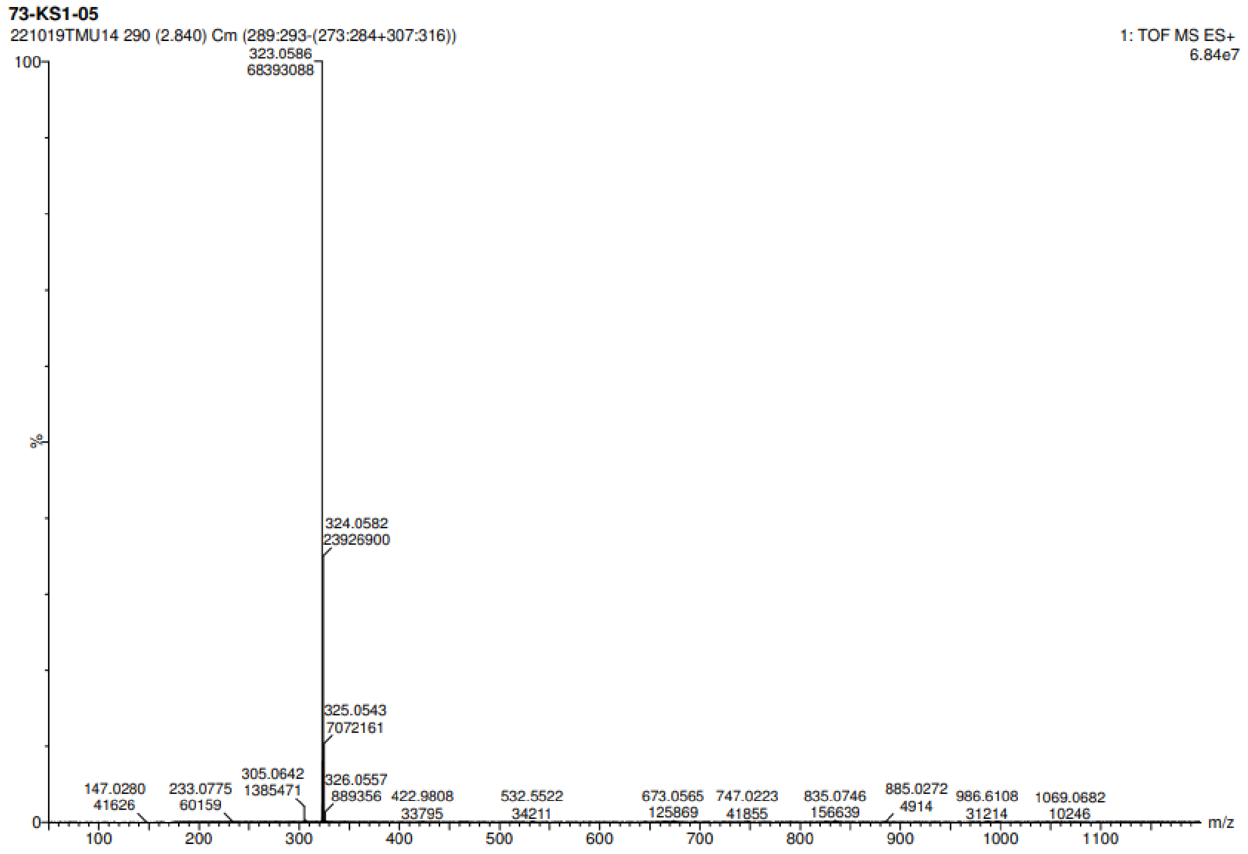

# HRMS Data of Compound 16

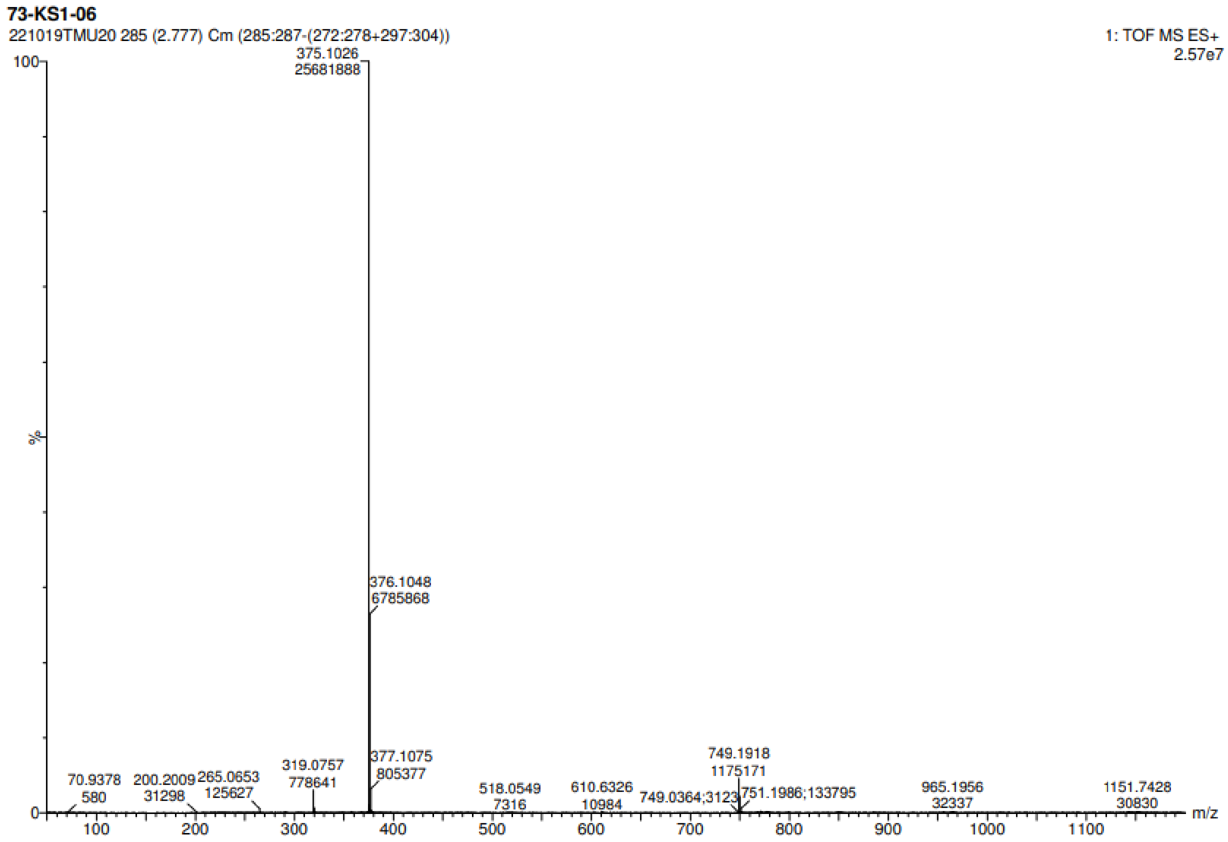

## HRMS Data of Compound 17

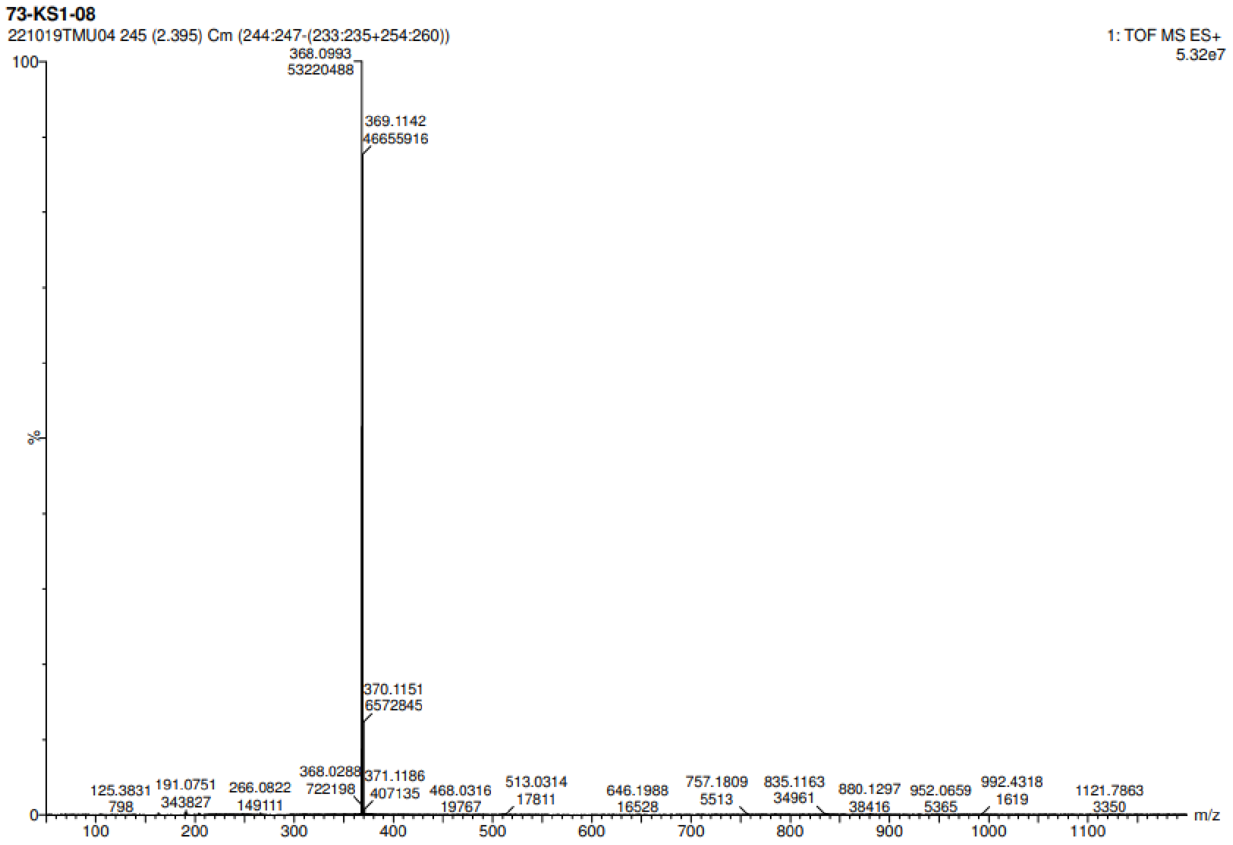

# HRMS Data of Compound 18

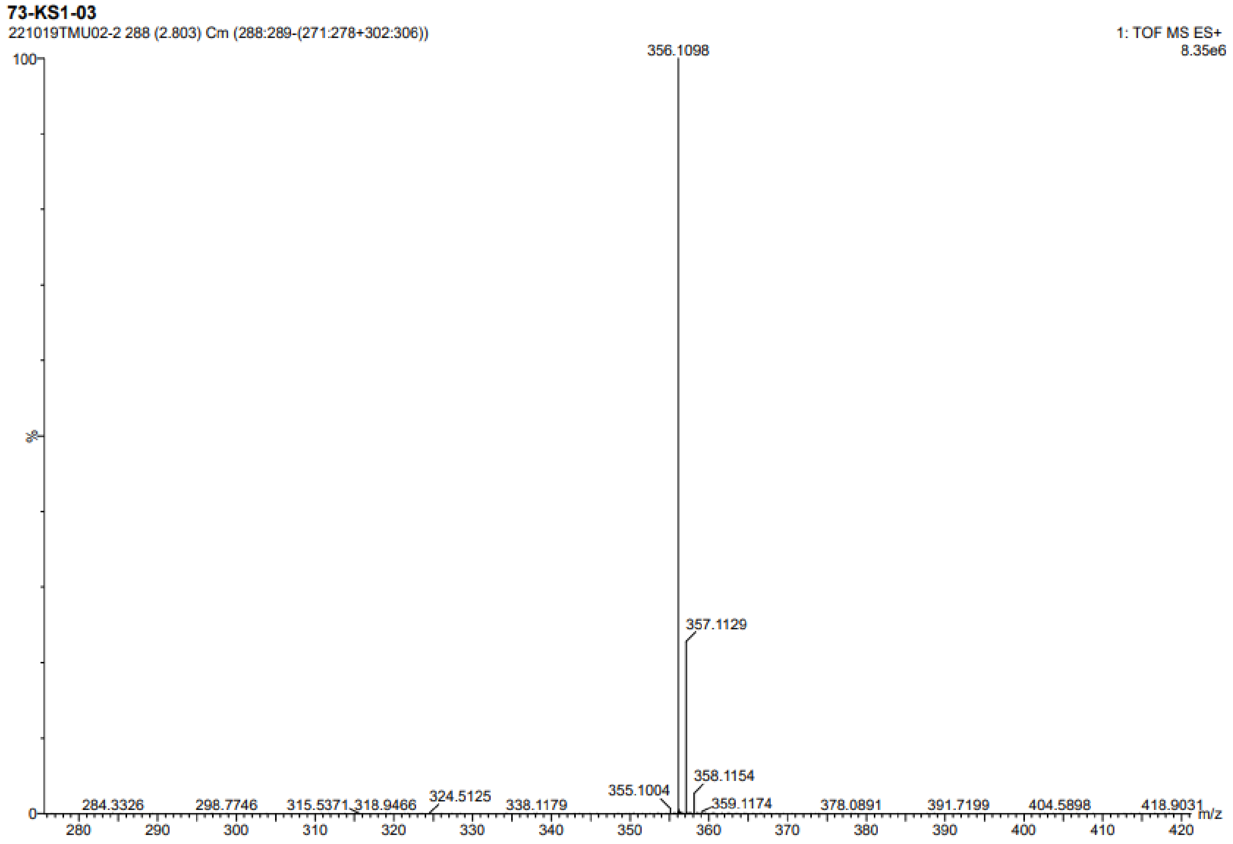

# HRMS Data of Compound 19

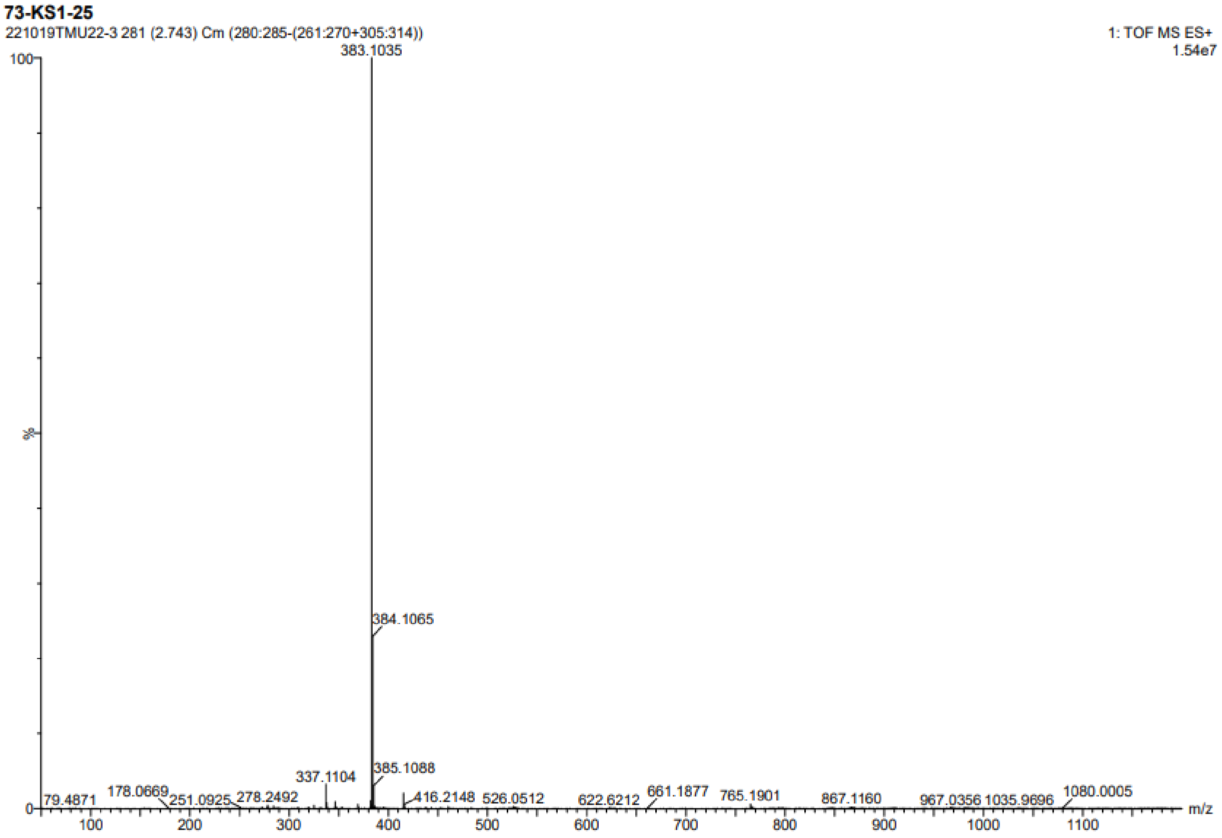

# HRMS Data of Compound 20

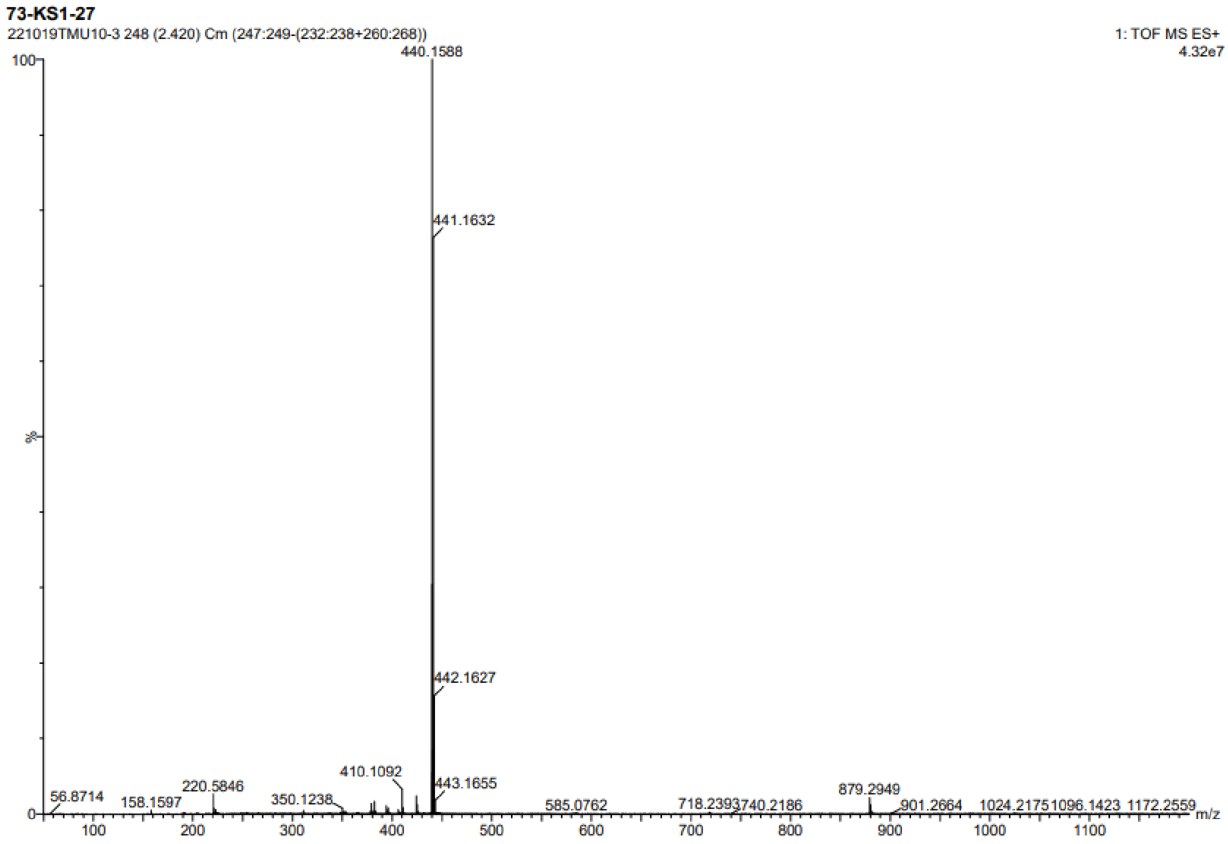

# HRMS Data of Compound 21

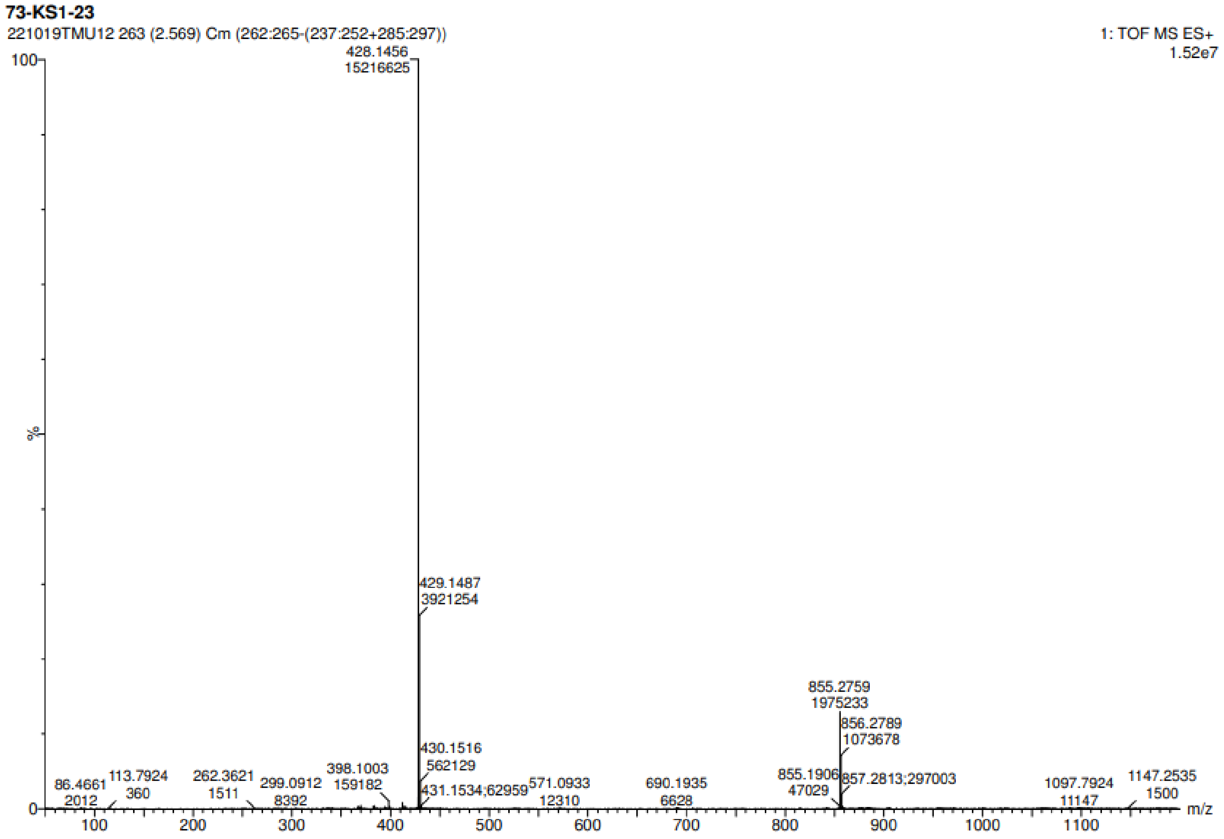

Supplement: Supplemental Material [file IENZ_A_2276665_SM4593.pdf]
